# Integrated Real-World Data Warehouses Across 7 Evolving Asian Health Care Systems: Scoping Review

**DOI:** 10.2196/56686

**Published:** 2024-06-11

**Authors:** Wen-Yi Shau, Handoko Santoso, Vincent Jip, Sajita Setia

**Affiliations:** 1 Regional Medical Affairs, Pfizer Corporation Hong Kong Limited, Hong Kong Hong Kong China (Hong Kong); 2 Transform Medical Communications Limited Wanganui New Zealand

**Keywords:** Asia, health care databases, cross-country comparison, electronic health records, electronic medical records, data warehousing, information storage and retrieval, real-world data, real-world evidence, registries, scoping review

## Abstract

**Background:**

Asia consists of diverse nations with extremely variable health care systems. Integrated real-world data (RWD) research warehouses provide vast interconnected data sets that uphold statistical rigor. Yet, their intricate details remain underexplored, restricting their broader applications.

**Objective:**

Building on our previous research that analyzed integrated RWD warehouses in India, Thailand, and Taiwan, this study extends the research to 7 distinct health care systems: Hong Kong, Indonesia, Malaysia, Pakistan, the Philippines, Singapore, and Vietnam. We aimed to map the evolving landscape of RWD, preferences for methodologies, and database use and archetype the health systems based on existing intrinsic capability for RWD generation.

**Methods:**

A systematic scoping review methodology was used, centering on contemporary English literature on PubMed (search date: May 9, 2023). Rigorous screening as defined by eligibility criteria identified RWD studies from multiple health care facilities in at least 1 of the 7 target Asian nations. Point estimates and their associated errors were determined for the data collected from eligible studies.

**Results:**

Of the 1483 real-world evidence citations identified on May 9, 2023, a total of 369 (24.9%) fulfilled the requirements for data extraction and subsequent analysis. Singapore, Hong Kong, and Malaysia contributed to ≥100 publications, with each country marked by a higher proportion of single-country studies at 51% (80/157), 66.2% (86/130), and 50% (50/100), respectively, and were classified as *solo scholars*. Indonesia, Pakistan, Vietnam, and the Philippines had fewer publications and a higher proportion of cross-country collaboration studies (CCCSs) at 79% (26/33), 58% (18/31), 74% (20/27), and 86% (19/22), respectively, and were classified as *global collaborators*. Collaboration with countries outside the 7 target nations appeared in 84.2% to 97.7% of the CCCSs of each nation. Among target nations, Singapore and Malaysia emerged as preferred research partners for other nations. From 2018 to 2023, most nations showed an increasing trend in study numbers, with Vietnam (24.5%) and Pakistan (21.2%) leading the growth; the only exception was the Philippines, which declined by –14.5%. Clinical registry databases were predominant across all CCCSs from every target nation. For single-country studies, Indonesia, Malaysia, and the Philippines favored clinical registries; Singapore had a balanced use of clinical registries and electronic medical or health records, whereas Hong Kong, Pakistan, and Vietnam leaned toward electronic medical or health records. Overall, 89.9% (310/345) of the studies took >2 years from completion to publication.

**Conclusions:**

The observed variations in contemporary RWD publications across the 7 nations in Asia exemplify distinct research landscapes across nations that are partially explained by their diverse economic, clinical, and research settings. Nevertheless, recognizing these variations is pivotal for fostering tailored, synergistic strategies that amplify RWD’s potential in guiding future health care research and policy decisions.

**International Registered Report Identifier (IRRID):**

RR2-10.2196/43741

## Introduction

### Background

Asia is a vast and diverse continent that also represents varied health care systems and socioeconomic challenges. Multiple evidence-driven approaches tailored to each nation’s unique health care and research context are required to draw essential data to support the ambitious goals for universal health coverage in each country [1,2]. The strength and necessity of real-world data (RWD) and their concrete data analytical interference in terms of real-world evidence (RWE) are integral to this evidence generation. RWE has the potential to inform health technology assessments (HTAs), guide evidence-driven policies, and streamline service delivery [3]. However, as crucial as RWE is, the Asian health care landscape lacks a cohesive framework to harness its full potential despite its promise in pharmacoeconomics, pharmacovigilance, and pharmacoepidemiology [3,4].

The utility of RWD and RWE becomes even more apparent with large integrated research databases within health systems. The integrated warehouses offer vast connected data sets that sustain the statistical rigor and can assist in providing insights with minimal bias and confounding [5]. However, these data reservoirs have not been vastly studied across health care systems, which limits their broader utility in health care research; RWE data generation; and, consequently, universal health coverage [6].

Recognizing this potential, our previous research explored integrated RWD warehouses within 3 diverse health care pilots for Taiwan, India, and Thailand [7]. Our systematic research identified strong differences in the types of RWD and their warehouses in the 3 countries. Still, the results only partly reflected their divergent economic, social, and clinical settings. Hence, we continued to conduct similar research in many other diverse Asian health care systems in line with our published protocol [8].

### Objectives

The literature on RWD practices and awareness of corresponding warehouses in certain Asian countries such as China, Japan, and South Korea is significant [5,9-17], partly because these countries also have recommendations on the utility of RWE by external regulators [3]. This study sought to understand the evolving landscape of RWD use and its implications across Hong Kong, Indonesia, Malaysia, Pakistan, the Philippines, Singapore, and Vietnam, where RWD practices are emerging or undergoing significant development [4]. Our selection of countries for this scoping review was strategically based on selecting a contrasting spectrum of HTA maturity across countries with evolving HTA systems, ranging from relatively mature systems in Singapore, Thailand, and Malaysia to emerging frameworks in Indonesia, the Philippines, and Vietnam and systems in the nascent stages in Pakistan [18,19]. Each nation, with its individualistic health care challenges and unique research capabilities, underscores the need for understanding recent patterns in RWD research and use of clinical research warehouses, especially in light of the marked underrepresentation of specific Asian demographics in traditional randomized clinical trials [20]. By systematically analyzing both single-country studies (SCSs) and cross-country collaboration studies (CCCSs), this research aimed to delineate the current state of RWE generation and collaborative research initiatives for RWE from integrated databases across different nations in Asia. Our objectives also included obtaining a comprehensive understanding of the preference for RWD methodologies by contrasting the emphasis on comparative effectiveness research (CER) with descriptive studies and discerning the preferred and popular real-world research databases.

The cyclical interplay between a nation’s economic strength, health care infrastructure, and research capacity perpetuates disparities in RWD generation. We hypothesized that Asian countries with less extensively documented RWD research trends could be effectively clustered based on systematic patterns in RWD generation. This streamlined our objective to evaluate trends in RWD generation and shed light on targeted capacity-building strategies essential for informed health care policy making. Through this rigorous extended scoping research, we aimed to present insights that resonate with clinical stakeholders, medical researchers, and health policy makers, thereby guiding the formulation of strategies attuned to each nation’s health care challenges and research diversities and complexities.

## Methods

### Research Approach

Our research approach was methodically aligned with the guidelines set forth by the PRISMA-ScR (Preferred Reporting Items for Systematic Reviews and Meta-Analyses extension for Scoping Reviews) [21]. Our published protocol specified a preliminary focus on 3 countries—India, Thailand, and Taiwan—as a representative pilot to explore the diversity of health care systems and RWD use in Asia [8]. The outcomes from our initial study covering Taiwan, India, and Thailand have been previously published [7]. Relevant insights from the latter publication were incorporated into the archetyping of the nations wherever applicable. In this study, we expanded our protocol to 7 other countries. However, we maintained consistency with the original protocol’s methodological framework to ensure comparability across all countries studied. This expansion was aligned with our initial intent to potentially include more countries following the first study across 3 countries.

The search strategy is described in Table S1 in Multimedia Appendix 1. We filtered our search to include only English-language publications from the last 5 years, aiming to highlight current and internationally relevant RWE or RWD. As the conversion of RWD to RWE emphasizes the stringent analytical processes necessary to yield valuable and credible findings, we intentionally chose to rely on PubMed as an exclusive source of relevant citations for screening. Our goal was to assess studies yielding robust RWD featured in esteemed, indexed, and peer-reviewed journals while reducing potential duplicates. By focusing solely on PubMed, we tried to identify research representing this standard and offering evidence of the utmost scientific integrity. This strategy aligns with the specifications outlined in our protocol [8].

### Screening Eligible Studies for Data Analysis

All retrieved study abstracts were directly imported into the Covidence software (Veritas Health Innovation) for subsequent screening and data extraction. Studies were initially screened against predefined eligibility criteria to capture research from integrated RWD. The criteria encompassed 4 domains described in the original protocol: database type and requirement for research across >1 hospital or clinic, publication nature, RWD study type, and publication scope [8]. The scope of publication was adapted in this study to include citations on databases involving 1 of the target nations (Hong Kong, Indonesia, Malaysia, Pakistan, Philippines, Singapore, or Vietnam). The inclusion criteria also considered studies featuring nontarget countries as long as 1 of the 7 target nations was involved. Table S2 in Multimedia Appendix 1 provides a snapshot of the eligibility criteria used in this study.

Duplicate removal and a 2-step eligibility screening process were conducted in Covidence. The initial step (phase 1) assessed titles and abstracts, with relevant studies advancing to full-text evaluation in the second phase. Given the study volume, the screening for both phases was divided between 2 reviewers. An independent reviewer examined a random sample of 20% of the studies to maintain accuracy. Any ambiguities or discrepancies were collaboratively resolved, and another reviewer was consulted if needed. The final step involved data extraction and data analysis for eligible studies.

### Data Extraction and Analysis

We used Covidence for data extraction through a custom template that covered the following:

1. Basic study details: Covidence identifier (ID) based on the first author’s last name and publication year and title.
2. Presence of cross-country collaboration in the research (CCCS or SCS).
3. Nature of publication (clinical study or protocol).
4. Study categorization: CER versus descriptive study (non-CER), with CER definitions adapted from Medical Subject Headings. We expanded the criteria for CER to standardize its meaning in the context of this research as the “studies comparing interventions and strategies (including the comparison between active and nonactive interventions and strategies) to prevent, diagnose, treat, and monitor health conditions using validated methods for confounders elimination, e.g., matching, and statistical adjustments like stratification, weighting, regression, instrumental variable analysis etc” [7].
5. Research source database classification involving medical records, health insurance claims, clinical registries, pharmacy claims, or composite databases.
6. Disease specifics: name and area of the target disease under study (defined by primary diagnosis and pathophysiology or by prime medical specialty in charge if they intersected). The disease categories encompassed cardiology and metabolic disorders (CVM), oncology, inflammatory and autoimmune disorders, infectious diseases and vaccines (IDV), and others. These categories represent major research fields in clinical medicine with significant disease burdens, selected to provide pertinent insights into RWD and RWE applications within these critical domains.
7. Outcome types: clinical (treatment effect or safety), cost, or patient-reported outcomes (PROs), with PROs capturing direct patient responses.
8. Demographics (adults, children, or both), number of centers, study participants, and length of study and duration between last data collected and year of publication. The length or duration represented the span from the study’s commencement to completion as specified by the authors.
9. The unique names for the databases used. When provided, the name of the specific database used in each study was collected and organized by target nation, database type, and disease area.

A total of 2 reviewers collaboratively managed data extraction, and all extractions underwent quality checks by another reviewer to ensure the accuracy and reliability of the extracted data. However, this process was not conducted independently or blinded to the other reviewer’s decisions. Disagreements between the 2 reviewers were settled through discussions, and an additional reviewer was involved whenever there was a need for a consensus.

The final search was conducted on May 9, 2023, covering the preceding 5 years; to account for partial yearly data in 2018 and 2023, we calculated the equivalent of annual publication count using 365 multiplied by the average daily number of publications. We used linear regression, using the year as a continuous predictor variable, to understand the annual trend in nation study counts. This provided insights into the average annual trend in study numbers throughout the search period. To further even out year-to-year variations, a 2-year simple moving average (SMA) was applied to enhance the clarity of the data trends. This SMA approach was consistent with our previous research methodologies [7]. Given the study’s descriptive nature, there was no a priori statistical hypothesis. Statistical analyses were conducted to calculate point estimates and their associated errors. Categorical data were presented as frequencies and percentages, whereas continuous data were presented as means and SDs. We used Microsoft Excel (Microsoft Corp) for all data analyses. Adobe Illustrator was used for crafting high-definition figures for the main manuscript.

## Results

### Eligible Studies

**Figure 1:**
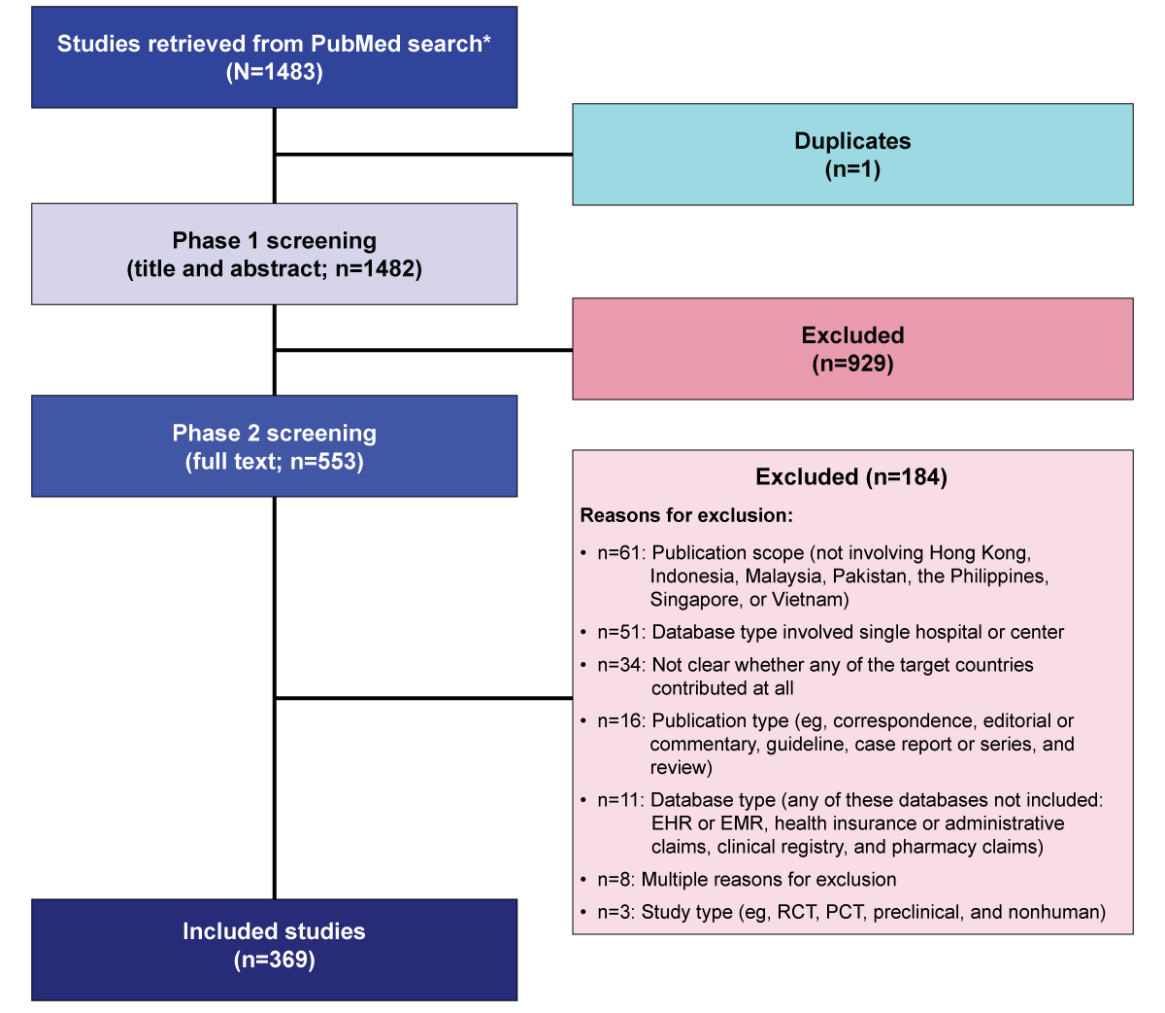
PRISMA (Preferred Reporting Items for Systematic Reviews and Meta-Analyses) flowchart for the selection of eligible studies. *Search date: May 9, 2023. EHR: electronic health record; EMR: electronic medical record; PCT: pragmatic clinical trial; RCT: randomized clinical trial.

The search was conducted on May 9, 2023, and yielded 1483 studies with 1 duplicate. Of these 1483 studies, 553 (37.3%) were included in phase-2 screening, and 369 (24.9%) studies were eligible for data extraction (Figure 1).

The vast majority of the publications (361/369, 97.8%) were original research, whereas the remaining 2.2% (8/369) were study protocols. The country-wise distribution of SCSs and CCCSs is illustrated in Multimedia Appendix 2.

### Geographic Distribution and Collaboration Relationship

**Figure 2:**
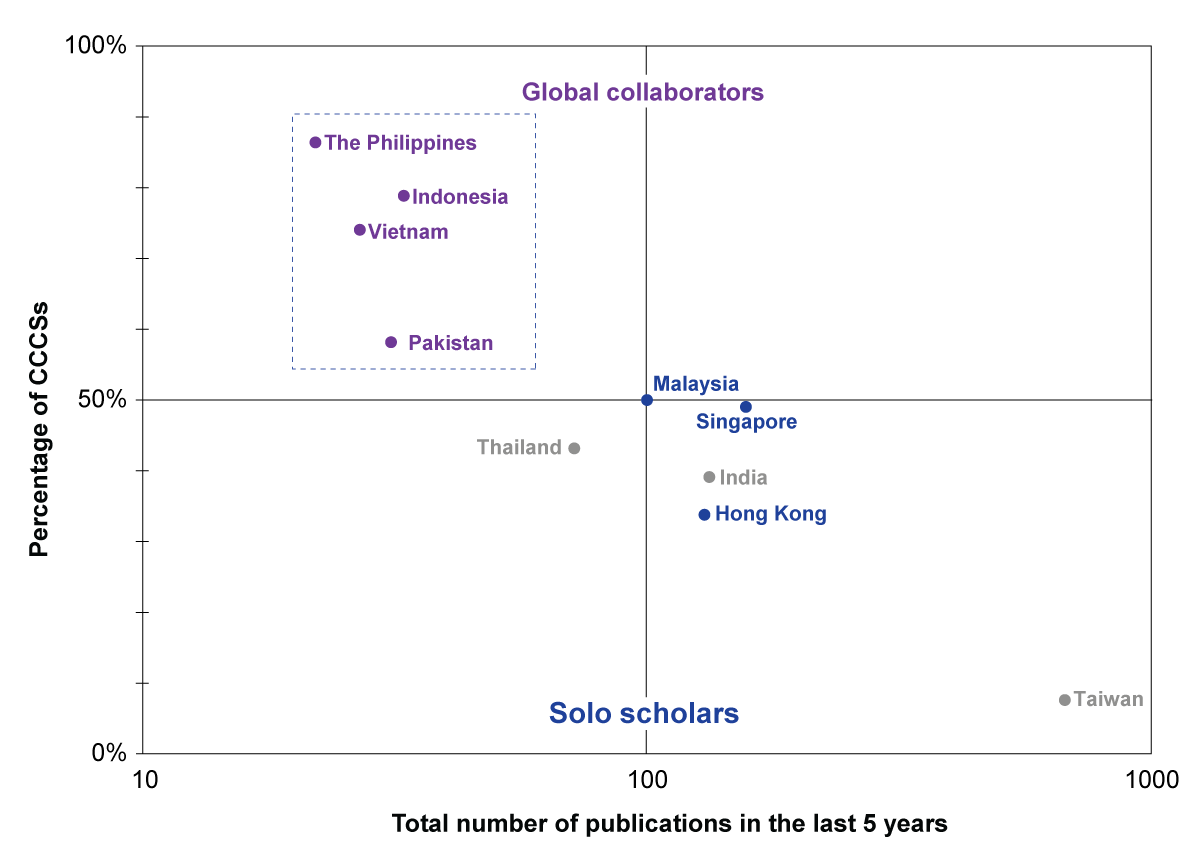
Archetype by number of publications and percentage of cross-country collaboration studies (CCCSs) from real-world study databases in the last 5 years from 10 nations in Asia. Scatter plot archetyping for countries with a relatively high percentage of CCCSs but fewer total publications as global collaborators (highlighted within the dashed lines). Solo scholars cluster includes countries with a lower percentage of CCCSs (≤50%) and a higher number of publications (≥100 real-world data publications from integrated databases in the last 5 years). Countries such as Taiwan show a high number of total publications with a relatively low percentage of CCCSs, signifying a tendency to conduct independent research. The data for India, Taiwan, and Thailand were derived from our previous publication that used the same methodology as that used in this research [7].

Among the 369 studies that qualified for data extraction, Singapore, Hong Kong, and Malaysia each contributed to ≥100 publications, with respective counts of 157 (42.5%), 130 (35.2%), and 100 (27.1%). The other 4 nations—Indonesia, Pakistan, Vietnam, and the Philippines—were involved in fewer publications, with 8.9% (33/369), 8.4% (31/369), 7.3% (27/369), and 6% (22/369), respectively, and each had >50% of their studies classified as CCCSs (Table S3 in Multimedia Appendix 1). Given their lower overall study numbers and the predominance of CCCSs, these 4 nations were categorized as *global collaborators* in certain subsequent analyses (Figure 2 [7]).

Countries beyond the 7 target nations of this study that were involved in collaborations were labeled as nontarget countries. The cross-country collaboration network across the 7 target nations and nontarget countries is described in Table S4 in Multimedia Appendix 1. The average number of collaborative countries (ANC) indicates the cross-country interconnection for research of a given nation. The ANC varied from 2.2 for Singapore to 4.1 for the Philippines. Despite having the lowest ANC, Singapore was involved in 30.3% (77/254) of the studies, making it the highest contributor to CCCSs. On the other hand, Malaysia, with a higher ANC of 3.1, participated in 50% (50/100) of CCCSs, making it the most engaged collaborator within the *solo scholars* cluster. Malaysia participated in 56% (10/18) of CCCSs from Pakistan, 75% (15/20) of CCCSs from Vietnam, 81% (21/26) of CCCSs from Indonesia, and 84% (16/19) of CCCSs from the Philippines.

Nontarget countries were common collaborators in CCCSs across all 7 target countries, with their involvement ranging from 84% (16/19) to 98% (43/44).

### Time Trend

**Figure 3:**
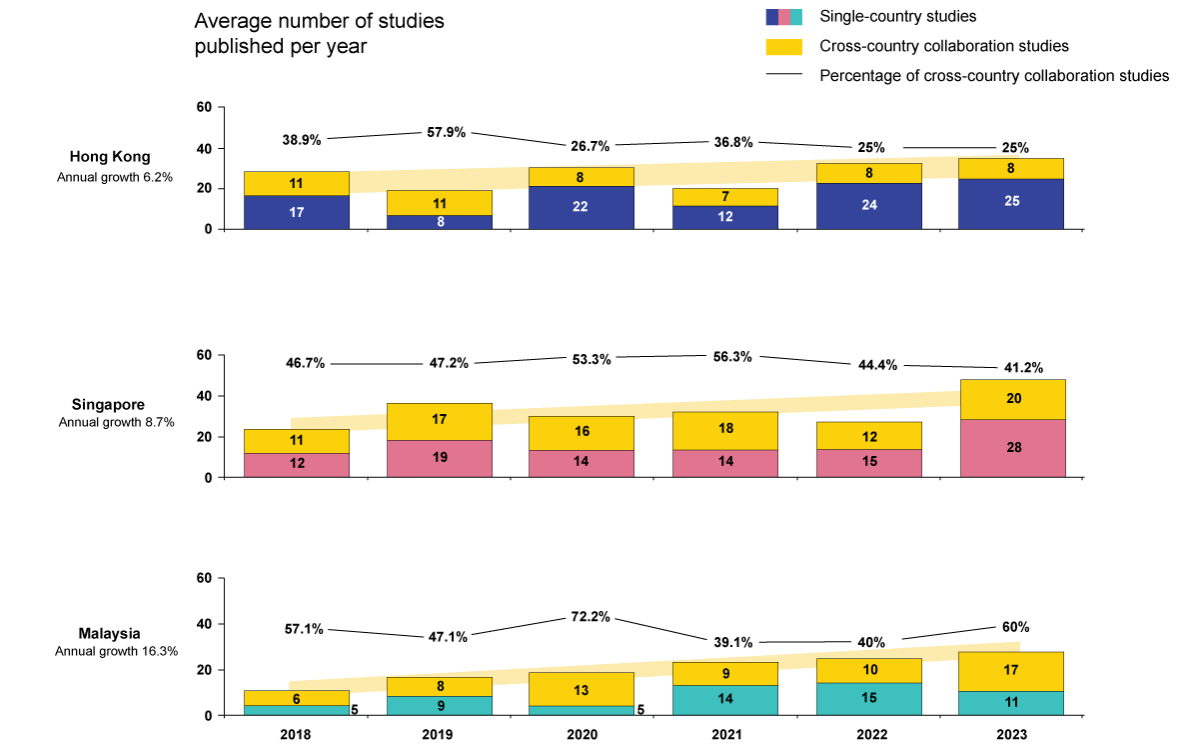
Annual trends in average number of publications by geographical distribution for solo scholar nations. Malaysia showed the highest annual growth rate of 16.3% among the solo scholars cluster, followed by Singapore (8.7%) and Hong Kong (6.2%).

**Figure 4:**
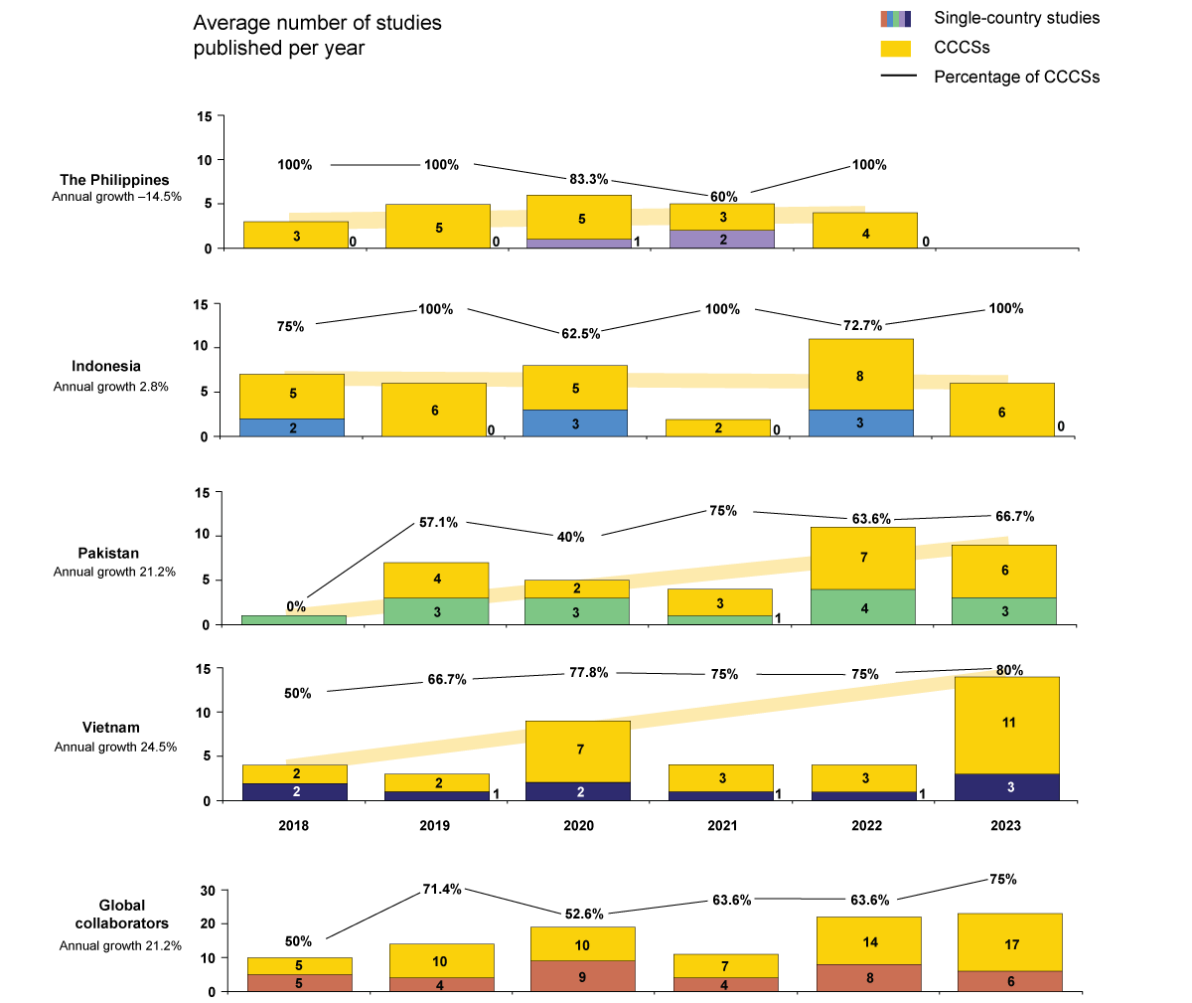
Annual trends in average number of publications by geographical distribution for global collaborator nations. Vietnam exhibited the most significant increase in total studies among the 7 target countries, followed by Pakistan and Malaysia. The Philippines was the only nation with a decline, whereas Indonesia and Hong Kong maintained a consistent study count. Vietnam and, to a lesser degree, Indonesia and Pakistan demonstrated a rising participation in cross-country collaborative research. Duplicate studies within the cluster were adjusted, and thus, the sum of cross-country collaboration studies (CCCSs) from all 4 nations might be larger than the number of CCCSs in the cluster as whole.

Figures 3 and 4 depict the yearly average counts and growth rates of SCSs and CCCSs across the 7 target nations from the *solo scholar* and *global collaborator* clusters, respectively. Between 2018 and 2023, every target nation except for the Philippines, which experienced a decline of –14.5%, exhibited an upward trend in the average number of studies published. Vietnam led with the steepest growth rate at 24.5%, trailed by Pakistan at 21.2%. Among solo scholars, there were growing trends for all 3 nations, with a growth rate of 6.2% for Hong Kong, 8.7% for Singapore, and 16.3% for Malaysia. Due to the small average number of studies by year in some of the individual global collaborator nations, growth trends are also presented collectively for global collaborators as a cluster in Figure 4. Duplicate studies within clusters were adjusted; thus, the sum of CCCSs from all 4 nations might be larger than the number of CCCSs of the cluster as whole. The growth rate was 21.2% in the global collaborators cluster after adjusting for duplicates.

### Overall Attributes

#### Overview

Tables 1 and 2 show the primary attributes of the eligible studies from each target nation by SCS and CCCS. The variables presented were study type, disease domain, data source, outcomes, participant demographics, length of study duration, time gap from last data collected to publication, sample size, and number of research centers.

**Table 1:** Study characteristics in the solo scholar cluster across the 7 target countries by single-country study (SCS) and cross-country collaboration study (CCCS; n=369)^a^.

Study characteristics			Singapore (n=157)				Hong Kong (n=130)				Malaysia (n=100)		
			SCS (n=80)		CCCS (n=77)		SCS (n=86)		CCCS (n=44)		SCS (n=50)		CCCS (n=50)
**Study type, n (%)**													
	CER^b^	54 (68)		44 (57)		57 (66)		31 (70)		28 (56)		27 (54)	
	Non-CER (descriptive)	26 (32)		33 (43)		29 (34)		13 (30)		22 (44)		23 (46)	
**Disease area, n (%)**													
	CVM^c^	33 (41)		32 (42)		36 (42)		20 (45)		13 (26)		25 (50)	
	IDV^d^	7 (9)		3 (4)		18 (21)		3 (7)		7 (14)		7 (14)	
	IAD^e^	2 (2)		3 (4)		6 (7)		1 (2)		6 (12)		2 (4)	
	Oncology	11 (14)		9 (12)		8 (9)		13 (30)		10 (20)		7 (14)	
	Others	27 (34)		30 (39)		18 (21)		7 (16)		14 (28)		9 (18)	
**Database type, n (%)**													
	EMR^f^ or EHR^g^	43 (54)		19 (25)		69 (80)		12 (27)		11 (22)		12 (24)	
	Clinical registry	46 (58)		63 (82)		22 (26)		35 (80)		39 (78)		41 (82)	
	Health insurance and claims	4 (5)		1 (1)		0 (0)		1 (2)		0 (0)		0 (0)	
	Pharmacy claims	1 (1)		0 (0)		0 (0)		1 (2)		2 (4)		0 (0)	
	Multiple databases	12 (15)		5 (6)		5 (6)		3 (7)		2 (4)		3 (6)	
**Study outcome, n (%)**													
	Clinical	78 (98)		76 (99)		86 (100)		44 (100)		49 (98)		48 (96)	
	Cost	4 (5)		0 (0)		2 (2)		0 (0)		0 (0)		0 (0)	
	PROs^h^	0 (0)		5 (6)		1 (1)		2 (5)		1 (2)		7 (14)	
**Study population, n (%)**													
	Adults	63 (79)		61 (79)		67 (78)		37 (84)		32 (64)		37 (74)	
	Mixed	13 (16)		9 (12)		16 (19)		7 (16)		12 (24)		8 (16)	
	Pediatric	4 (5)		7 (9)		3 (3)		0 (0)		6 (12)		5 (10)	
Study duration (y), mean (SD)			6.6 (4.8)		7.8 (7.4)		9.8 (7.3)		7.9 (6.7)		7.1 (4.2)		5.5 (7.4)
**Lag period (y) from end of research to publication**													
	Overall, mean (SD)	5.8 (3.0)		4.8 (2.5)		5.1 (2.9)		5.0 (2.6)		4.8 (1.6)		4.6 (2.5)	
	<2, n (%)	3 (4)		7 (9)		11 (13)		4 (9)		1 (2)		7 (14)	
	2-5, n (%)	39 (49)		47 (61)		46 (53)		23 (52)		33 (66)		24 (48)	
	≥6, n (%)	34 (42)		16 (21)		26 (30)		10 (23)		16 (32)		9 (18)	
	Unknown, n (%)	4 (5)		7 (9)		3 (3)		7 (16)		0 (0)		10 (20)	
**Study size, mean (SD)**													
	Sample size (in thousands)	56.0 (180.9)		813.4 (4383.2)		205.0 (607.8)		4187.1 (23,979.1)		16.1 (30.7)		844.0 (4526.1)	
	Number of centers	4.4 (5.1)		89.7 (215.0)		42.7 (60.2)		66.8 (147.8)		19.5 (15.4)		92.4 (156.9)	

^a^Study numbers for database types and study outcomes may appear as duplicates; hence, the total percentage may not add up to 100. CCCS numbers may appear as duplicates for studies conducted in multiple target countries. The percentages may add up to less or more than 100 because of rounding.

^b^CER: comparative effectiveness research.

^c^CVM: cardiology and metabolic disorders.

^d^IDV: infectious diseases and vaccines.

^e^IAD: inflammatory and autoimmune disorders.

^f^EMR: electronic medical record.

^g^EHR: electronic health record.

^h^PRO: patient-reported outcome.

**Table 2:** Study characteristics in the global collaborator cluster across the 7 target countries by single-country study (SCS) and cross-country collaboration study (CCCS; n=369)^a^.

Study characteristics		Indonesia (n=33)		Pakistan (n=31)		Vietnam (n=27)		The Philippines (n=22)	
		SCS (n=7)	CCCS (n=26)	SCS (n=13)	CCCS (n=18)	SCS (n=7)	CCCS (n=20)	SCS (n=3)	CCCS (n=19)
**Study type, n (%)**									
	CER^b^	3 (43)	14 (54)	1 (8)	12 (67)	5 (71)	11 (55)	1 (33)	10 (53)
	Non-CER (descriptive)	4 (57)	12 (46)	12 (92)	6 (33)	2 (29)	9 (45)	2 (67)	9 (47)
**Disease area, n (%)**									
	CVM^c^	1 (14)	13 (50)	2 (15)	5 (28)	2 (29)	9 (45)	1 (33)	8 (42)
	IDV^d^	2 (29)	6 (23)	4 (31)	9 (50)	0 (0)	4 (20)	1 (33)	1 (5)
	IAD^e^	2 (29)	2 (8)	0 (0)	1 (6)	3 (43)	1 (5)	0 (0)	2 (11)
	Oncology	1 (14)	3 (12)	5 (38)	1 (6)	1 (14)	2 (10)	0 (0)	2 (11)
	Others	1 (14)	2 (8)	2 (15)	2 (11)	1 (14)	4 (20)	1 (33)	6 (32)
**Database type, n (%)**									
	EMR^f^ or EHR^g^	2 (29)	12 (46)	9 (69)	9 (50)	4 (57)	10 (50)	1 (33)	7 (37)
	Clinical registry	4 (57)	18 (69)	3 (23)	13 (72)	1 (14)	12 (60)	2 (67)	14 (74)
	Health insurance and claims	1 (14)	0 (0)	0 (0)	0 (0)	2 (29)	0 (0)	0 (0)	0 (0)
	Pharmacy claims	0 (0)	0 (0)	1 (8)	0 (0)	0 (0)	0 (0)	0 (0)	0 (0)
	Multiple databases	0 (0)	4 (15)	0 (0)	4 (22)	0 (0)	2 (10)	0 (0)	2 (11)
**Study outcome, n (%)**									
	Clinical	7 (100)	26 (100)	13 (100)	16 (89)	6 (86)	20 (100)	2 (67)	19 (100)
	Cost	2 (29)	0 (0)	0 (0)	0 (0)	2 (29)	0 (0)	1 (33)	0 (0)
	PROs^h^	0 (0)	3 (12)	0 (0)	3 (17)	0 (0)	0 (0)	0 (0)	3 (16)
**Study population, n (%)**									
	Adults	5 (71)	17 (65)	3 (23)	8 (44)	7 (100)	12 (60)	1 (33)	14 (74)
	Mixed	2 (29)	6 (23)	9 (69)	8 (44)	0 (0)	5 (25)	2 (67)	4 (21)
	Pediatric	0 (0)	3 (12)	1 (8)	2 (11)	0 (0)	3 (15)	0 (0)	1 (5)
Study duration (y), mean (SD)		3.1 (3.3)	7.1 (9.6)	2.1 (1.9)	9.1 (11.2)	4.8 (7.0)	8.2 (10.1)	12.0 (8.2)	8.4 (9.8)
**Lag period (y) from end of research to publication**									
	Overall, mean (SD)	4.3 (1.8)	4.2 (2.4)	3.8 (1.6)	3.3 (1.8)	4.6 (1.3)	4.7 (2.6)	4.0 (1.7)	3.9 (2.3)
	<2, n (%)	1 (14)	5 (19)	2 (15)	6 (33)	0 (0)	3 (15)	0 (0)	4 (21)
	2-5, n (%)	4 (57)	16 (62)	8 (62)	6 (33)	6 (86)	11 (55)	2 (67)	13 (68)
	≥6, n (%)	2 (29)	2 (8)	2 (15)	1 (6)	1 (14)	5 (25)	1 (33)	1 (5)
	Unknown, n (%)	0 (0)	3 (12)	1 (8)	5 (28)	0 (0)	1 (5)	0 (0)	1 (5)
**Study size, mean (SD)**									
	Sample size (in thousands)	122.6 (304.2)	1513.4 (6235.5)	5.9 (9.8)	2226.8 (7525.4)	202.7 (525.9)	1982.8 (7132.4)	161.9 (280.2)	2094.2 (7322.2)
	Number of centers	5.0 (3.6)	88.9 (92.7)	19.9 (13.4)	35.7 (48.3)	11.7 (4.9)	181.9 (236.9)	3.0 (—^i^)	81.6 (96.7)

^a^Study numbers for database types and study outcomes may appear as duplicates; hence, the total percentage may not add up to 100. CCCS numbers may appear as duplicates for studies conducted in multiple target countries. The percentages may add up to less or more than 100 because of rounding.

^b^CER: comparative effectiveness research.

^c^CVM: cardiology and metabolic disorders.

^d^IDV: infectious diseases and vaccines.

^e^IAD: inflammatory and autoimmune disorders.

^f^EMR: electronic medical record.

^g^EHR: electronic health record.

^h^PRO: patient-reported outcome.

^i^Not applicable.

#### Study Type: CER or Non-CER (Descriptive)

Of the 369 studies, 221 (59.9%) were CER studies, with the remaining 148 (40.1%) being non-CER or descriptive. The relative representation of CER versus non-CER for SCSs and CCCSs is illustrated in Multimedia Appendix 2. Singapore, Hong Kong, Malaysia, and Vietnam had a higher number of CER studies in both their SCSs and CCCSs. Vietnam’s SCSs had the predominant CER representation at 71% (5/7), followed by Singapore at 68% (54/80) and Hong Kong at 66% (57/86). Among CCCSs, Hong Kong led with 70% (31/44) CER studies, followed by Pakistan with 67% (12/18) and Singapore with 57% (44/77). There were more descriptive non-CER studies in SCSs from Pakistan, the Philippines, and Indonesia, resulting in the CER study percentages being 8% (1/13), 33% (1/3), and 43% (3/7), respectively.

**Figure 5:**
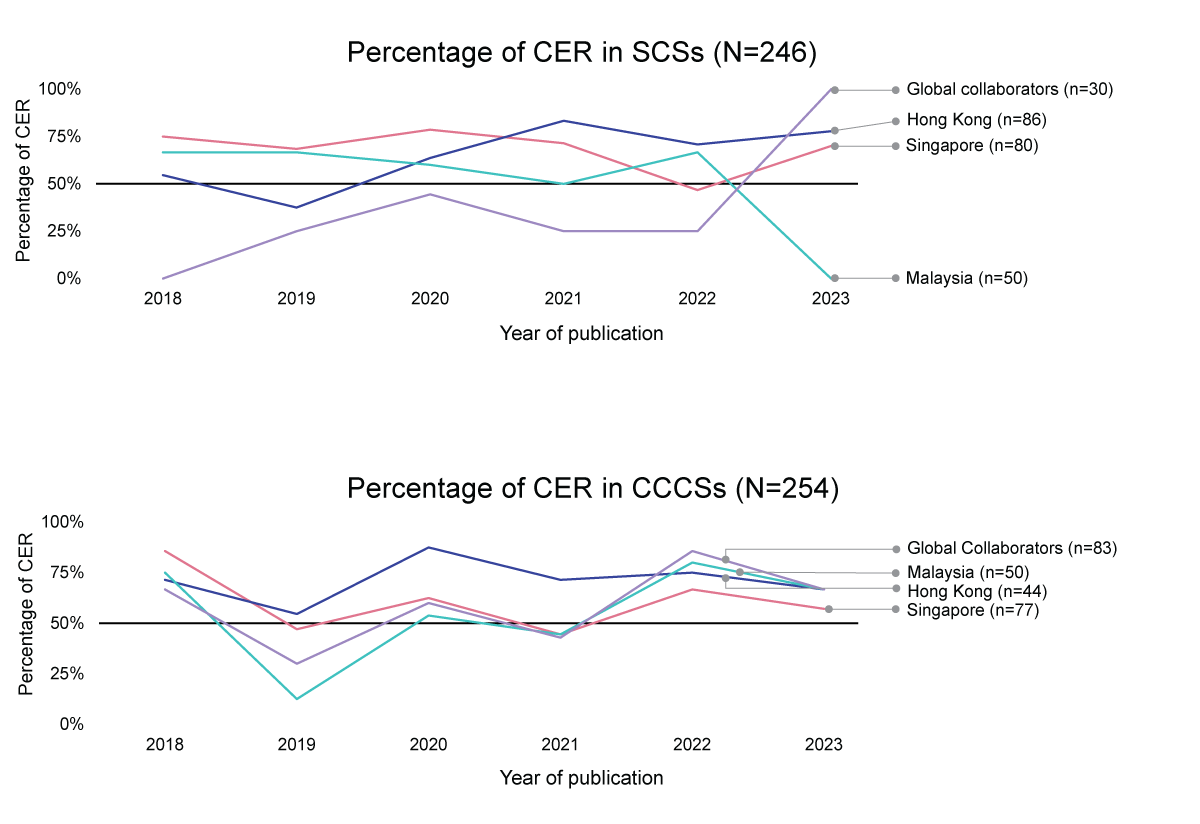
Trends in comparative effectiveness research (CER) by single-country studies (SCSs) and cross-country collaboration studies (CCCSs). Trends in CCCSs across the 7 target countries showed more consistency compared to trends in SCSs. The SCSs of Hong Kong and the global collaborators showed an upward trend in CER percentages, whereas Malaysia’s SCSs consistently decreased in CER contribution. The count of CCCSs represents the individual contributions of each country, leading to a total count across countries that exceeds the actual number of CCCSs due to some studies involving multiple collaborators.

Figure 5 shows the yearly trends of CER percentages from 2018 to 2023 broken down by SCS and CCCS. The consistency in trends was more noticeable in SCSs across the 7 target nations compared to CCCSs. An upward trend in CER study percentage was observed in SCSs from Hong Kong and the global collaborators. Conversely, Malaysia’s SCSs experienced a steady decrease in CER contribution over the same period.

The 2-year SMA trends for CER and descriptive studies are illustrated in Multimedia Appendix 2 for the biennial average from 2018 to 2023. Hong Kong consistently increased its CER contributions in both SCSs and CCCSs, increasing from 47% (9/19) between 2018 and 2019 to 73% (24/33) between 2022 and 2023 for SCSs and similarly from 61% (11/18) between 2018 and 2019 to 73% (8/11) between 2022 and 2023 for CCCSs. Other notable rises in CER contributions in CCCSs were observed in Malaysia (from 4/12, 33% between 2018 and 2019 to 12/16, 75% between 2022 to 2023), Indonesia (from 3/9, 33% between 2018 to 2019 to 7/10, 70% between 2022 and 2023), Pakistan (from 1/4, 25% between 2018 and 2019 to 8/9, 89% between 2022 and 2023), and Vietnam (from 4/9, 44% between 2019 and 2020 to 6/7, 86% between 2022 and 2023). Conversely, Malaysia’s SCSs saw a consistent decline in CER contribution over the 5 years, dropping from 67% (8/12) between 2018 and 2019 to 53% (10/19) between 2022 and 2023. Furthermore, all of Pakistan’s SCSs (12/12, 100%) were non-CER between 2018 and 2022.

#### Database Type

Of the 369 studies, 341 (92.4%) used a single database. Exclusive use of clinical registry databases was most common at 50.9% (188/369), followed by electronic medical records (EMRs) or electronic health records (EHRs) at 39.3% (145/369), health insurance and administrative claims at 1.4% (5/369), and pharmacy claims at 0.8% (3/369). The use of multiple databases was found in 7.6% (28/369) of the studies, primarily combining clinical registries and EMRs or EHRs (Multimedia Appendix 2). Use of EMR or EHR databases was more common for SCSs (120/246, 48.8%; Multimedia Appendix 2). On the other hand, the predominant exclusive database warehouse for CCCSs was clinical registries, used in 73.2% (9/123) of the studies. EMRs’ or EHRs’ contribution to CCCSs was lower, representing only 20.3% (25/123) of CCCSs, which is considerably lower than their share in SCSs (Tables 1 and 2 and Multimedia Appendix 2).

The use of the clinical registry database type consistently dominated across all CCCSs from all target nations, whether used on its own or in combination with other databases. For SCSs, (1) there were more clinical registries over EMRs or EHRs used in Indonesia, Malaysia, and the Philippines—the figures were 57% (4/7) versus 29% (2/7), 78% (39/50) versus 22% (11/50), and 67% (2/3) versus 33% (1/3), respectively; (2) Singapore’s use was almost even, with 58% (46/80) of the studies using clinical registries and 54% (43/80) using EMRs or EHRs; and (3) conversely, Hong Kong, Pakistan, and Vietnam used more EMRs or EHRs than clinical registries—80% (69/86) versus 26% (22/86), 69% (9/13) versus 23% (3/13), and 57% (4/7) versus 14% (1/7), respectively.

**Figure 6:**
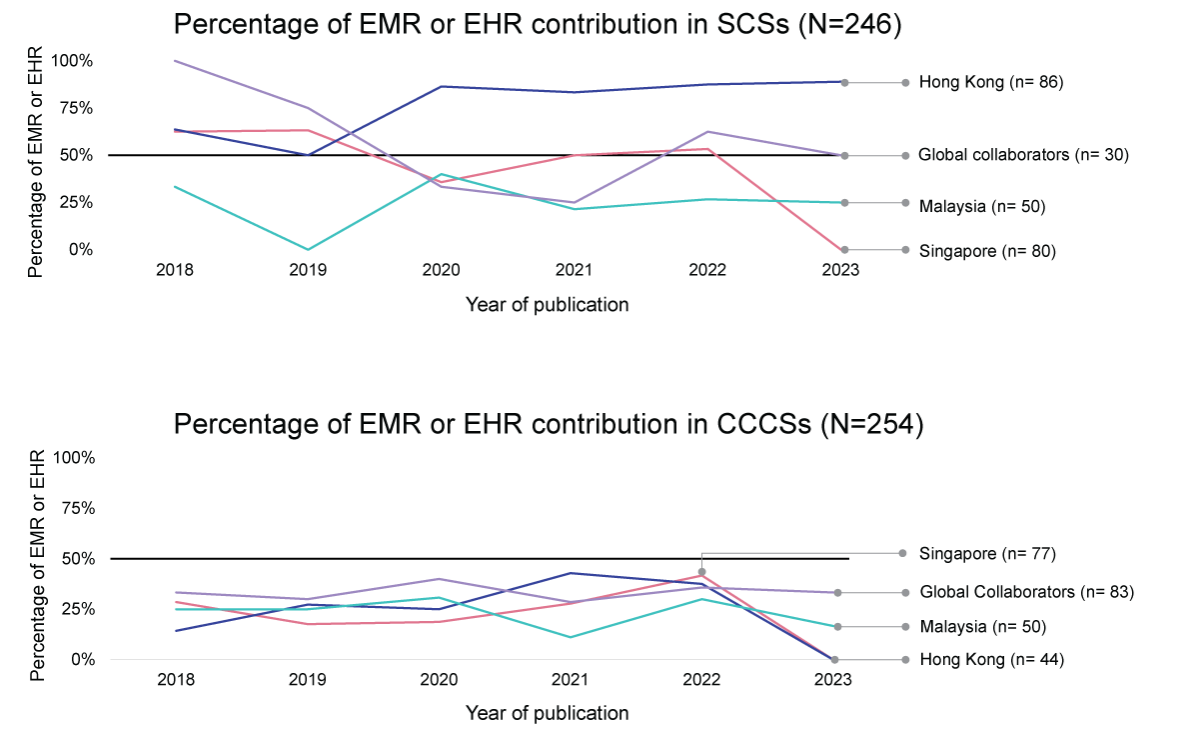
Trends in percentage of use of medical records by single-country studies (SCSs) and cross-country collaboration studies (CCCSs). A decline in exclusive or combined use of electronic medical records (EMRs) or electronic health records (EHRs) was observed for SCSs in Malaysia and the global collaborators, whereas Hong Kong saw an increase; in contrast, use of EMR or EHR databases in CCCSs was consistently below 50%, with no uniform trend emerging during this period. The count of CCCSs represents the individual contributions of each country, leading to a total count across countries that exceeds the actual number of CCCSs due to some studies involving multiple collaborators.

Figure 6 reveals the evolution of EMR or EHR contributions, both exclusively and in combination with other databases, in the previous 5 years. In SCSs, Malaysia and the global collaborators experienced a consistent decline in EMR or EHR use, whereas Hong Kong exhibited an increase. Malaysia’s EMR or EHR use in SCSs remained consistently at <50% during this period. In contrast to SCSs, where EMR or EHR use was predominant, EMR or EHR use in CCCSs from the target countries was always at <50% from 2018 to 2023, and no consistent time trend pattern was observed.

The 2-year SMA over the previous 5 years indicated that the exclusive use of EMRs or EHRs in SCSs from the 7 target nations increased from 46% (26/56) to 60% (49/82). In contrast, the reliance on clinical registry databases dipped from 50% (28/56) to 38% (31/82); Multimedia Appendix 2). For CCCSs, the distribution between clinical registries and EMRs or EHRs remained relatively steady, with clinical registries being the most common (Multimedia Appendix 2).

#### Disease Area

The leading medical research area was CVM, accounting for 36.9% (136/369) of the studies, trailed by oncology and IDV, each with 14.9% (55/369). Inflammatory and autoimmune disorders was the least prevalent area, representing 6.2% (23/369) of the studies. The remaining 27.1% (100/369) of the studies pertained to various other diseases. The proportion of CVM studies grew from 28% (11/39) in 2018 to 39% (14/36) in 2023, peaking in 2020 with 49% (36/73). Conversely, the share contributed by IDV medical area increased from 8% (6/73) in 2020 to 25% (21/84) in 2022 and 17% (6/36) in 2023, surpassing oncology as the second most common disease and therapeutics research area in recent years (Multimedia Appendix 2).

#### Study Outcomes

Most of the studies (348/369, 94.3%) presented clinical outcomes whether in terms of treatment effect or safety. There were 5.7% (21/369) of the studies that discussed cost outcomes, PROs, or a combination of these with clinical results. In the SCS category, every study from Pakistan focused on clinical outcomes, whereas cost outcomes were observed in SCSs from all countries except Malaysia and Pakistan. One study each from Hong Kong (1/86, 1%) and Malaysia (1/50, 2%) included PRO outcomes. In the CCCS category, none of the selected nations published studies focusing on cost outcomes (Tables 1 and 2).

#### Study Population

Of the 369 studies obtained, 273 (74%) investigated adults, 24 (6.5%) focused on the pediatric age group, and 72 (19.5%) encompassed both adult and pediatric participants. Notably, in the SCS category (Table 2), Pakistan (9/13, 69%) and the Philippines (2/3, 67%) reported higher proportions of mixed populations than of solely adult participants (Pakistan: 3/13, 23%; the Philippines: 1/3, 33%).

Pediatric representation in the CCCSs was 8.1% (10/123), slightly higher than in SCSs (14/246, 5.7%). Within CCCSs (Table 2), Vietnam led in pediatric-focused research with 15% (3/20) of the studies, followed by Indonesia (3/26, 12%) and Pakistan (2/18, 11%).

#### Study Duration

Information about study duration was reported for 94.6% (349/369) of the studies. The average duration was 7.4 (SD 6.3) years, ranging from 0.01 to 35.8 years. In total, 3.5% (13/369) of the studies had a duration of >20 years. The mean for SCSs was higher at 7.5 (SD 6.0) years compared to that for CCCSs at 7.1 (SD 6.8) years (Multimedia Appendix 2).

Among SCSs, the Philippines (Table 2) topped the list with the longest average study duration of 12.0 (SD 8.2) years. As shown in Table 1, Hong Kong followed closely with an average of 9.8 (SD 7.3) years. Conversely, Pakistan and Indonesia registered the shortest mean study durations with 2.1 (SD 1.9) years and 3.1 (SD 3.3) years, respectively. Over a 5-year span, based on a 2-year rolling average, the study duration in Indonesia showed an uptick, increasing from 0.6 years between 2018 and 2019 to 1.9 years between 2022 and 2023. Other target countries did not exhibit any consistent study duration trend patterns (Multimedia Appendix 2).

For CCCSs, Pakistan (Table 2) led with the longest mean study duration of 9.1 (SD 11.2) years, closely followed by the Philippines with 8.4 (SD 9.8) years. Malaysia (Table 1) recorded the shortest average study duration at 5.5 (SD 7.4) years. The study duration in Indonesia’s CCCSs averaged 7.1 (SD 9.6) years, which was notably longer than that of its SCSs. Observing trends, there was a decline in the mean study duration of CCCSs in Malaysia, Pakistan, the Philippines, and Vietnam. Conversely, the average study duration in Singapore’s CCCSs steadily rose, increasing from 5.6 years between 2018 and 2019 to 9.1 years between 2022 and 2023 (Multimedia Appendix 2).

#### Lag Between the Research Period and Publication

Of the eligible studies, most (205/369, 55.6%) were published between 2 and 5 years after the time of latest available data studied, 28.5% (105/369) were published after >6 years, and 9.5% (35/369) were published within 2 years. The remaining 6.5% (24/369) of the studies had unspecified year or years of research completion (Multimedia Appendix 2).

Eligible studies from all the target nations showed a similar trend, with most (205/345, 59.4%) being published within 2 to 5 years after the research period. However, in both Singapore and Hong Kong, the time taken from research completion to publication was notably longer for both SCSs and CCCSs, averaging 5.8 (SD 3.0) years and 5.1 (SD 2.9) years for SCSs and 4.8 (SD 2.5) years and 5.0 (SD 2.6) years for CCCSs, respectively (Table 1).

It is worth noting that 15.9% (17/107) of CCCSs were published within 2 years of the research period but only 7.6% (18/238) of SCSs were published within this time frame. We observed upward trends in the time to publication within 2 years from 2018 to 2023. For SCSs, it was from 3% (2/63) to 15% (12/83), and for CCCSs, it was from 16% (6/38) to 20% (5/25; Multimedia Appendix 2).

The publication time lag also varied according to the RWD source (Multimedia Appendix 2). Among studies that relied on a single database, the highest percentage of those published within 2 years after the research consistently used the EMR or EHR database type. This trend held true for both SCSs and CCCSs, with EMRs or EHRs dominating the quick turnaround for publications. Specifically, among SCSs, 12.8% (15/117) of studies using the EMR or EHR database type were published within this 2-year time frame, whereas only 2% (2/94) of those using clinical registry databases had the same publishing speed. CCCSs had a consistent pattern—26% (6/23) of the studies that used EMRs or EHRs were published within 2 years, in contrast to the 14% (11/78) of the studies that used clinical registry databases. Notably, no studies published within the 2-year window used the health insurance and medical claims database type or the pharmacy claims database type.

#### Study Size: Sample Size and Number of Centers

The sample size was specified in 98.1% (362/369) of the studies and varied considerably, ranging from as few as 16 to >154,500,000. The average sample size was 672,352 (SD 8,364,280). The average in CCCSs was much higher at 1,824,035 (SD 14,530,091) compared to 106,029 (SD 397,050) in SCSs (Multimedia Appendix 2). The 2-year SMA of sample size in SCSs indicated an increasing trend from 51,706 to 178,675 from 2018 to 2023. In contrast, a sharp decline was observed in CCCS sample sizes from 5,543,271 to 548,107 during the same period (Multimedia Appendix 2). Hong Kong had the highest average sample size in SCSs (205,006, SD 607,767) as well as in CCCSs (4,187,122, SD 23,979,119; Tables 1 and 2).

The number of participating centers was only specified in 44.4% (164/369) of the studies. The mean number of centers was 44.3 (SD 120.3), ranging from 2 to 1119. As noted in Tables 1 and 2, Hong Kong reported the highest average number of study centers at 42.7 (SD 60.2) for SCSs, followed by Pakistan at 19.9 (SD 13.4) and Malaysia at 19.5 (SD 15.4). For CCCSs, Vietnam had the highest mean number of study centers at 181.9 (SD 236.9), which was higher than twice the overall CCCS mean of 78.4 (SD 178.6).

The 2-year SMA for the number of study centers in SCSs initially rose from 81.8 between 2018 and 2019, reaching a peak of 131.9 between 2019 and 2020 before declining to 99.1; 37.1; and, finally, 30.8 in the subsequent years. In CCCSs, there was a downtrend starting from 16.1 between 2018 and 2019 to 10.7 centers at its lowest point between 2020 and 2021 before bouncing back to 33.9 centers between 2022 and 2023 (Multimedia Appendix 2).

Table S5 in Multimedia Appendix 1 provides and overview of the number of centers involved in RWD studies across various database types in the target countries. The Philippines reported the highest average number of centers (197.0) in studies using EMR or EHR databases, whereas Vietnam had the highest average (65.8) in studies associated with clinical registry databases. Information on the number of centers was not reported in many studies, particularly those using health insurance and claims databases.

### Database Names

Multimedia Appendix 3 provides the specific name or names of the databases used in each study organized by target country, database type, and disease area.

## Discussion

### Principal Findings

This scoping review was based on our earlier research completed for Taiwan, India, and Thailand in Asia [7]. We have now expanded insights on integrated real-world study databases across 7 additional diverse Asian health care systems in Hong Kong, Indonesia, Malaysia, Pakistan, the Philippines, Singapore, and Vietnam. This has enabled us to provide a comprehensive perspective on the current landscape of RWE generation in these nations, thus aiding stakeholders in formulating informed research and policy decisions [22,23].

The archetyping of the target nations into 2 country clusters (ie, solo scholars and global collaborators) allowed us to uncover distinct patterns reflective of differing resources, priorities, and strategic objectives. Solo scholars tend to conduct independent studies, which exemplifies that these nations are equipped with robust research infrastructures. This autonomy allows for a deep dive into national health issues tailored to specific local contexts and country priorities [24]. On the other hand, global collaborators frequently engaged in international partnerships, a strategy that would be likely born out of necessity due to limited research funding and resources within the nation and, hence, a greater reliance on collaborative networks [25]. We found that >90% of CCCSs from global collaborators involving nontarget countries (76/83, 92%) also partnered with some of the 7 target nations. This collaborative pattern possibly indicated a stronger network of research collaboration within the neighboring regions of Asia. In contrast, among solo scholars such as Singapore and Hong Kong, despite the high number of CCCSs involving nontarget countries (>94%; 73/77, 95% in Singapore and 43/44, 98% in Hong Kong), there were approximately 40% of studies (33/77, 43%; 17/44, 39%) not partnered with other target nations. However, the emphasis on global or regional priorities could overshadow the unique health challenges and priorities of these nations. This imbalance can lead to a scarcity of data and insights directly applicable to domestic health care [26].

Among solo scholars, Hong Kong, Malaysia, and Singapore emerged as significant contributors to domestic RWD publications, showcasing their robust research infrastructure and commitment to harnessing RWD. In contrast, global collaborators, and especially the Philippines, had fewer studies, which could be attributed to various reasons, from funding sources to bureaucracies in research grant administration [27,28]. Singapore emerged as the predominant contributor to the CCCS pool, but on average, Malaysia collaborated with more countries. It is also particularly noteworthy that, from 2018 to 2023, most of our target nations (except the Philippines) manifested an increasing trend in the average number of studies published annually, with Vietnam leading in growth. Vietnam’s health care system has been consistently advancing, and the nation is enhancing its research capabilities; this progress has been widely acknowledged in the literature [23,29].

Diving deeper into the nature of these studies, there was an evident leaning toward CER over descriptive studies in several nations, such as Vietnam, Singapore, and Hong Kong. The proportion of CER to non-CER studies offers insights into the nature of the questions that researchers in different regions were keen to address. Regions with a higher proportion of CER studies, such as Vietnam’s SCSs, suggest an active interest in comparing the outcomes of different interventions and preventive and prognostic strategies, which can be crucial for policy decisions, including effective containment of COVID-19. Vietnam, along with Singapore and Hong Kong, has been extensively praised for effectively controlling the spread of COVID-19, especially during the early stages of the pandemic [30,31]. While we cannot assert a direct link with certainty, the possibility of a connection through RWD from CER also playing a role in fostering better-informed public health decisions cannot be denied [32]. Moreover, Singapore and Hong Kong’s well-established reputation as quality research hubs in Asia could further underscore the potential impact of robust research frameworks [33,34].

The predilection for certain database types, be it clinical registries or EMRs or EHRs, was due to a combination of availability and convenience and, hence, the ease of use of these databases in different regions. The 5-year trend showcased the evolving dynamics in RWD research. Nations leaning toward EMR or EHR databases, such as Hong Kong, might be signaling greater digitization of their health records or the perceived value in this data type [35,36], whereas nations such as Indonesia, Malaysia, and the Philippines primarily leveraged clinical registries. Apart from Hong Kong, Pakistan and Vietnam also displayed a marked inclination toward EMR or EHR use.

The variance in research duration offers a window into research efficiency and the possible administrative or infrastructural bottlenecks. Longer durations in nations such as the Philippines could indicate complex, long-term studies or challenges in study execution and continuity [27]. In addition, the expectation that RWD accelerates evidence generation is not reflected in our findings, where there was an average lag of approximately 2 to 5 years from research completion to publication. Notably, around 90% of the studies (310/345, 89.9%) extended beyond 2 years to reach publication, suggesting room for enhancing the efficiency of evidence generation, potentially through targeted support mechanisms. Interestingly, a comparatively higher proportion of CCCSs in contrast to SCSs (17/107, 15.9% vs 18/238, 7.6%) were published within 2 years, hinting at the efficiency benefits that international partnerships might offer in expediting research outputs. Larger study sample sizes and a greater number of centers, as observed in Hong Kong, reflect the ability to conduct expansive studies from territory-wide linked databases, indicating a propensity for large-scale, nationally representative research [37]. Similarly, the number of centers involved could also indicate the collaborative spirit within the research community or the need to pool resources.

The strategic application of RWE in health care research and policy formation is clearly evident on a global scale. Although there is an increasing reliance on HTA as a pivotal tool for informed health care decision-making, the nations categorized largely under the global collaborators cluster face a tremendous challenge in health care infrastructure and economic constraints to lead independent RWE that could shape local health care policy and reimbursement decision-making [38,39]. Hence, open data initiatives and international collaborations such as the Observational Health Data Sciences and Informatics and the HTAsiaLink, respectively, are crucial in this regard [40,41]. There is also the potential for other international networks (eg, the International Network of Agencies for Health Technology Assessment) in facilitating the alignment of health care policies with benchmark evidence-based practices [42].

### Limitations

In our efforts to comprehensively assess the landscape of research in the field, we encountered challenges in data extraction, mainly due to inconsistencies in how various studies reported certain characteristics such as the number of centers or study periods. For instance, while some studies provided clear details on their duration, others only specified the enrollment phase, leaving us to speculate on the follow-up or observation period. In addition, the screening and extraction process involved multiple reviewers working under tight schedules. We acknowledge that this approach diverges from the ideal practice of having at least 2 independent investigators screen each title, abstract, and full text and subsequently extract data blinded to each other’s decisions. While we implemented quality checks, including spot assessments and team discussions, the constraints may have inevitably introduced occasional inaccuracies.

Moreover, in some nations, the limited number of studies could make percentage-based analyses less reflective of the true study landscape. Still, these analyses offer a preliminary understanding of research trends in those regions. We must also acknowledge that our analysis, with a search conducted in May 2023, assumes a linear distribution for studies in 2019 and 2023, which might slightly deviate from the actual figures due to approximations.

We might have inadvertently overlooked some relevant studies, especially if abstracts failed to mention key terms such as RWD or RWE. Our decision to focus on English-language publications and rely solely on PubMed for citations, while strategic, may have missed a handful of non–English-language studies or those in other databases. Nonetheless, the vast number of studies that we analyzed offers valuable insights into using RWD to produce RWE in our target countries. In addition, we did not report study design and funding sources for the included studies. While this information may have been valuable, we faced challenges in the extraction of study design and funding information due to lack of clear and consistent reporting across publications. This necessitated the exclusion of these variables to prevent any subjective interpretations.

Despite these challenges, our findings underscore a need in the research community—a call for clearer, more standardized reporting on the databases used, study design, analysis methods, and important time points. A particular area that warrants attention is the clarity in detailing study duration (which should encompass the recruitment and observation periods), study design, and funding sources. While we recognize this study’s limitations, we believe that it also paves the way for refining research methodologies in the future.

### Conclusions: The Path Forward

Our comprehensive assessment of studies across the selected nations reveals intricate patterns that explain the diverse research landscape for RWD generation. Each nation’s unique landscape for contemporary integrated RWD warehouses tells a narrative that is partially attributed to their economic, clinical, and research settings. Delving deeper into these patterns aids in formulating robust insights for future endeavors in health care research and policy making, including prioritization of competency building based on a nation’s unique infrastructure, skill sets, and research strengths and weaknesses [4]. As the health care landscape evolves, there is an undeniable value in understanding and leveraging RWD [43]. Recognizing the diverse approaches and challenges across countries can lead to more collaborative and informed strategies [4]. The goal should be to address the present gaps and pave the way for future synergistic, impactful, and patient-centric research [3].

In conclusion, the observed variations across nations reiterate the essence of context in health care research. Every nation’s unique story, as told by their data, accentuates the need for a tailored approach in using RWD—ensuring that they truly serve the multifaceted needs of health care research and decision-making.

## Appendix

Multimedia Appendix 1 Supplementary tables.

Multimedia Appendix 2 Supplementary figures.

Multimedia Appendix 3 Identified databases.

## Abbreviations

ANC: average number of collaborative countries
CCCS: cross-country collaboration study
CER: comparative effectiveness research
CVM: cardiology and metabolic disorders
EHR: electronic health record
EMR: electronic medical record
HTA: health technology assessment
IDV: infectious diseases and vaccines
PRISMA-ScR: Preferred Reporting Items for Systematic Reviews and Meta-Analyses extension for Scoping Reviews
PRO: patient-reported outcome
RWD: real-world data
RWE: real-world evidence
SCS: single-country study
SMA: simple moving average

## Data Availability

Data generated or analyzed during this study are included in Multimedia Appendices 1-3.
