# Supplementary material for: Integrated Real-World Data Warehouses Across 7 Evolving Asian Health Care Systems: Scoping Review
Source: J Med Internet Res. 2024 Jun 11;26:e56686. doi: 10.2196/56686 (PMC11200047; doi:10.2196/56686)
Supplement: Multimedia Appendix 1 [file jmir_v26i1e56686_app1.pdf]

Table S1. Number of citations retrieved from PubMed based on search strategy

#	Query	Results
**1**	Concept 1<br>(“Treatment Outcome”[MeSH] OR “Evidence-Based Medicine”[MeSH] OR “Time Factors”[MeSH] OR “Retrospective Studies”[MeSH] OR “real world” OR “real-world” OR “RWD” OR “RWE” OR “real life” OR “real patient” OR “real practice” OR “real clinical” OR “real population” OR “actual life” OR “actual world” OR “actual patient” OR “actual clinical” OR “actual practice” OR “actual population”)	3,253,219
**2**	Concept 2<br>(“Hong Kong”[MeSH] OR “Hong Kong” OR “Indonesia”[MeSH] OR “Indonesia” OR “Malaysia” [MeSH] OR “Malaysia” OR “Pakistan”[MeSH] OR “Pakistan” OR “Philippines”[MeSH] OR “Philippines” OR “Singapore” [MeSH] OR “Singapore” OR “Vietnam” [MeSH] OR “Vietnam”)	544,737
**3**	Concept 3<br>(“Electronic Health Records”[MeSH] OR “Insurance, Health”[MeSH] OR “Registries”[MeSH] OR “Pharmaceutical Services”[MeSH] OR “Databases, Pharmaceutical”[MeSH] OR “registry” OR “registries” OR “electronic healthcare record*” OR “electronic health record*” OR “electronic medical record*” OR “EHRs” OR “EHR” OR “EMRs” OR “EMR” OR “claims database*” OR “administrative database*” OR “hospital data” OR “claims data” OR “electronic health data” OR “electronic healthcare data” OR “informatics” OR “clinical database*”)	704,755
**4**	#1 AND #2 AND #3[a]	**2,759**
**5**	#4 with filter for English[Language]	**2,757**
**6**	**#5 with filter for published in last 5 years[b]**	**1,483**

[a]((("Treatment Outcome"[MeSH] OR "Evidence-Based Medicine"[MeSH] OR "Retrospective Studies"[MeSH] OR "Time Factors"[MeSH] OR "real world" OR "real-world" OR "RWD" OR "RWE" OR "real life" OR "real patient" OR "real practice" OR "real clinical" OR "real population" OR "actual world" OR "actual life" OR "actual patient" OR "actual practice" OR "actual clinical" OR "actual population")) AND (("Hong Kong"[MeSH] OR "Hong Kong" OR "Indonesia"[MeSH] OR "Indonesia" OR "Malaysia" [MeSH] OR "Malaysia" OR "Pakistan"[MeSH] OR "Pakistan" OR "Philippines"[MeSH] OR "Philippines" OR "Singapore" [MeSH] OR "Singapore" OR "Vietnam" [MeSH] OR "Vietnam"))) AND (("Electronic Health Records"[MeSH] OR "Insurance, Health"[MeSH] OR "Registries"[MeSH] OR "Databases, Pharmaceutical"[MeSH] OR "Pharmaceutical Services"[MeSH] OR "registry" OR "registries" OR " electronic health record*" OR "electronic healthcare record*" OR "electronic medical record*" OR "EHR" OR "EHRs" OR "EMR" OR "EMRs" OR "claims database*" OR "administrative database*" OR "hospital data" OR "claims data" OR "electronic health data" OR "clinical database*" OR "electronic healthcare data" OR "informatics"))

Table S2. Eligibility criteria for data extraction

Inclusion criteria	Exclusion criteria
**Database types**	
• Studies involving medical records (EMR/ EHR[a]), health insurance claims, clinical registries, and/or pharmacy databases<br>AND<br>• Databases with research data involving more than 1 hospital/clinic	• A research data source involving medical records, health insurance claims, clinical registries, or pharmacy databases is not mentioned<br>OR<br>• Databases with research data involving only one hospital/clinic
**Publication types**	
• Original research (including brief reports/ research letters/ short communications/)	• Editorials/ commentaries<br>• Correspondence/ letters to the editor<br>• Case reports, case series (except retrospective case series with real-world study design)<br>• Guidelines<br>• Narrative, systematic, or scoping reviews
**Study types**	
All types of real-world studies or their protocols using the following databases:<br>• EHR/ EMRs<br>• Health insurance claims databases<br>• Clinical registries<br>• Pharmacy claims databases	The following **types of studies**:<br>• Pragmatic clinical trials<br>• Randomized clinical trials<br>• Non-human studies<br>• Pre-clinical studies
**Scope of publication**	
Studies with databases involving Hong Kong, Indonesia, Malaysia, Pakistan, Philippines, Singapore, or Vietnam<br><br>Eligible CCCS studies will be included, provided any of the above-mentioned target countries are included	Studies with scope outside Hong Kong, Indonesia, Malaysia, Pakistan, Philippines, Singapore, or Vietnam

[a]EHR: electronic health record/ EMR: electronic medical record.

[b]CCCS: cross-country collaboration studies.

Table S3. Number and distribution of all eligible SCS and CCCS from linked research databases in target countries

Country	SCS[a] from individual target countries (n=246), n (%)	CCCS[b] between target/ nontarget countries, including duplicates (n=254[c])											Total studies (SCS+CCCS; N=500[d]), n (%)
		Other countries (non-target; n=63), n (%)	Non-target countries and Hongkong (n=8), n (%)	Non-target countries and Indonesia (n=0), n (%)	Non-target countries and Malaysia (n=9), n (%)	Non-target countries and Pakistan (n=3), n (%)	Non-target countries and Philippines (n=2), n (%)	Non-target countries and Singapore (n=17), n (%)	Non-target countries and Vietnam (n=1), n (%)	Non-Target countries and two or more target countries (n=144), n (%)	All countries (Hong Kong, Indonesia, Malaysia, Pakistan, Philippines, Singapore, Vietnam; n=7), n (%)	Total CCCS by country, n (%)	
Hong Kong	86 (35.0)	17 (27.0)	NA[e]	0	1 (11.1)	0 (0.0)	0 (0.0)	7 (41.2)	0 (0.0)	18 (12.5)	1 (14.3)	44 (33.8)	130 (26.0)
Indonesia	7 (2.8)	1 (1.6)	0 (0.0)	NA	0 (0.0)	0 (0.0)	0 (0.0)	0 (0.0)	0 (0.0)	24 (16.7)	1 (14.3)	26 (78.8)	33 (6.6)
Malaysia	50 (20.3)	8 (12.7)	1 (12.5)	0	NA	2 (66.7)	0 (0.0)	6 (35.3)	0 (0.0)	32 (22.2)	1 (14.3)	50 (50.0)	100 (20.0)
Pakistan	13 (5.3)	3 (4.8)	0 (0.0)	0	2 (22.2)	NA	0 (0.0)	1 (5.9)	0 (0.0)	11 (7.6)	1 (14.3)	18 (58.1)	31 (6.2)
Philippines	3 (1.2)	0 (0.0)	0 (0.0)	0	0 (0.0)	0 (0.0)	NA	2 (11.8)	0 (0.0)	16 (11.1)	1 (14.3)	19 (86.4)	22 (4.4)
Singapore	80 (32.5)	33 (52.4)	7 (87.5)	0	6 (66.7)	1 (33.3)	2 (100.0)	NA	1 (100.0)	26 (18.1)	1 (14.3)	77 (49.0)	157 (31.4)
Vietnam	7 (2.8)	1 (1.6)	0 (0.0)	0	0 (0.0)	0 (0.0)	0 (0.0)	1 (5.9)	NA	17 (11.8)	1 (14.3)	20 (74.1)	27 (5.4)

[a]SCS: single-country studies.
[b]CCCS: cross-country collaboration studies.
[c]Duplications in CCCS adjusted for the number of studies from 254 to 123.
[d]Duplications in total studies adjusted for the number of studies from 500 to 369.
[e]N/A: not applicable.

Table S4. Cross-country collaboration network for seven target countries, n=369[a]

Target country	Cross-country collaboration studies n (%)	Average no. in collaboration[b]	n (% of cluster's/ country's cross-country collaboration studies) & SG n (%)[c]	& HK n (%)[c]	& MY n (%)[c]	& IN n (%)[c]	&PK n (%)[c]	& VN n (%)[c]	& PH n (%)[c]	& NTC n (%)[c]	Without other target countries[d]
Solo Scholars	171 (100)		51 (29.8)	37 (21.6)	44 (25.7)	48 (28.1)	21 (12.3)	33 (19.3)	38 (22.2)	164 (95.9)	58 (33.9)
Singapore	77 (100)	2.2	0 (0)	22 (28.6)	29 (37.7)	16 (20.8)	7 (9.1)	11 (14.3)	15 (19.5)	73 (94.8)	33 (42.9)
Hong Kong	44 (100)	2.5	22 (50.0)	0 (0)	15 (34.1)	11 (25.0)	4 (9.1)	7 (15.9)	7 (15.9)	43 (97.7)	17 (38.6)
Malaysia	50 (100)	3.1	29 (58.0)	15 (30.0)	0 (0)	21 (42.0)	10 (20.0)	15 (30.0)	16 (32.0)	48 (96.0)	8 (16.0)
Global Collaborators	83 (100)		49 (59.0)	29 (34.9)	62 (74.7)	31 (37.3)	18 (21.7)	22 (26.5)	23 (27.7)	76 (91.6)	5 (6.0)
Indonesia	26 (100)	4.0	16 (61.5)	11 (42.3)	21 (80.8)	0 (0)	8 (30.8)	10 (38.5)	13 (50.0)	24 (92.3)	1 (3.8)
Pakistan	18 (100)	3.1	7 (38.9)	4 (22.2)	10 (55.6)	8 (44.4)	0 (0)	6 (33.3)	4 (22.2)	17 (94.4)	3 (16.7)
Vietnam	20 (100)	3.7	11 (55.0)	7 (35.0)	15 (75.0)	10 (50.0)	6 (30.0)	0 (0)	6 (30.0)	19 (95.0)	1 (5.0)
Philippines	19 (100)	4.1	15 (78.9)	7 (36.8)	16 (84.2)	13 (68.4)	4 (21.1)	6 (31.6)	0 (0)	16 (84.2)	0 (0)

[a]Duplications in cross-country collaboration studies adjusted for the number of studies from 254 to 123.

[b]The 'average number in collaboration' is calculated by dividing the total instances of a country's collaborations by the number of cross-country collaborative studies it participates with other target and non-target countries. It shows how interconnected the research efforts are between the target countries.

[c]& SG, & HK, & MY, & IN, & PK, & VN, & PH, & NTC: These columns represent the specific number and percentage of studies in which the target country has collaborated with Singapore (SG), Hong Kong (HK), Malaysia (MY), India (IN), Pakistan (PK), Vietnam (VN), the Philippines (PH), and non-target countries (NTC), respectively.

[d]Without other target countries: This shows the number and percentage of studies that did not involve collaboration with any of the target countries included in this study.

Table S5. Overview of numbers of centres from each database type in target countries, n=369[a]

Target Country	Number of studies[l] N	Number of studies[l] n	EMR/EHR	Clinical Registry	Pharmacy Claims	Health Insurance/ Claims[k]	Multiple Databases
			Database Type: Mean (SD)				
Solo Scholars[b]	387	197	45.0 (112.8)	44.9 (133.6)	27.0 (35.4)	NA	10.7 (12.9)
Singapore[c]	157	70	37.2 (144.2)	39.0 (54.1)	NA	NA	6.7 (9.6)
Hong Kong[d]	130	55	66.3 (144.5)	59.1 (174.7)	NA	NA	20.7 (18.5)
Malaysia[e]	100	51	134.0 (239.1)	37.0 (39.8)	27.0 (35.4)	NA	9.0 (NA)
Global Collaborators[f]	113	61	99.9 (190.2)	42.6 (48.6)	4.0 (NA)	NA	NA
Indonesia[g]	33	18	149.2 (142.3)	46.3 (39.4)	NA	NA	NA
Pakistan[h]	31	18	38.4 (43.0)	17.6 (13.9)	4.0 (NA)	NA	NA
Vietnam[i]	27	49	176.8 (251.6)	65.8 (102.3)	NA	NA	NA
Philippines[j]	22	12	197.0 (227.7)	50.7 (35.9)	NA	NA	NA

[a]Duplications in cross-country collaboration studies adjusted for the number of studies from 254 to 123.

[b]Out of 387 studies from Solo Scholars, 197 studies provided the number of centres, while this information was not provided in the remaining 190 studies.

[c]Out of 157 studies from Singapore, 70 studies provided the number of centers, while this information was not provided in the remaining 87 studies

[d]Out of 130 studies from Hong Kong, 55 studies provided the number of centers, while this information was not provided in the remaining 75 studies

[e]Out of 100 studies from Malaysia, 51 studies provided the number of centers, while this information was not provided in the remaining 49 studies

[f]Out of 113 studies from Global Collaborators, 61 studies provided the number of centers, while this information was not provided in the remaining 52 studies

[g]Out of 33 studies from Indonesia, 18 studies provided the number of centers, while this information was not provided in the remaining 15 studies

[h]Out of 31 studies from Pakistan, 18 studies provided the number of centers, while this information was not provided in the remaining 13 studies

[i]Out of 27 studies from Vietnam, 13 studies provided the number of centers, while this information was not provided in the remaining 14 studies

[j]Out of 22 studies from the Philippines, 12 studies provided the number of centers, while this information was not provided in the remaining 10 studies

[k]All studies with exclusive use of health insurance/claims database(s) did not provide information on the number of centers.

[l]N is number of studies included from respective countries for data analysis while n is the number of studies from target countries with number of centers information available. n is used as a denominator to calculate mean.

[m]N/A: not applicable.
