# Supplementary material for: Integrated Real-World Data Warehouses Across 7 Evolving Asian Health Care Systems: Scoping Review
Source: J Med Internet Res. 2024 Jun 11;26:e56686. doi: 10.2196/56686 (PMC11200047; doi:10.2196/56686)
Supplement: Multimedia Appendix 2 [file jmir_v26i1e56686_app2.pdf]

Multimedia Appendix 2

Scoping review to identify and describe integrated contemporary real-world studies databases from three diverse healthcare systems in Asia: Hong Kong, Indonesia, Malaysia, Pakistan, Philippines, Singapore and Vietnam (target countries)

International Registered Report Identifier (IRRID): RR2-10.2196/43741

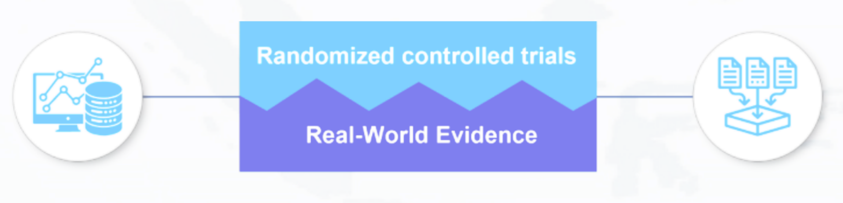

Supplementary Figures

Country-wise RWD studies from integrated databases

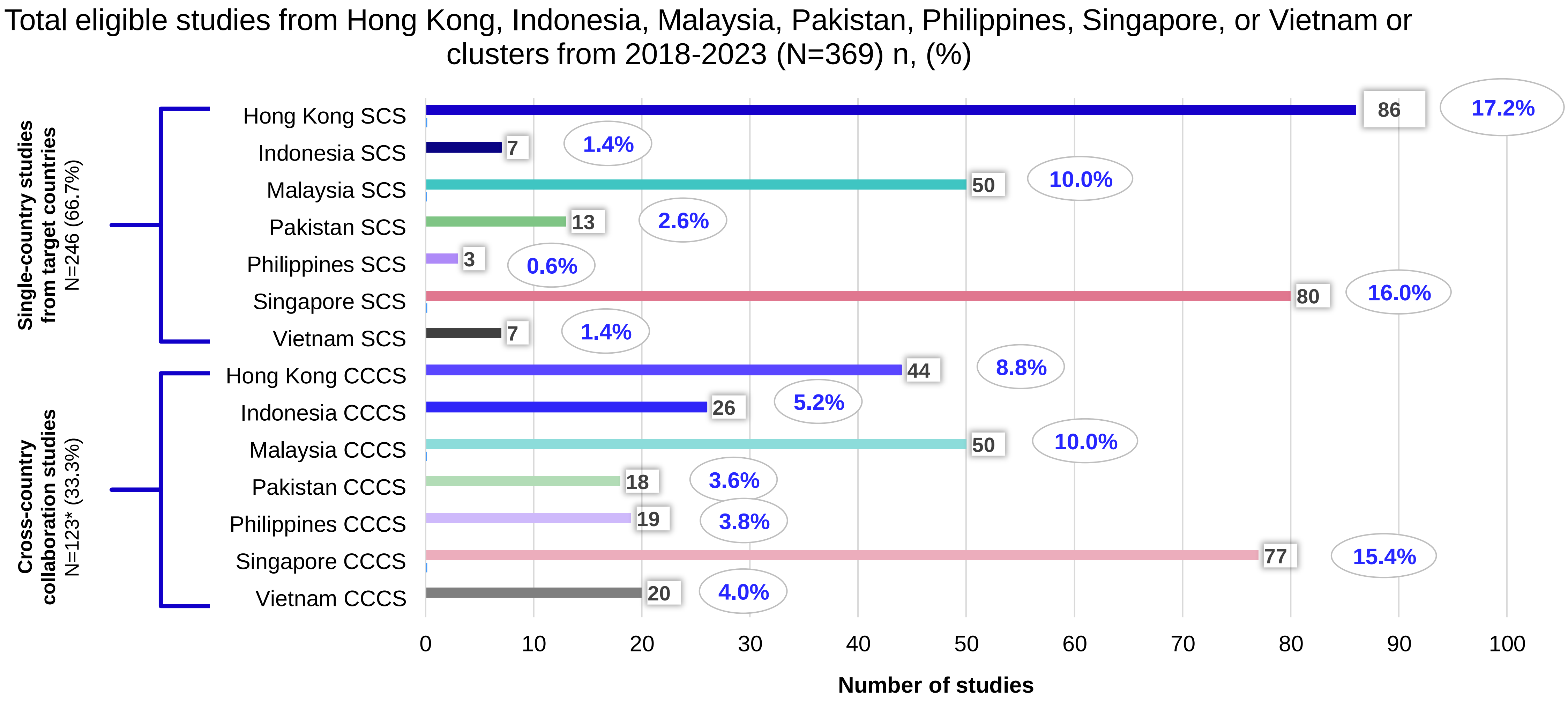

*Duplications in collaboration studies has been adjusted for the number of studies

Abbreviations: RWD, real-world data; SCS, single-country studies; CCCS, cross-country collaboration studies

Study types from integrated databases for all included studies

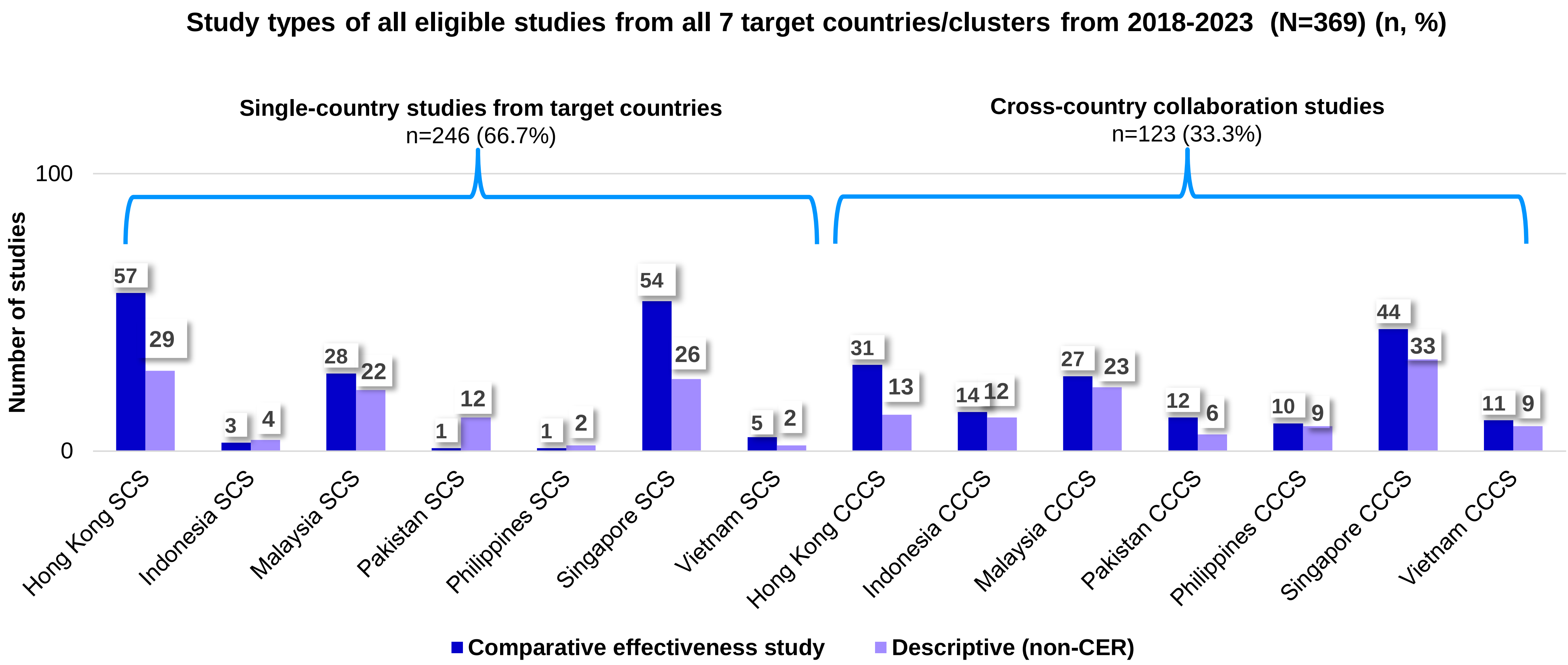

Hong Kong and Singapore have been contributing more CER than descriptive studies

Abbreviations: CER, comparative effectiveness research; RWD, real-world data

Time trend for CER for Hong Kong SCS & CCCS

Hong Kong SCS (*N* = 86)

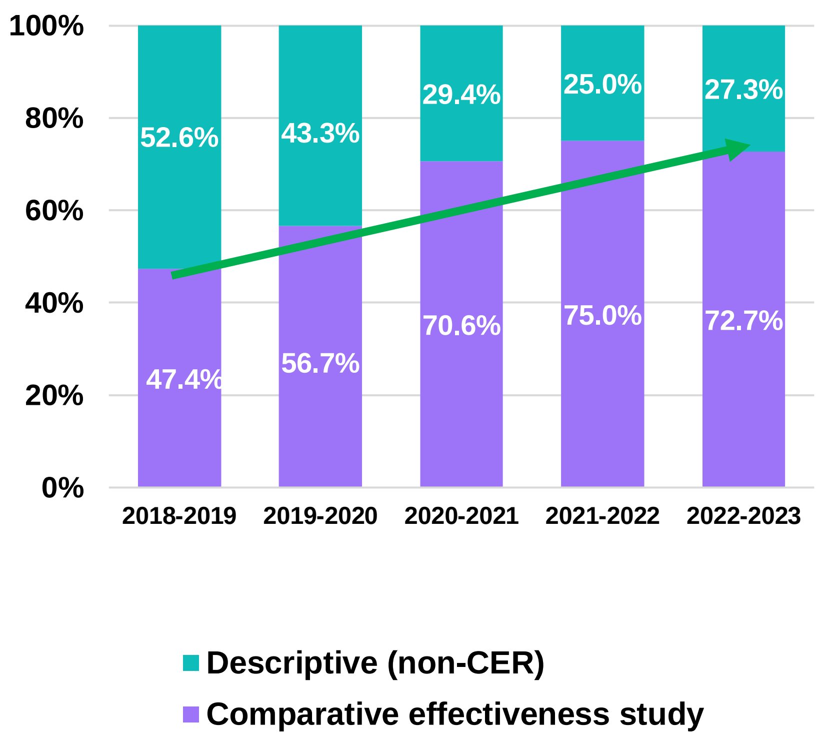

Hong Kong CCCS (*N* = 44)

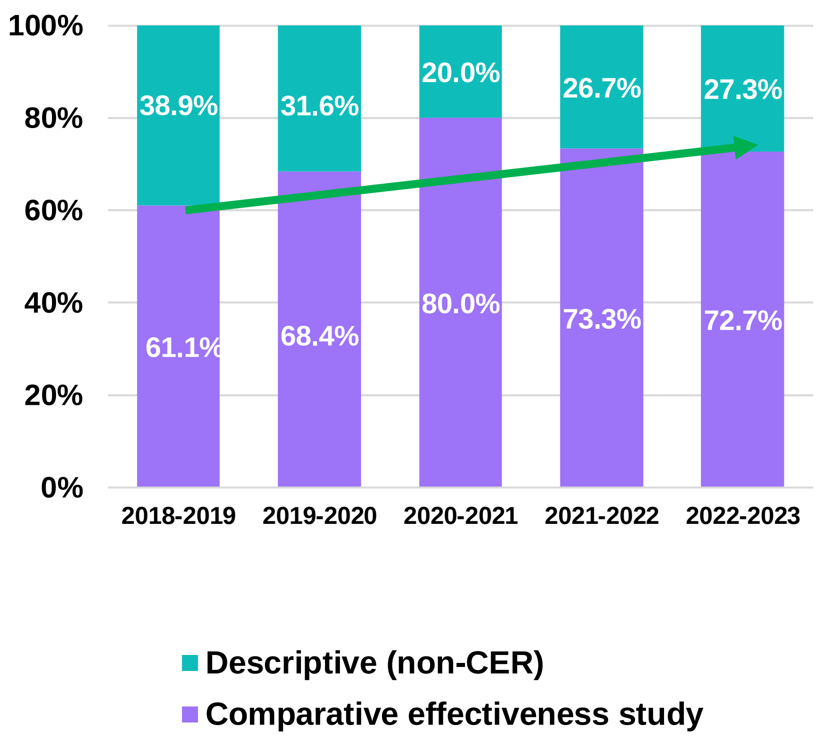

Hong Kong SCS & CCCS CER contribution have been increasing from 2018-2023

*As the PubMed final search was conducted on May 9, 2023, with filter for last 5 years, the eligible studies do not contain full data from 2018 and 2023. Study numbers from cross-country studies may appear as duplicates for studies conducted in multiple target countries.

Abbreviations: CER, comparative effectiveness research; RWD, real-world data; SCS, single country studies; CCCS, cross-country collaborative studies

Time trend for CER for Indonesia SCS & CCCS

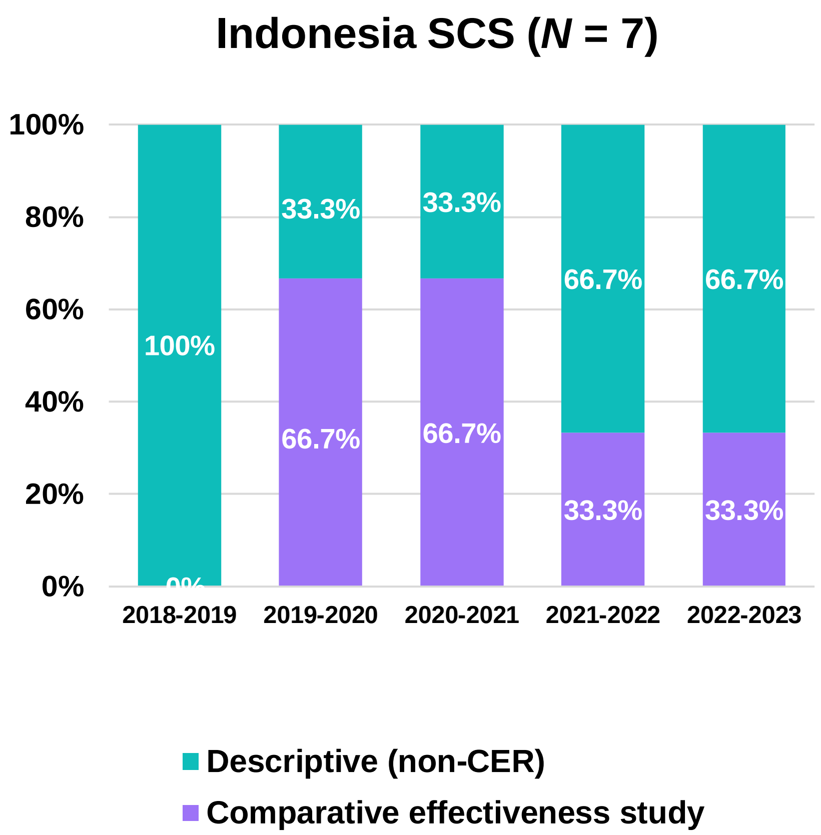

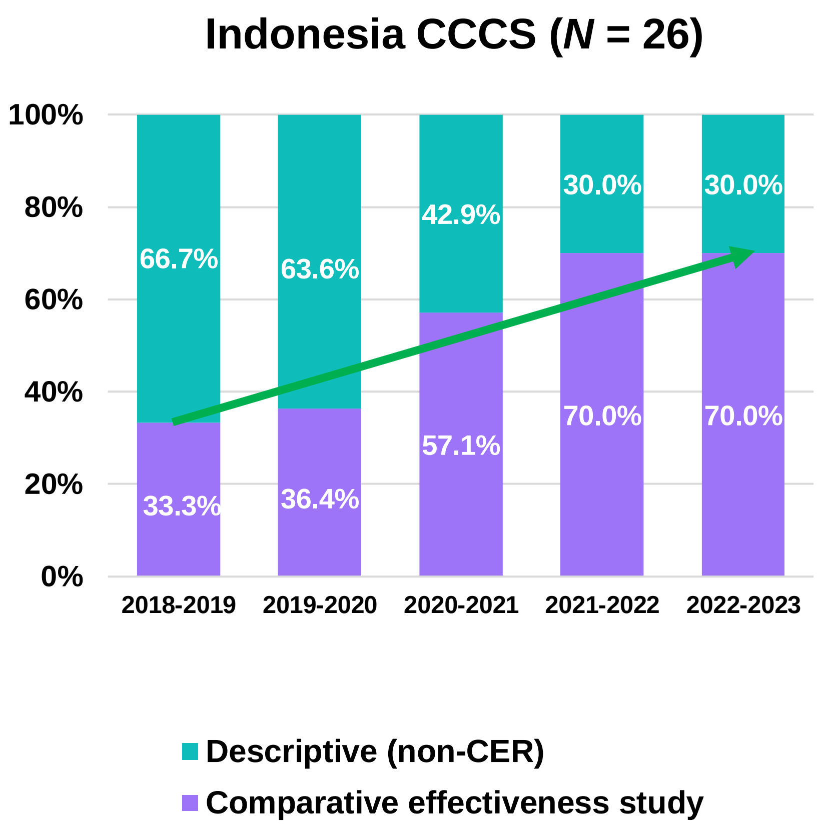

Indonesia CCCS CER contribution have been increasing from 2018-2023

*As the PubMed final search was conducted on May 9, 2023, with filter for last 5 years, the eligible studies do not contain full data from 2018 and 2023. Study numbers from cross-country studies may appear as duplicates for studies conducted in multiple target countries.

Abbreviations: CER, comparative effectiveness research; RWD, real-world data; SCS, single country studies; CCCS, cross-country collaborative studies

Time trend for CER for Malaysia SCS & CCCS

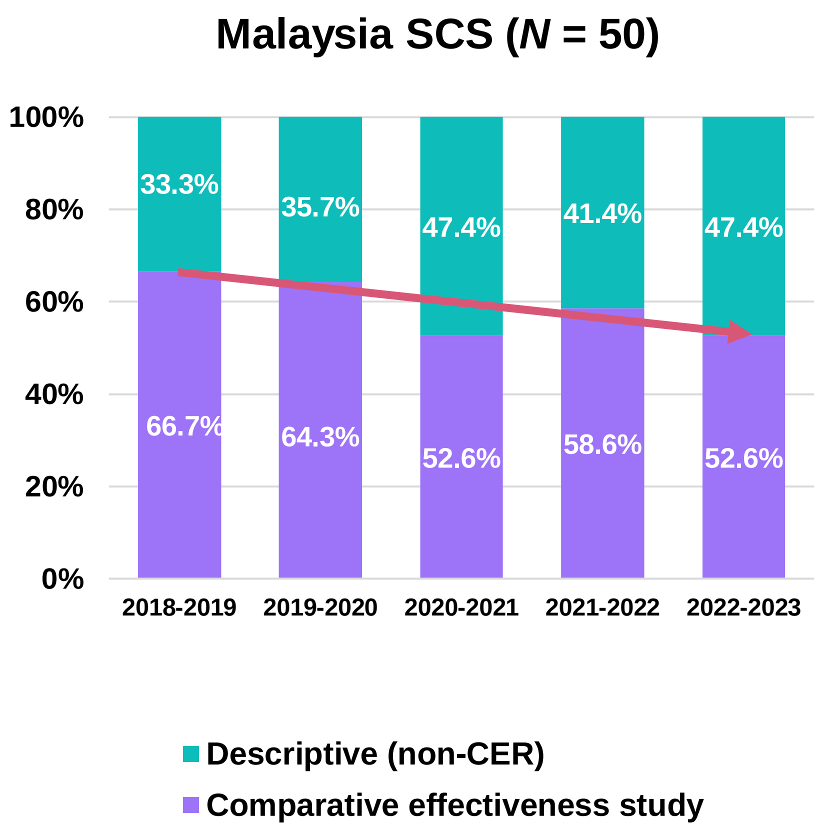

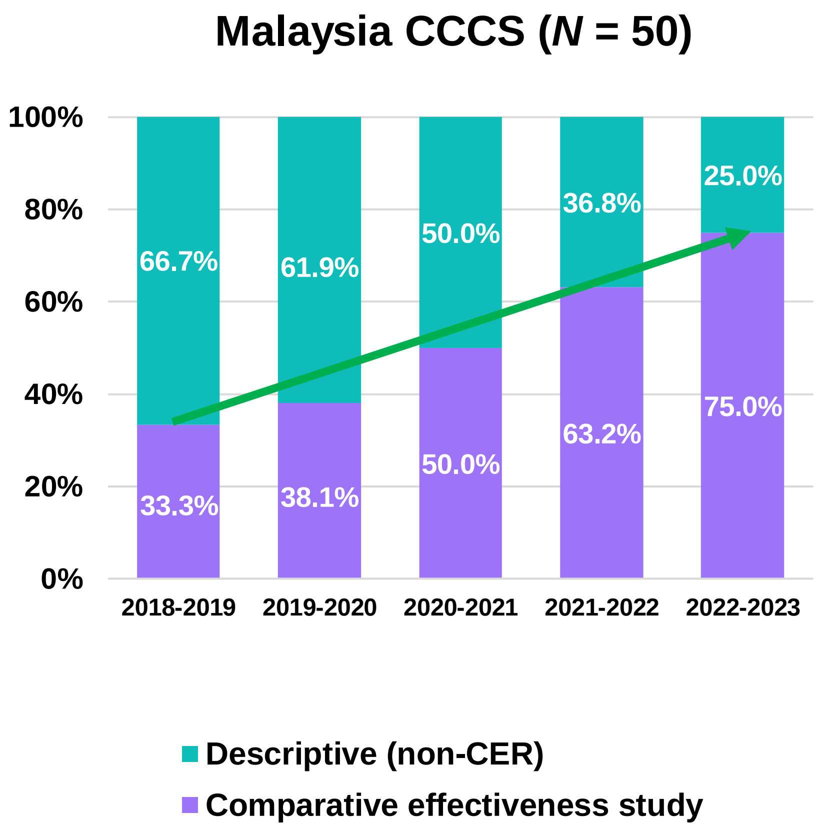

CER contribution of Malaysia SCS have been decreasing while CCCS have been increasing from 2018-2023

*As the PubMed final search was conducted on May 9, 2023, with filter for last 5 years, the eligible studies do not contain full data from 2018 and 2023. Study numbers from cross-country studies may appear as duplicates for studies conducted in multiple target countries.

Abbreviations: CER, comparative effectiveness research; RWD, real-world data; SCS, single country studies; CCCS, cross-country collaborative studies

Time trend for CER for Pakistan SCS & CCCS

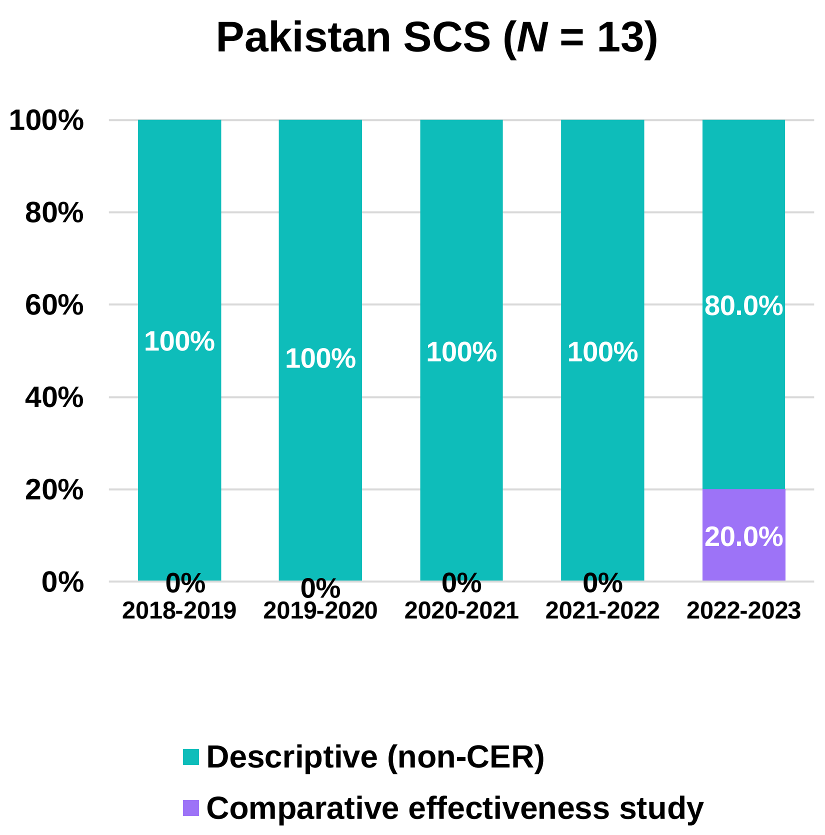

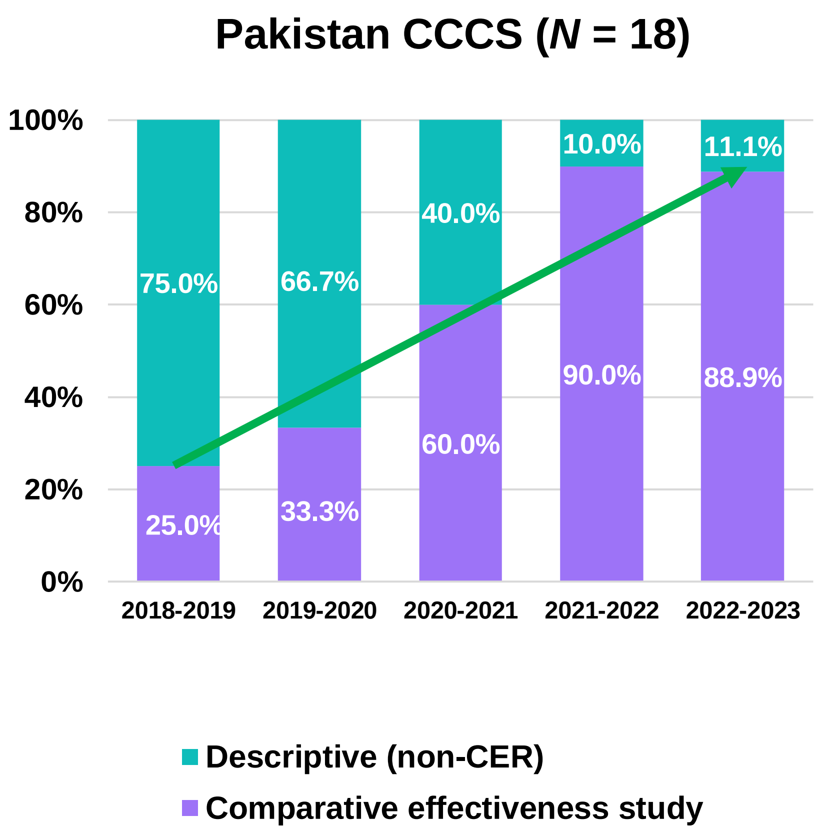

Pakistan CCCS CER contribution have been increasing from 2018-2023

*As the PubMed final search was conducted on May 9, 2023, with filter for last 5 years, the eligible studies do not contain full data from 2018 and 2023. Study numbers from cross-country studies may appear as duplicates for studies conducted in multiple target countries.

Abbreviations: CER, comparative effectiveness research; RWD, real-world data; SCS, single country studies; CCCS, cross-country collaborative studies

Time trend for CER for Philippines SCS & CCCS

Philippines SCS (*N* = 3)

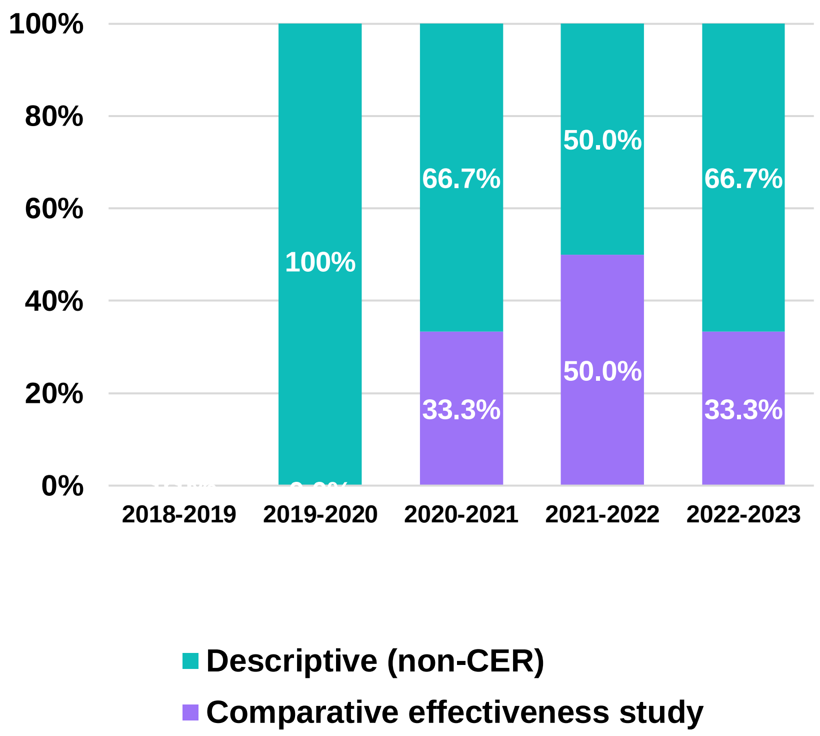

Philippines CCCS (*N* = 19)

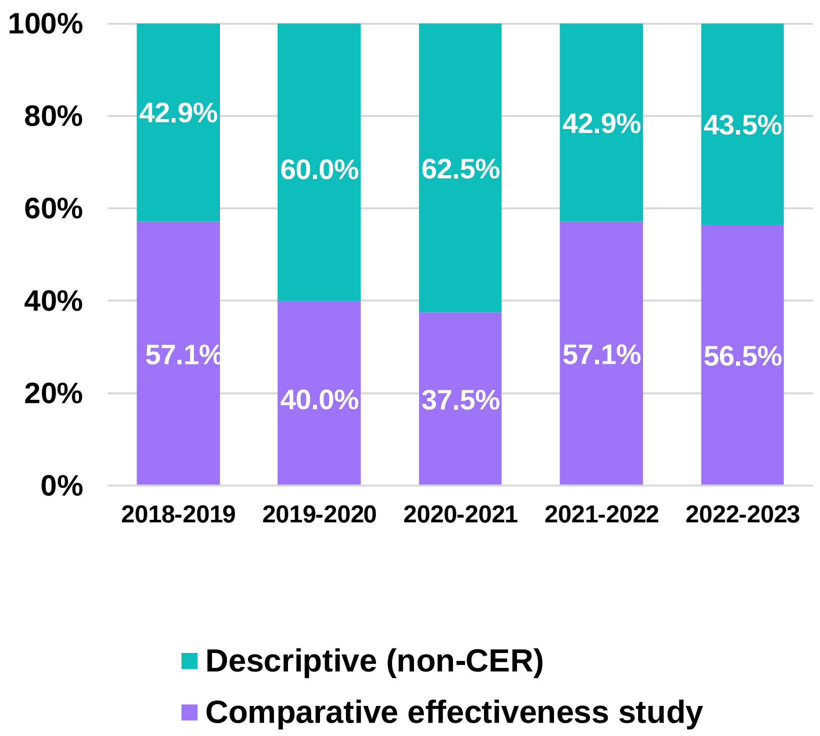

No consistent trend of CER contribution is observed in Philippines SCS and CCCS from 2018-2023

*As the PubMed final search was conducted on May 9, 2023, with filter for last 5 years, the eligible studies do not contain full data from 2018 and 2023. Study numbers from cross-country studies may appear as duplicates for studies conducted in multiple target countries.

Abbreviations: CER, comparative effectiveness research; RWD, real-world data; SCS, single country studies; CCCS, cross-country collaborative studies

Time trend for CER for Singapore SCS & CCCS

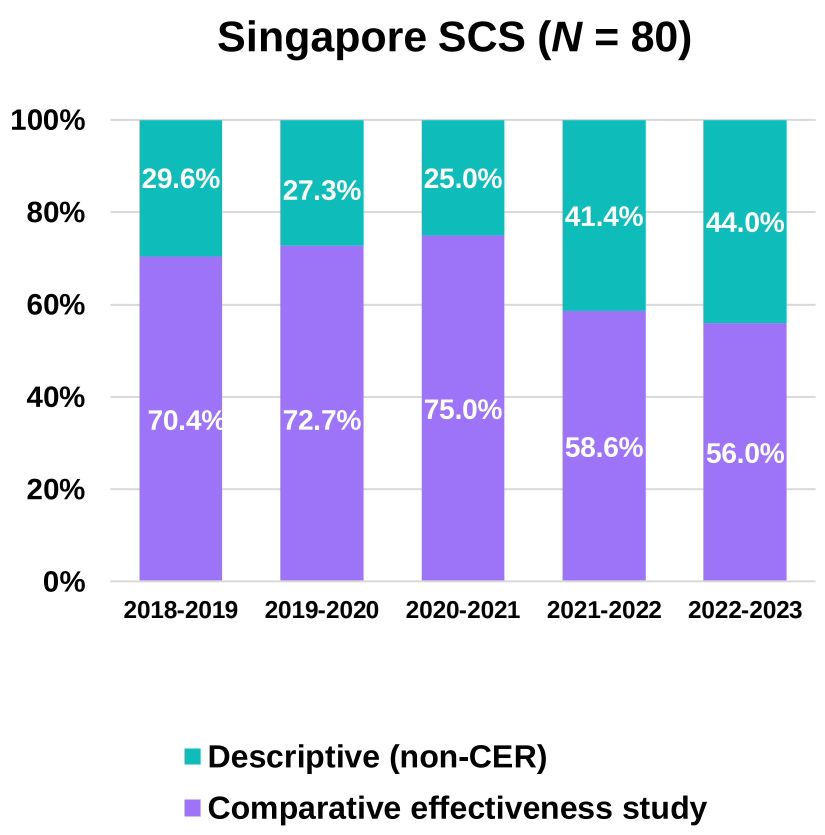

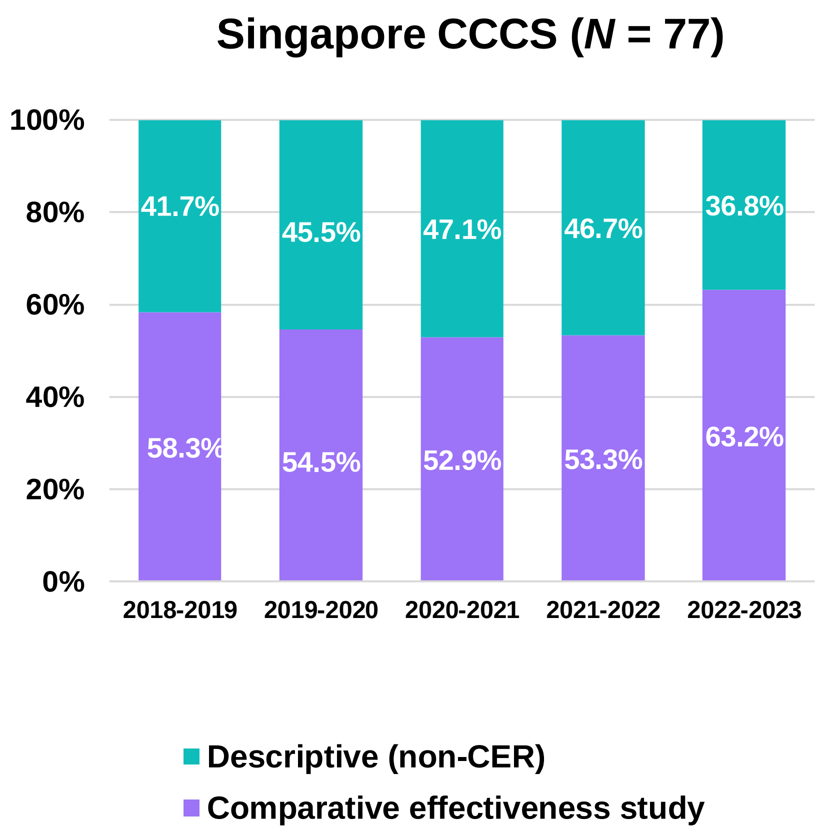

CER contribution of Singapore SCS dropped from 2021-2023 while CCCS is flat from 2018-2023

*As the PubMed final search was conducted on May 9, 2023, with filter for last 5 years, the eligible studies do not contain full data from 2018 and 2023. Study numbers from cross-country studies may appear as duplicates for studies conducted in multiple target countries.

Abbreviations: CER, comparative effectiveness research; RWD, real-world data; SCS, single country studies; CCCS, cross-country collaborative studies

Time trend for CER for Vietnam SCS & CCCS

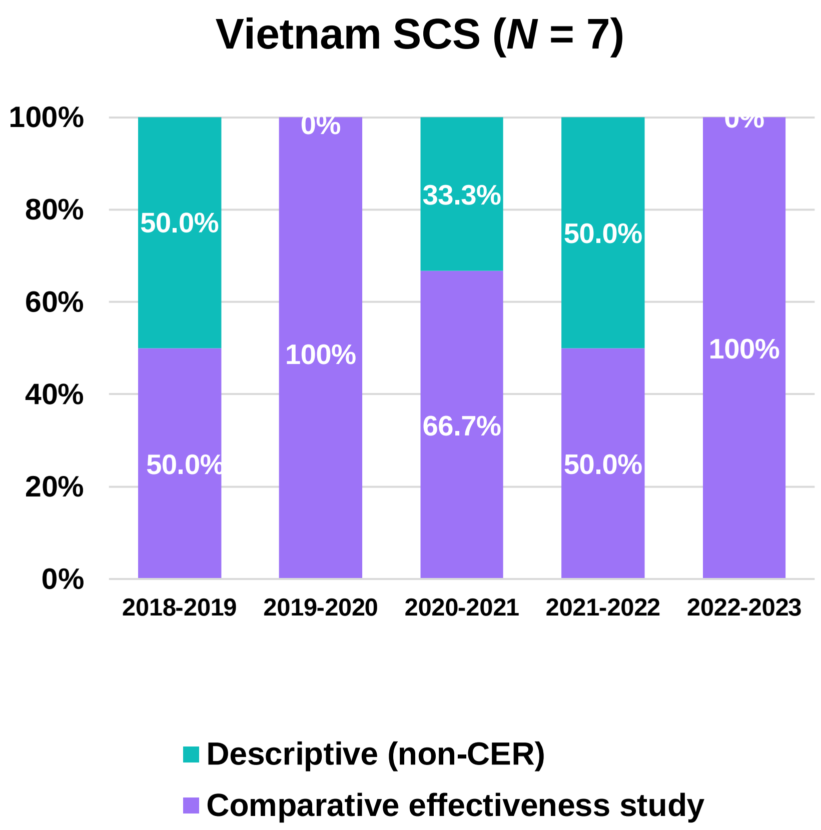

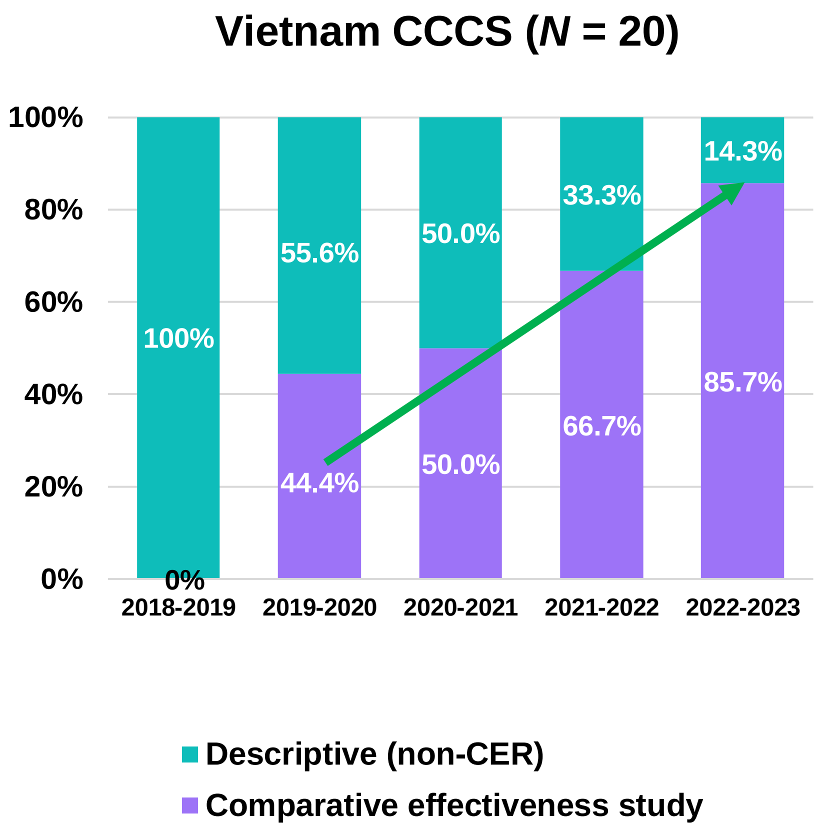

Vietnam CCCS CER contribution have been increasing from 2018-2023

*As the PubMed final search was conducted on May 9, 2023, with filter for last 5 years, the eligible studies do not contain full data from 2018 and 2023. Study numbers from cross-country studies may appear as duplicates for studies conducted in multiple target countries.

Abbreviations: CER, comparative effectiveness research; RWD, real-world data; SCS, single country studies; CCCS, cross-country collaborative studies

Single exclusive database utilization for RWD studies from total target countries (SCS+CCS Combined)

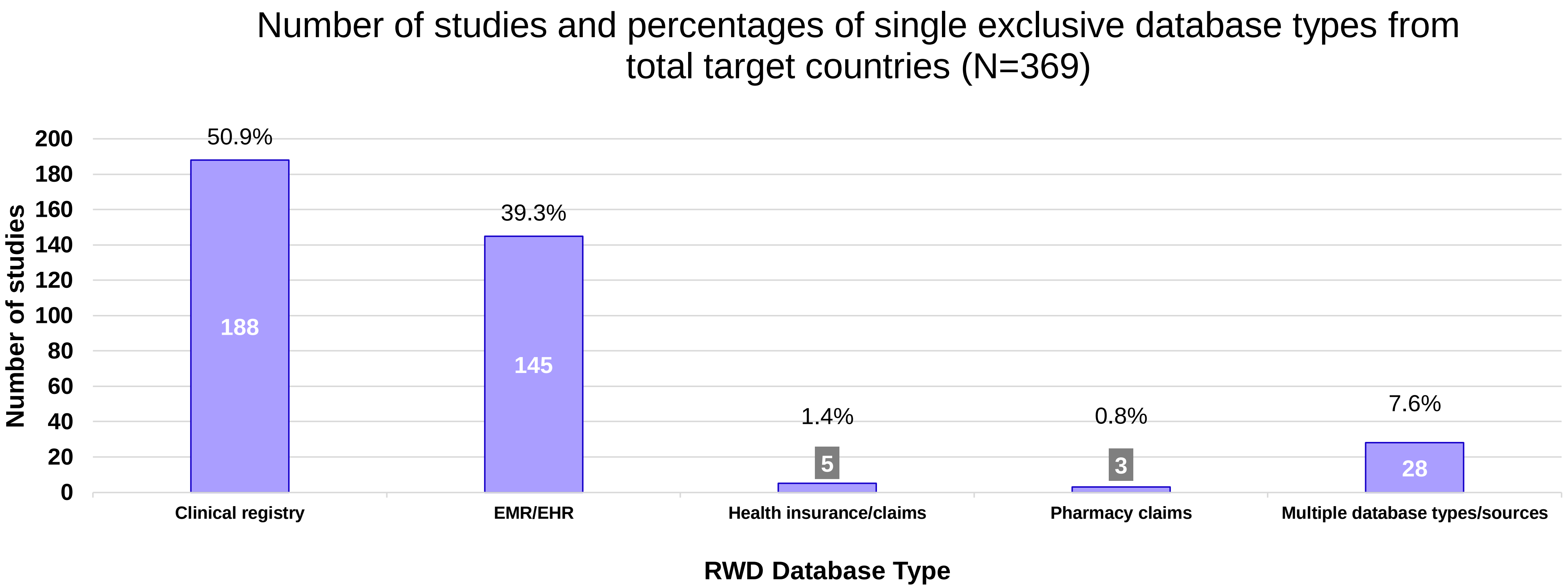

Abbreviations: RWD, real-world data; EMR/EHR, electronic medical record/electronic health record; SCS, Single-country studies; CCCS, Cross-country collaborative studies

Single exclusive database utilization for SCS studies from target countries

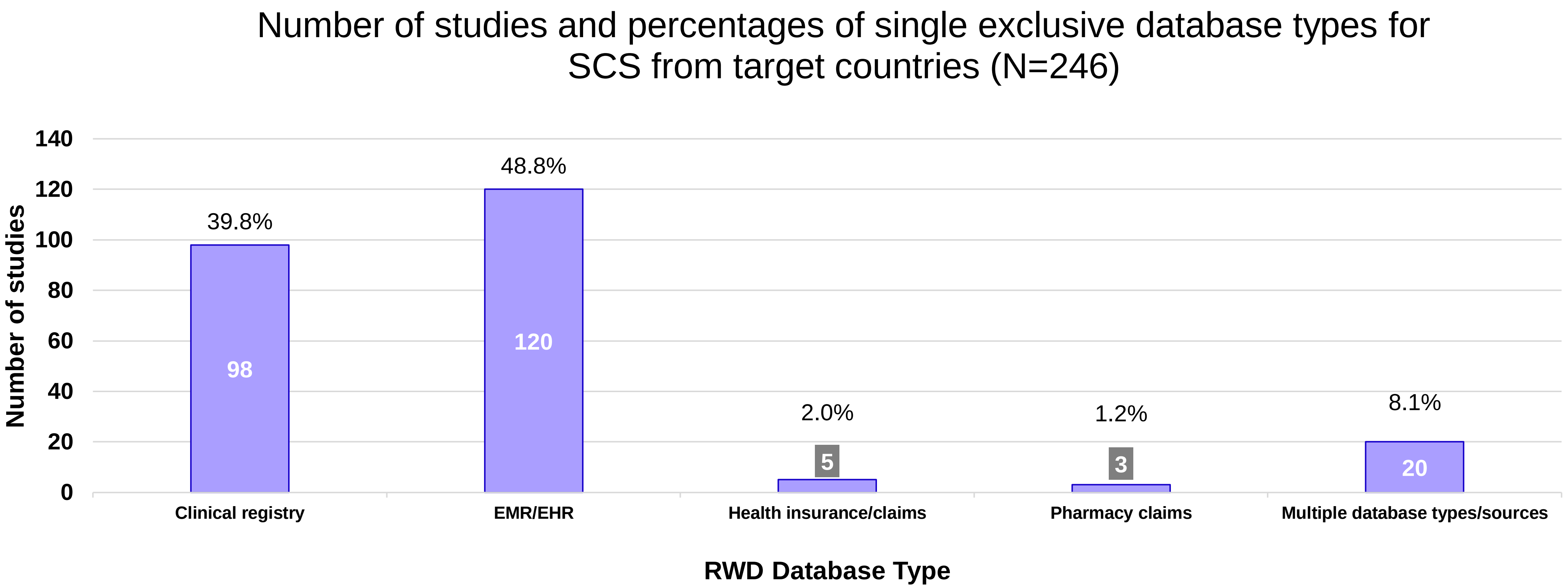

Abbreviations: RWD, real-world data; EMR/EHR, electronic medical record/electronic health record; SCS, Single-country studies; CCCS, Cross-country collaborative studies

Single exclusive database utilization for CCCS studies from target countries

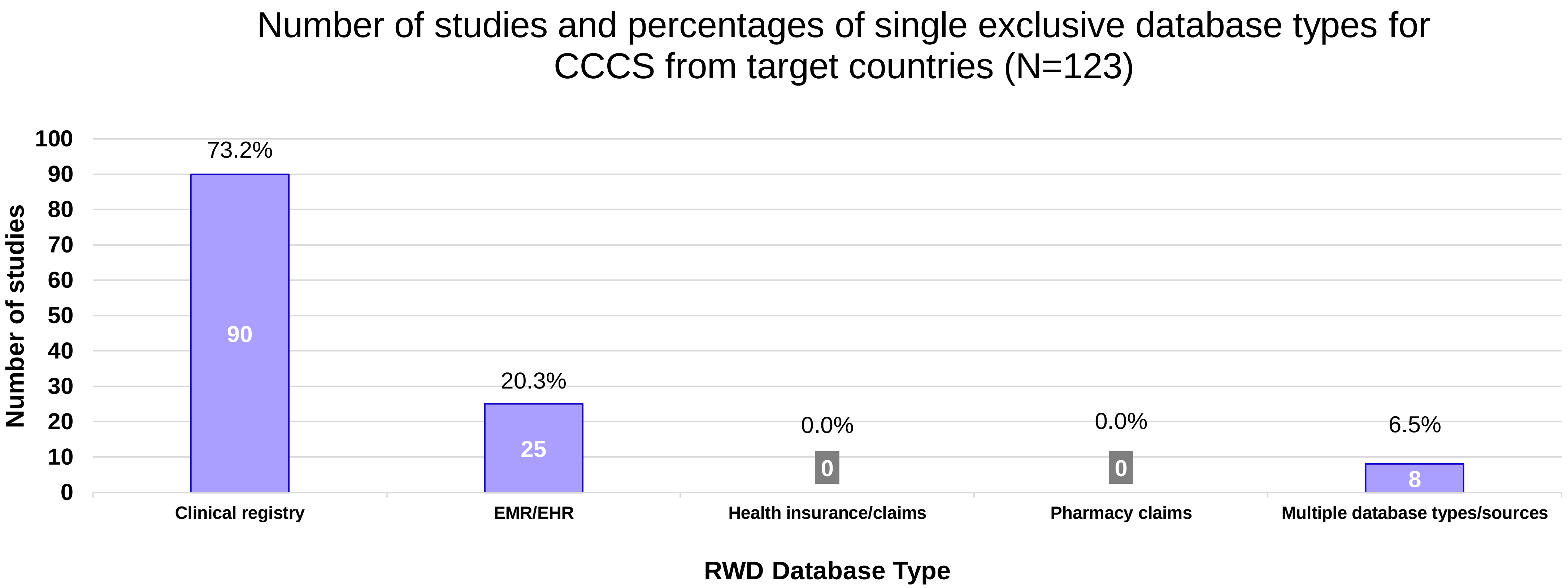

Abbreviations: RWD, real-world data; EMR/EHR, electronic medical record/electronic health record; SCS, Single-country studies; CCCS, Cross-country collaborative studies

Percentage of single exclusive database utilization for RWD studies from single-country studies of target countries

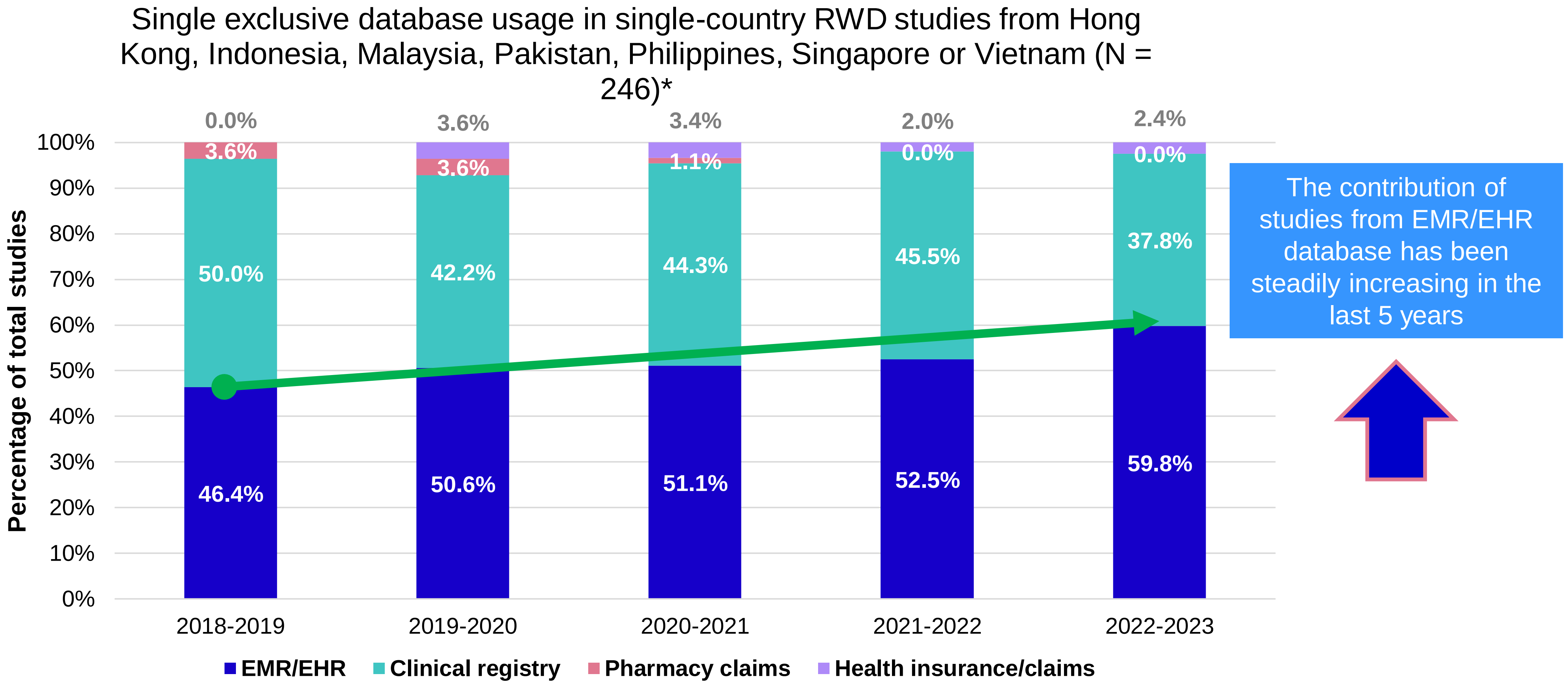

*Out of 246 studies in target countries, 226 has a single database type/source, while 20 has a combination of one or more database type/source.

Abbreviations: RWD, real-world data; EMR/EHR, electronic medical record/electronic health record

Percentage of single exclusive database utilization for RWD studies from cross-country collaborative studies of target countries

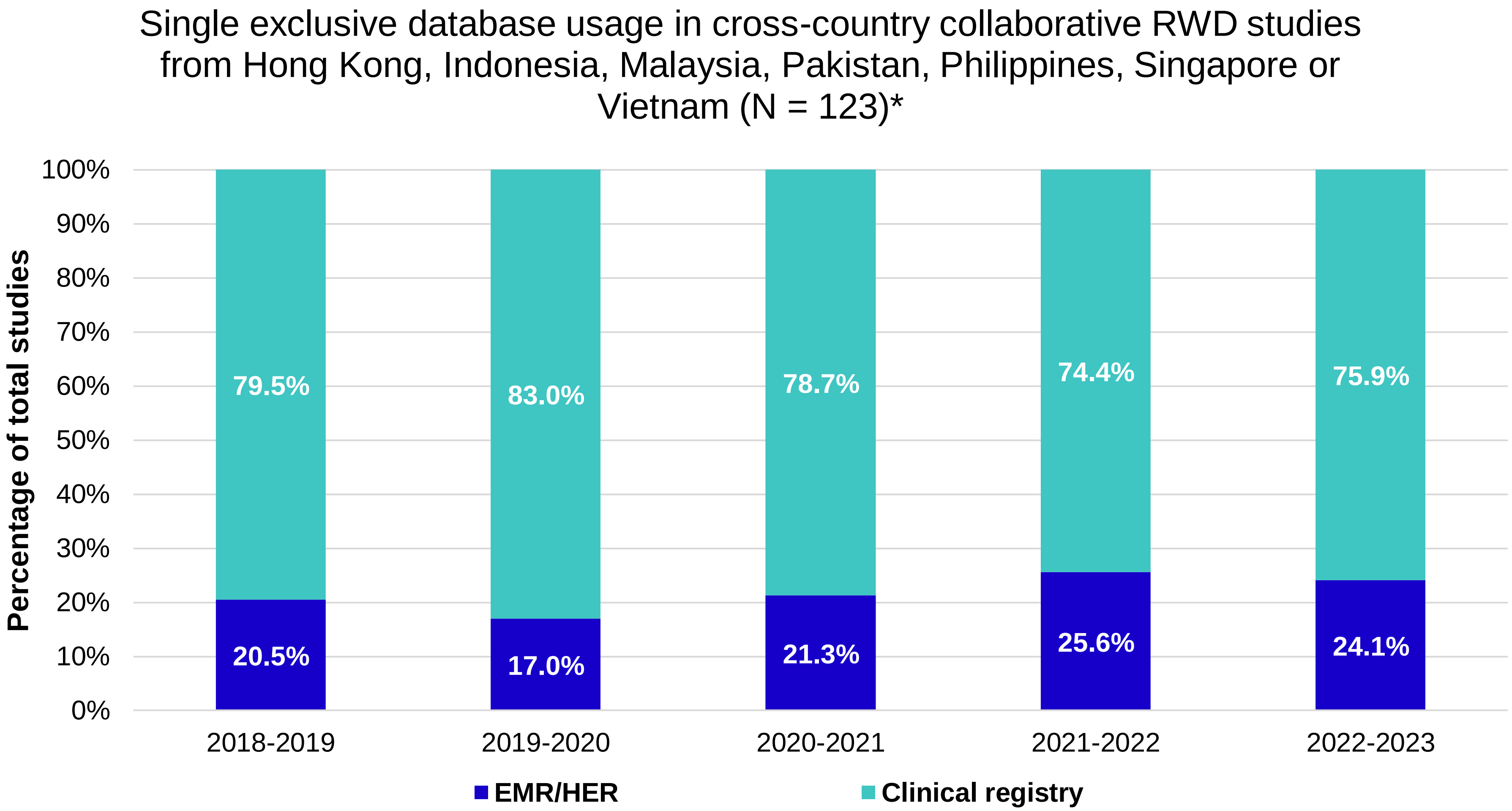

*Out of 123 studies in target countries, 115 has a single database type/source, while 8 has a combination of one or more database type/source. Study numbers from cross-country studies may appear as duplicates for studies conducted in multiple target countries. No study used the health insurance/claims database exclusively.

Abbreviations: RWD, real-world data; EMR/EHR, electronic medical record/electronic health record

Distribution and time trend for the percentage of studies conducted in different medical areas (N = 369)

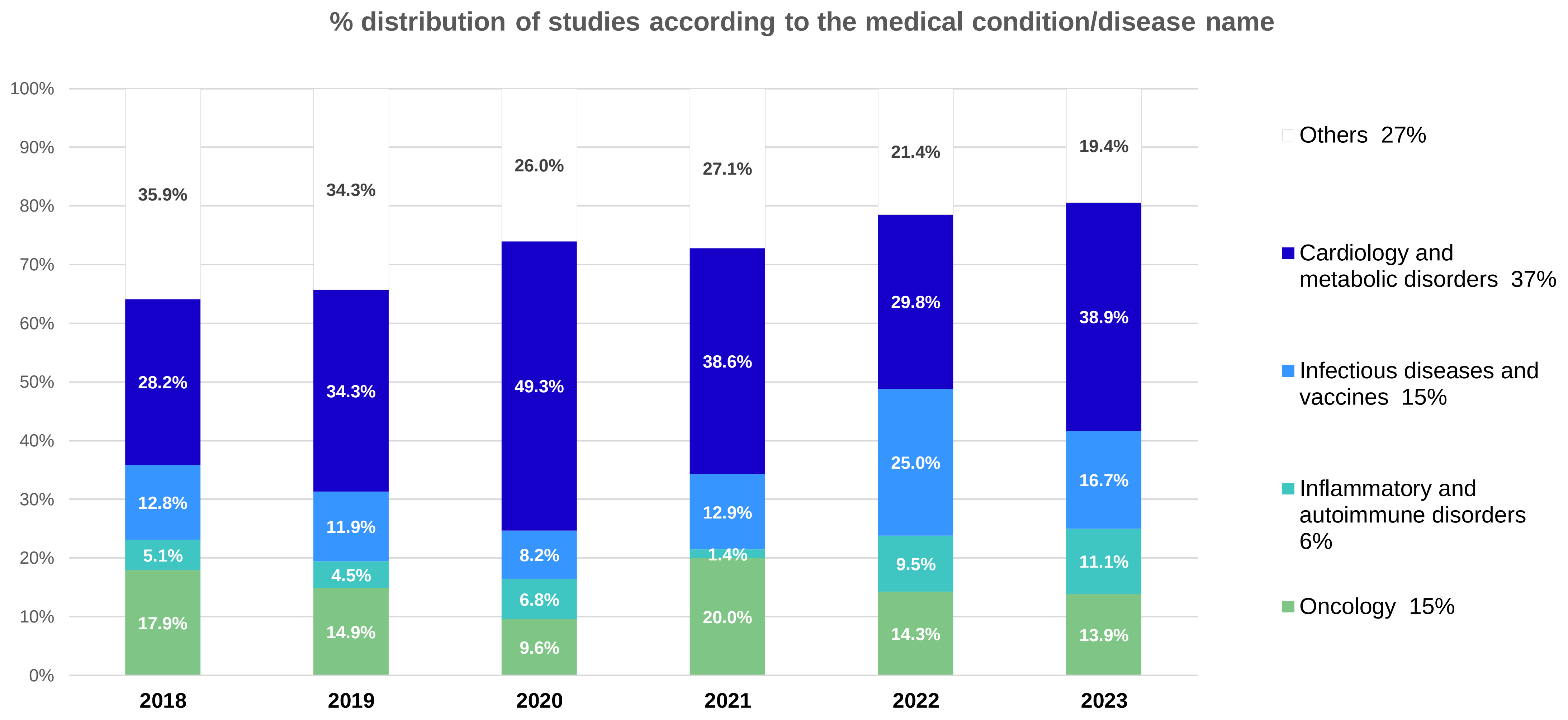

Study duration from integrated databases of total target countries (SCS & CCCS combined)

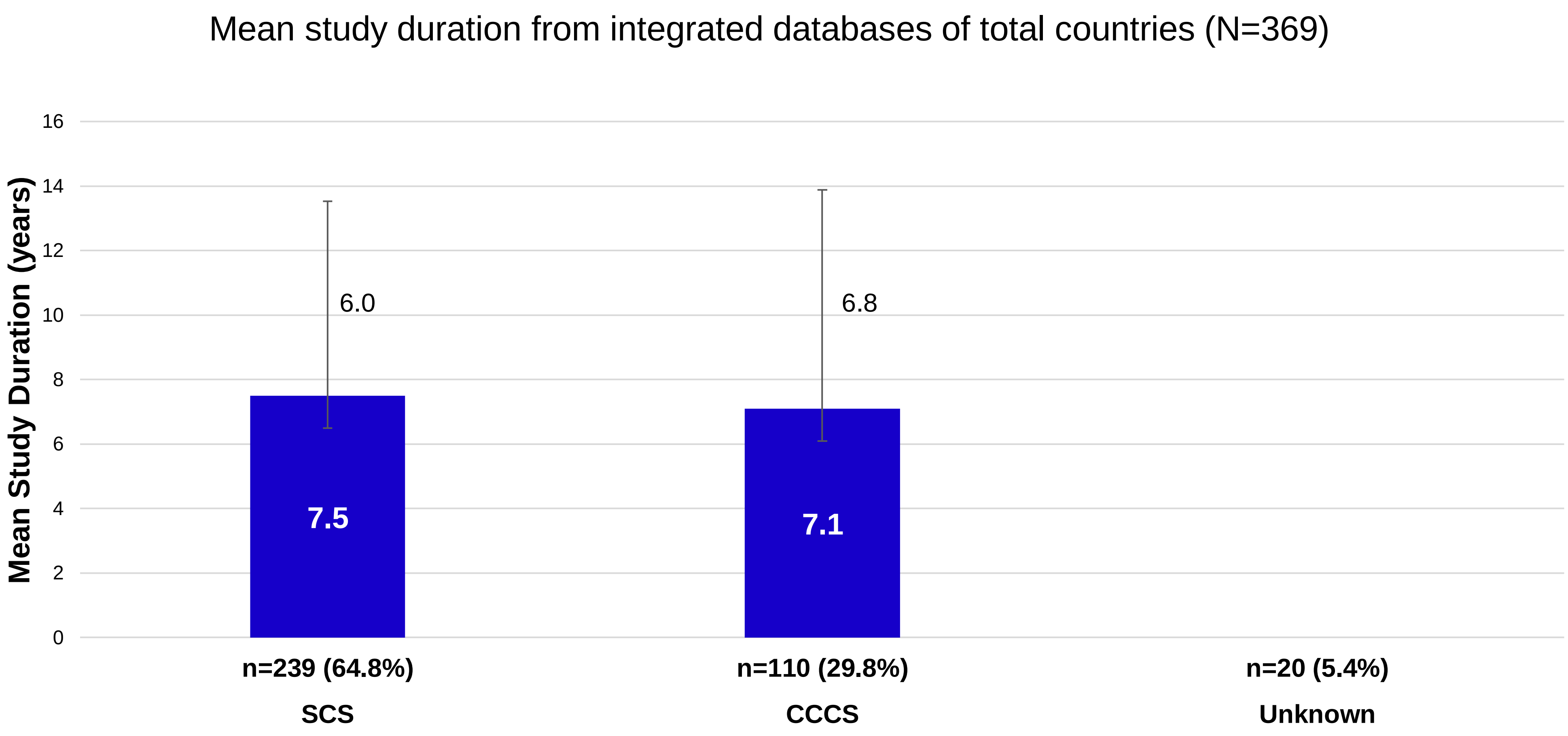

Abbreviations: SCS, Single-country studies; CCCS, Cross-country collaborative studies

Study duration from integrated databases of Hong Kong SCS & CCCS from 2018-2023

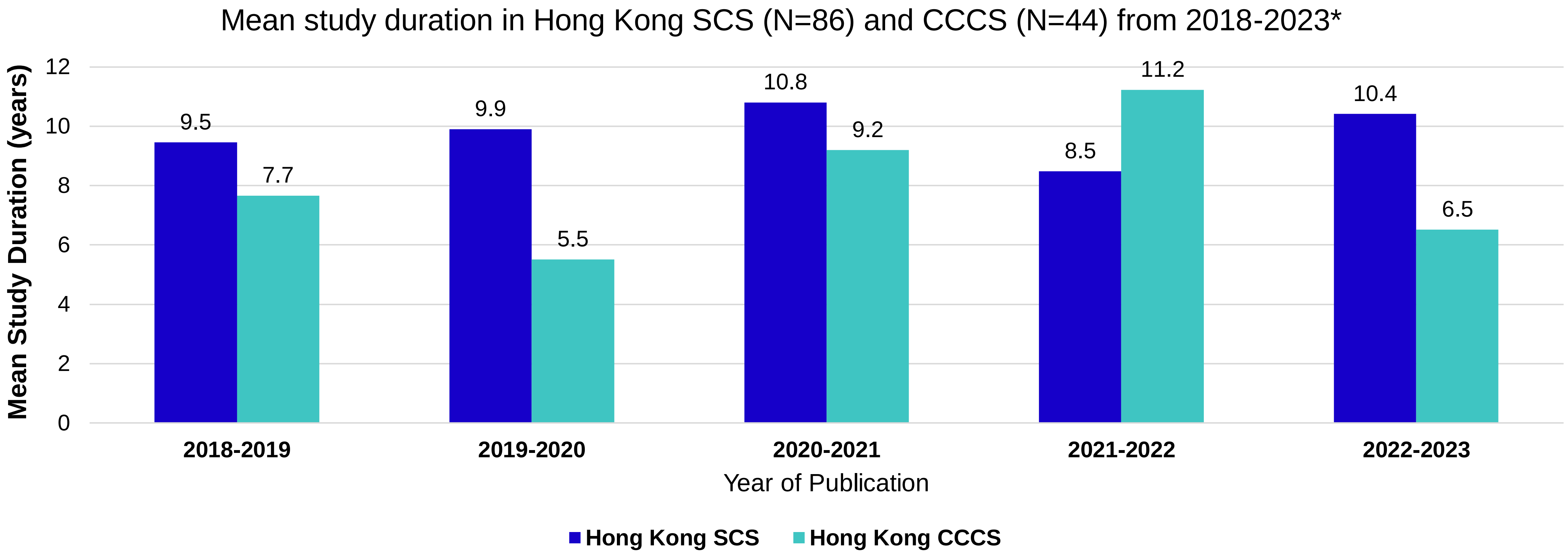

No consistent trend of mean study duration observed in Hong Kong SCS and CCCS from 2018-2023

*As the PubMed final search was conducted on May 9, 2023, with filter for last 5 years, the eligible studies do not contain full data from 2018 and 2023. Study numbers from cross-country studies may appear as duplicates for studies conducted in multiple target countries. The moving average of study duration is calculated by averaging the mean of study duration in each consecutive year.

Study duration from integrated databases of Indonesia SCS & CCCS from 2018-2023

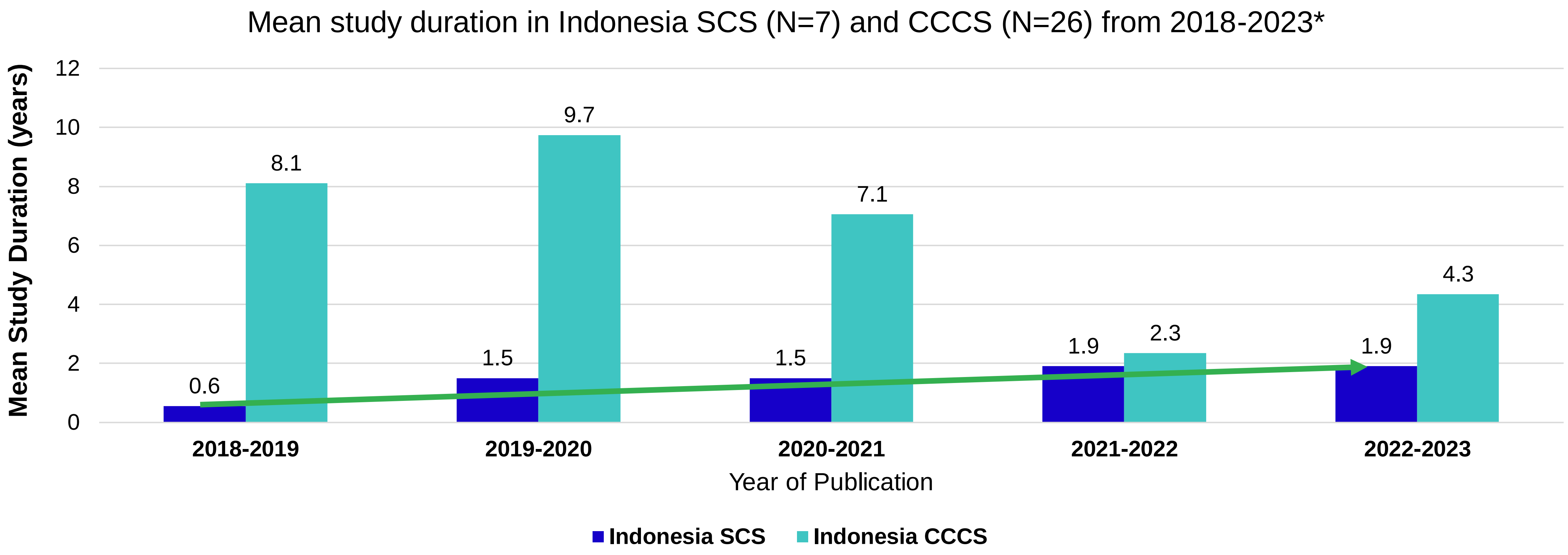

The mean study duration for Indonesia SCS has been increasing from 2018-2023

*As the PubMed final search was conducted on May 9, 2023, with filter for last 5 years, the eligible studies do not contain full data from 2018 and 2023. Study numbers from cross-country studies may appear as duplicates for studies conducted in multiple target countries. The moving average of study duration is calculated by averaging the mean of study duration in each consecutive year.

Study duration from integrated databases of Malaysia SCS & CCCS from 2018-2023

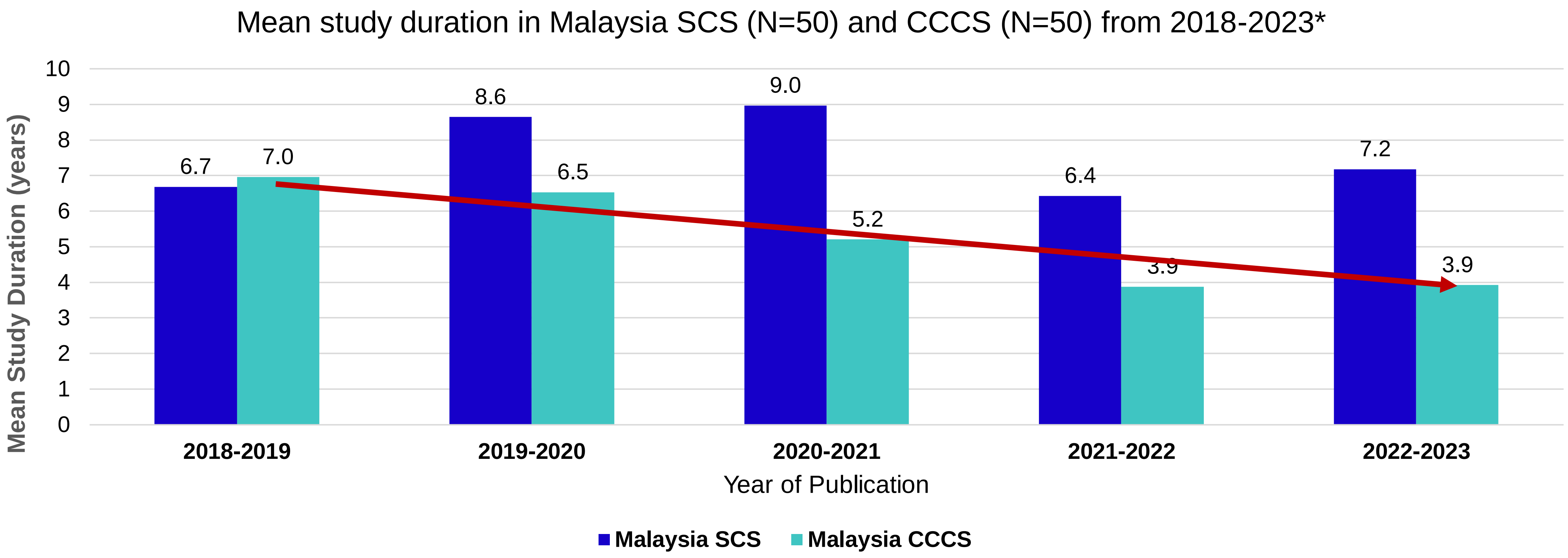

The mean study duration in Malaysia CCCS have been decreasing from 2018-2023

*As the PubMed final search was conducted on May 9, 2023, with filter for last 5 years, the eligible studies do not contain full data from 2018 and 2023. Study numbers from cross-country studies may appear as duplicates for studies conducted in multiple target countries. The moving average of study duration is calculated by averaging the mean of study duration in each consecutive year.

Study duration from integrated databases of Pakistan SCS & CCCS from 2018-2023

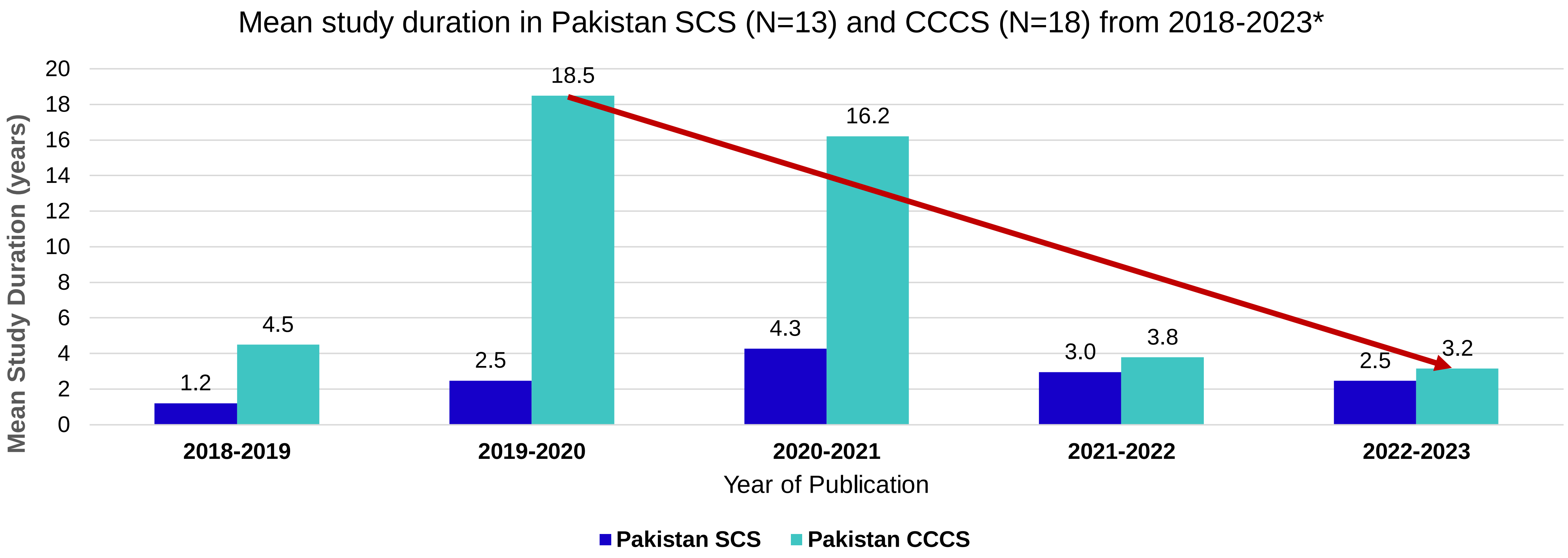

The mean study duration in Pakistan CCCS have been decreasing from 2019-2023

*As the PubMed final search was conducted on May 9, 2023, with filter for last 5 years, the eligible studies do not contain full data from 2018 and 2023. Study numbers from cross-country studies may appear as duplicates for studies conducted in multiple target countries. The moving average of study duration is calculated by averaging the mean of study duration in each consecutive year.

Study duration from integrated databases of Philippines SCS & CCCS from 2018-2023

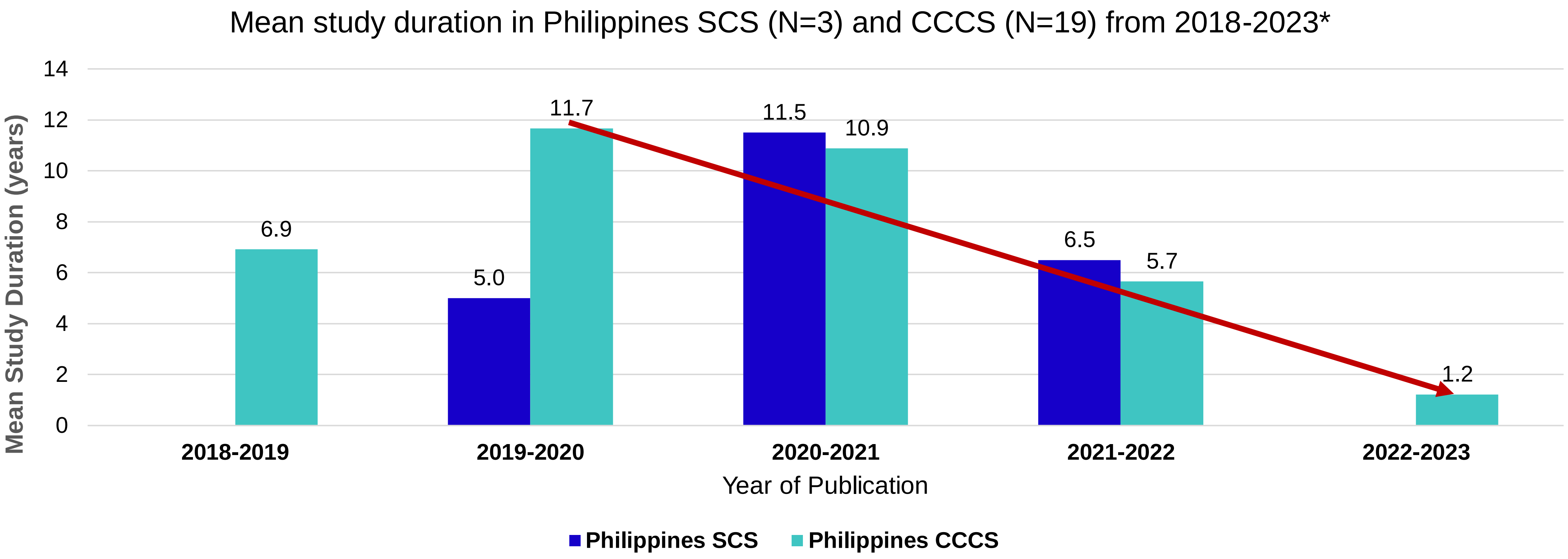

The mean study duration for Philippines CCCS have been decreasing from 2019-2023

*As the PubMed final search was conducted on May 9, 2023, with filter for last 5 years, the eligible studies do not contain full data from 2018 and 2023. Study numbers from cross-country studies may appear as duplicates for studies conducted in multiple target countries. The moving average of study duration is calculated by averaging the mean of study duration in each consecutive year.

Study duration from integrated databases of Singapore SCS & CCCS from 2018-2023

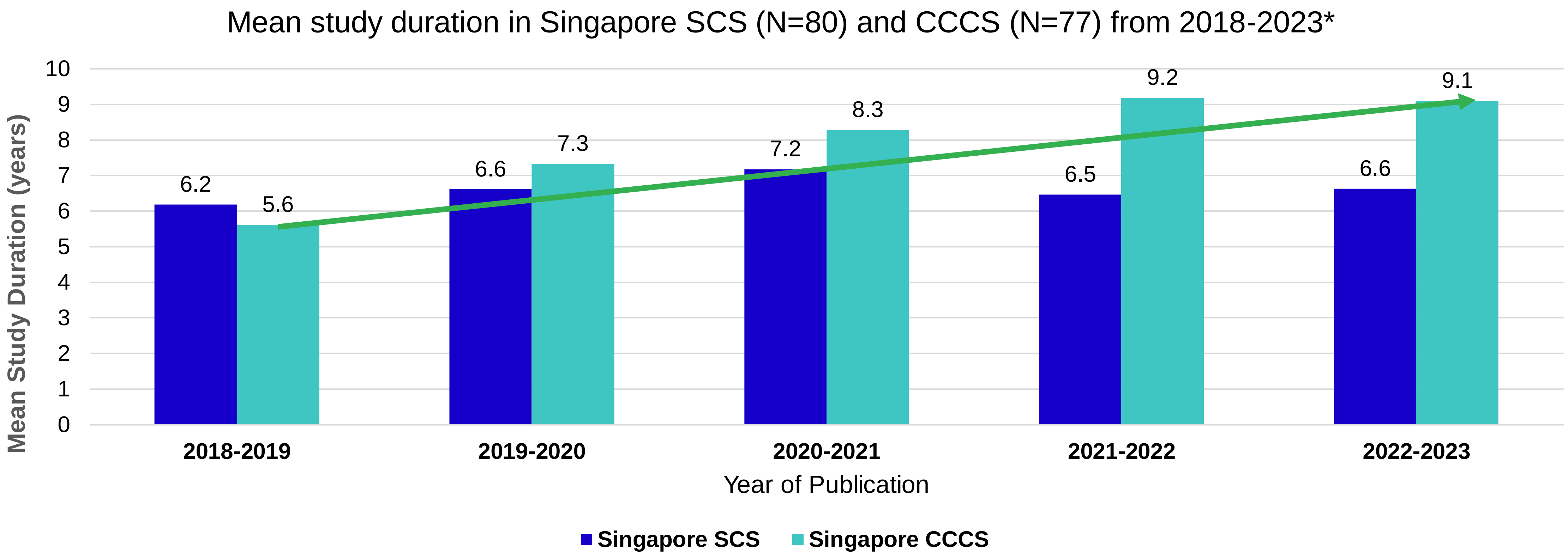

The mean study duration in Singapore CCCS have been increasing from 2018-2023

*As the PubMed final search was conducted on May 9, 2023, with filter for last 5 years, the eligible studies do not contain full data from 2018 and 2023. Study numbers from cross-country studies may appear as duplicates for studies conducted in multiple target countries. The moving average of study duration is calculated by averaging the mean of study duration in each consecutive year.

Study duration from integrated databases of Vietnam SCS & CCCS from 2018-2023

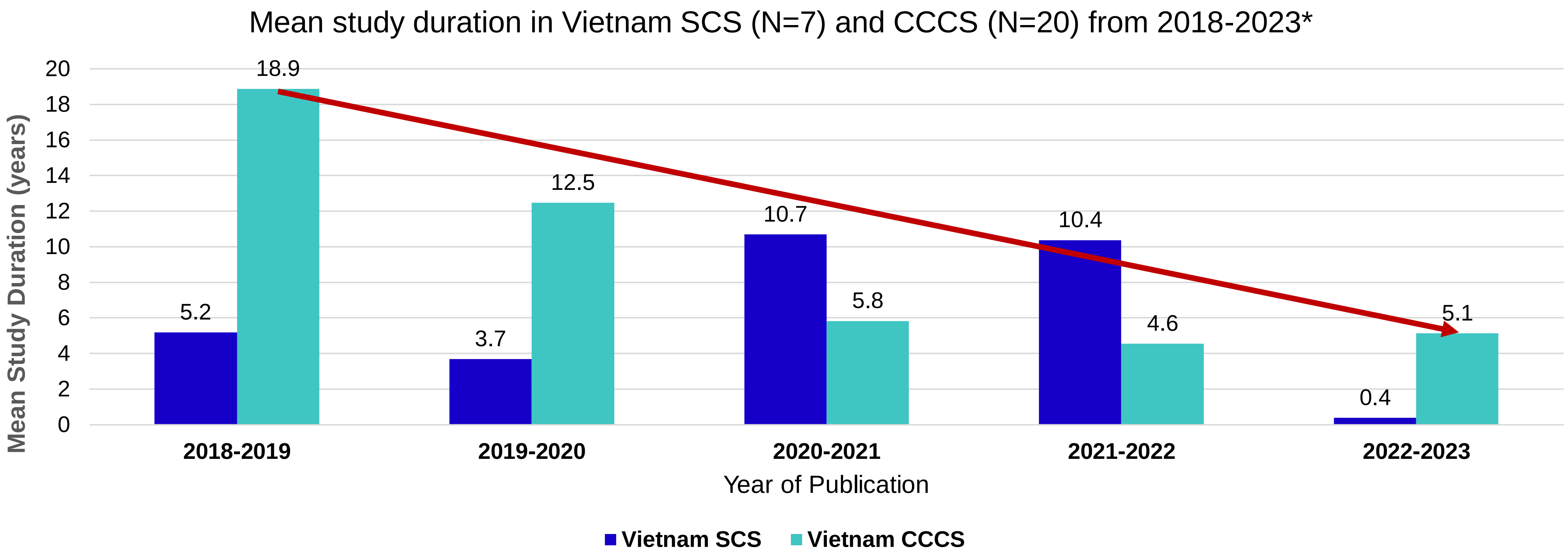

The mean study duration in Vietnam CCCS have been decreasing from 2018-2023

*As the PubMed final search was conducted on May 9, 2023, with filter for last 5 years, the eligible studies do not contain full data from 2018 and 2023. Study numbers from cross-country studies may appear as duplicates for studies conducted in multiple target countries. The moving average of study duration is calculated by averaging the mean of study duration in each consecutive year.

Publication time lag as per the number of RWD studies for all integrated databases

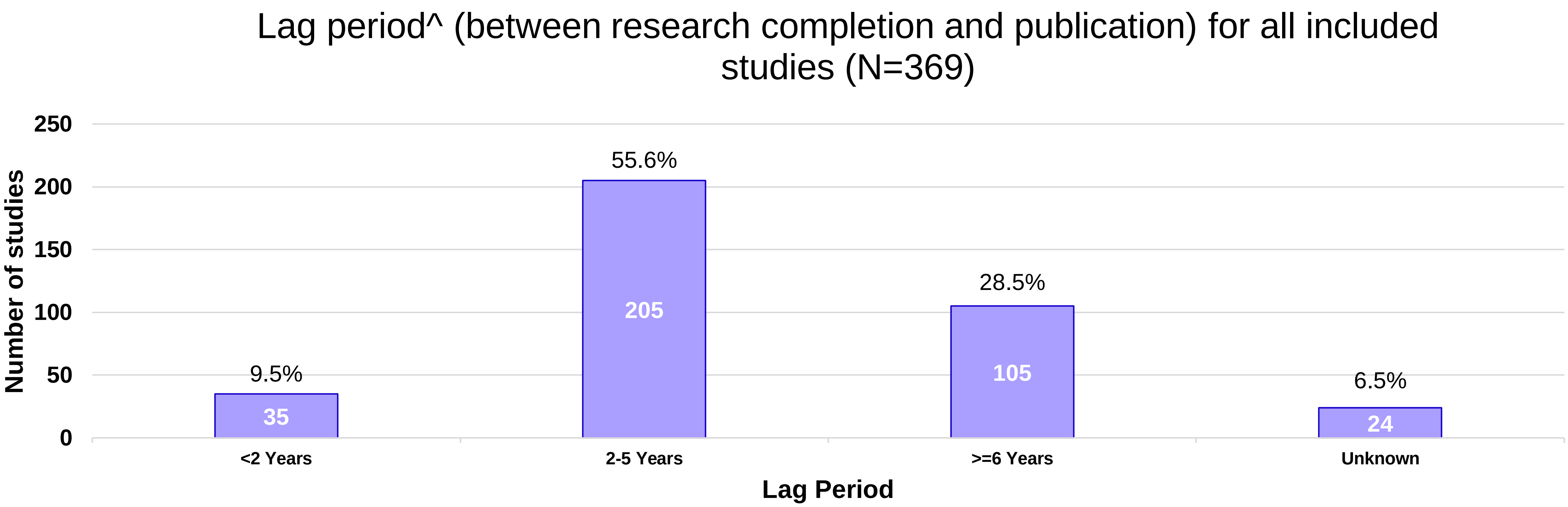

Majority of RWD studies have a lag period of 2-5 years between research completion and publication

^The lag period is defined as follows:
<2 Years (for publications published within 2 years after the research completion),
2-5 Years (for publications published within 2 to 5 years after the research completion), >=6 Years (for publications published 6 years and above after the research completion).
Unknown is for publications with an unspecified year of the research completion.

Abbreviations: RWD, real-world data

Trend for publication time lag from the integrated databases of single-country studies from target countries from 2018-2023

Time trend for percentage of singe-country studies of each lag period^ from Hong Kong, Indonesia, Malaysia, Pakistan, Philippines, Singapore or Vietnam (N = 246)*

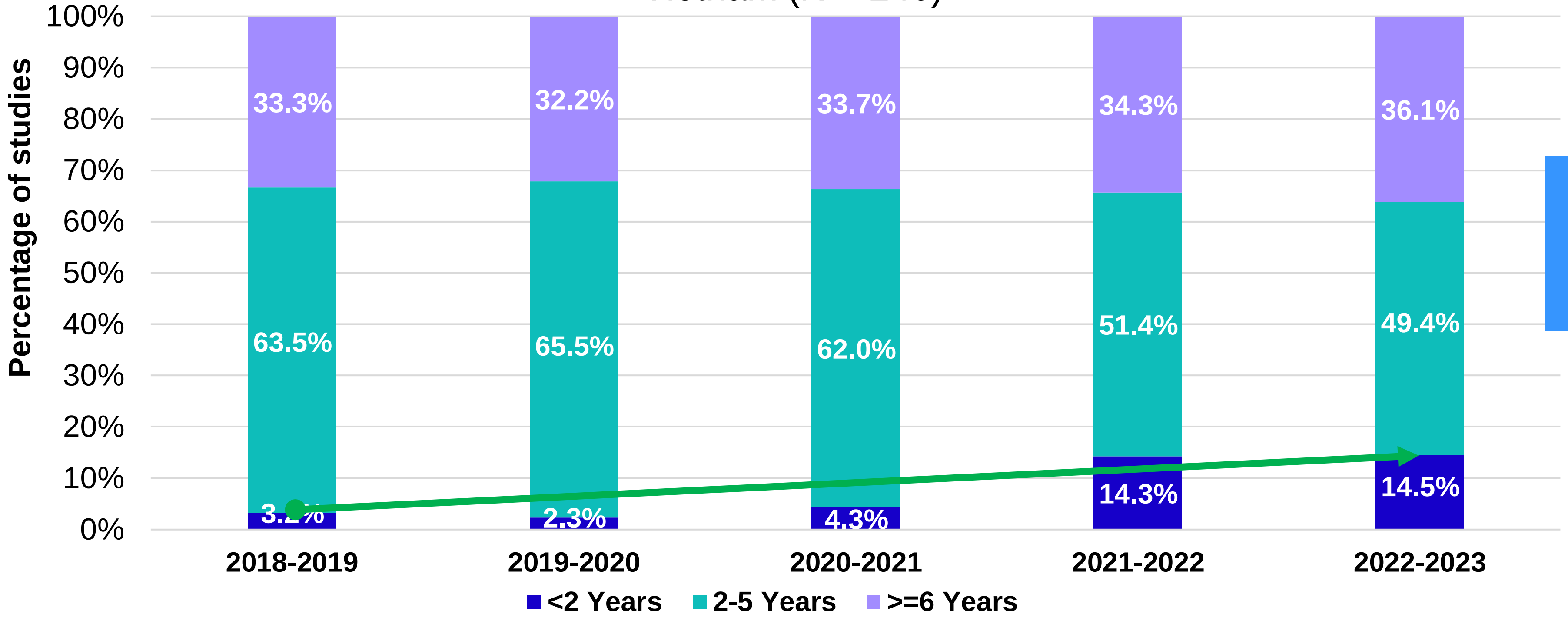

Rising trend in the number of studies being published within 2 years of their completion

*Out of 246 studies in target countries, 238 have a specified year of research completion, while 8 have an unspecified year of research completion.

^The lag period is defined as follows:
<2 Years (for publications published within 2 years after the research completion),
2-5 Years (for publications published within 2 to 5 years after the research completion), >=6 Years (for publications published 6 years and above after the research completion).

Trend for publication time lag from the integrated databases of cross-country collaborative studies from 2018-2023

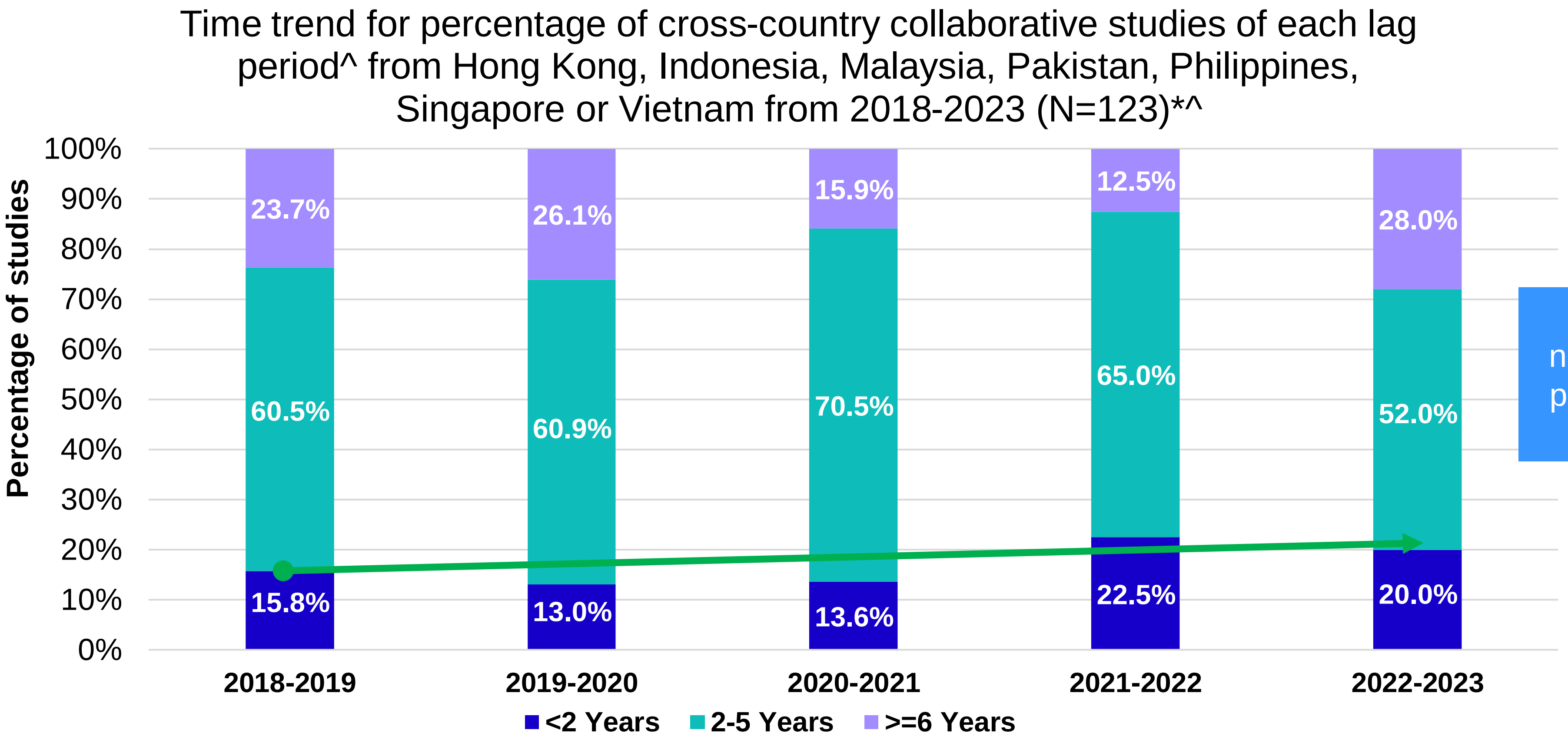

Rising trend in the number of studies being published within 2 years of their completion

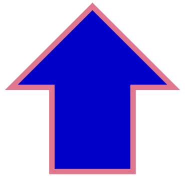

*Out of 123 studies in target countries, 107 have a specified year of research completion, while 16 have an unspecified year of research completion. Study numbers from cross-country studies may appear as duplicates for studies conducted in multiple target countries.

^The lag period is defined as follows:
<2 Years (for publications published within 2 years after the research completion),
2-5 Years (for publications published within 2 to 5 years after the research completion), >=6 Years (for publications published 6 years and above after the research completion).

Comparative analysis of publication speed for single-country studies from single exclusive databases

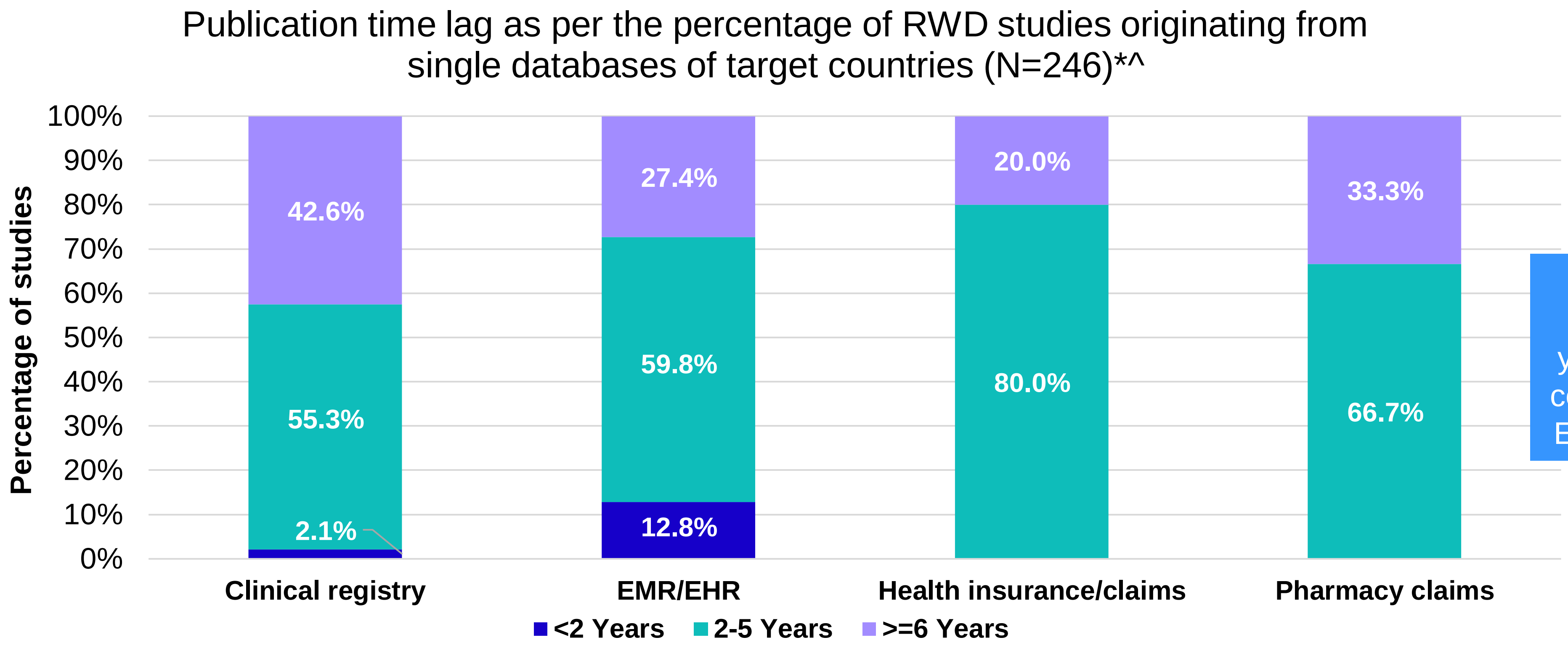

Majority of studies published within 2 years post research completion are using EMR/EHR database

*Out of 246 studies in target countries, 226 used a single database type/source, while 20 utilized a combination of one or more database types/sources.
Out of 226 studies with a single database type/source, 219 have a specified year of research completion, while 7 have an unspecified year of research completion.

^The lag period is defined as follows:
<2 Years (for publications published within 2 years after the research completion),
2-5 Years (for publications published within 2 to 5 years after the research completion), >=6 Years (for publications published 6 years and above after the research completion).

Abbreviations: EMR/HER, electronic medical record/electronic health record

Comparative analysis of publication speed for cross-country collaborative studies from single exclusive databases

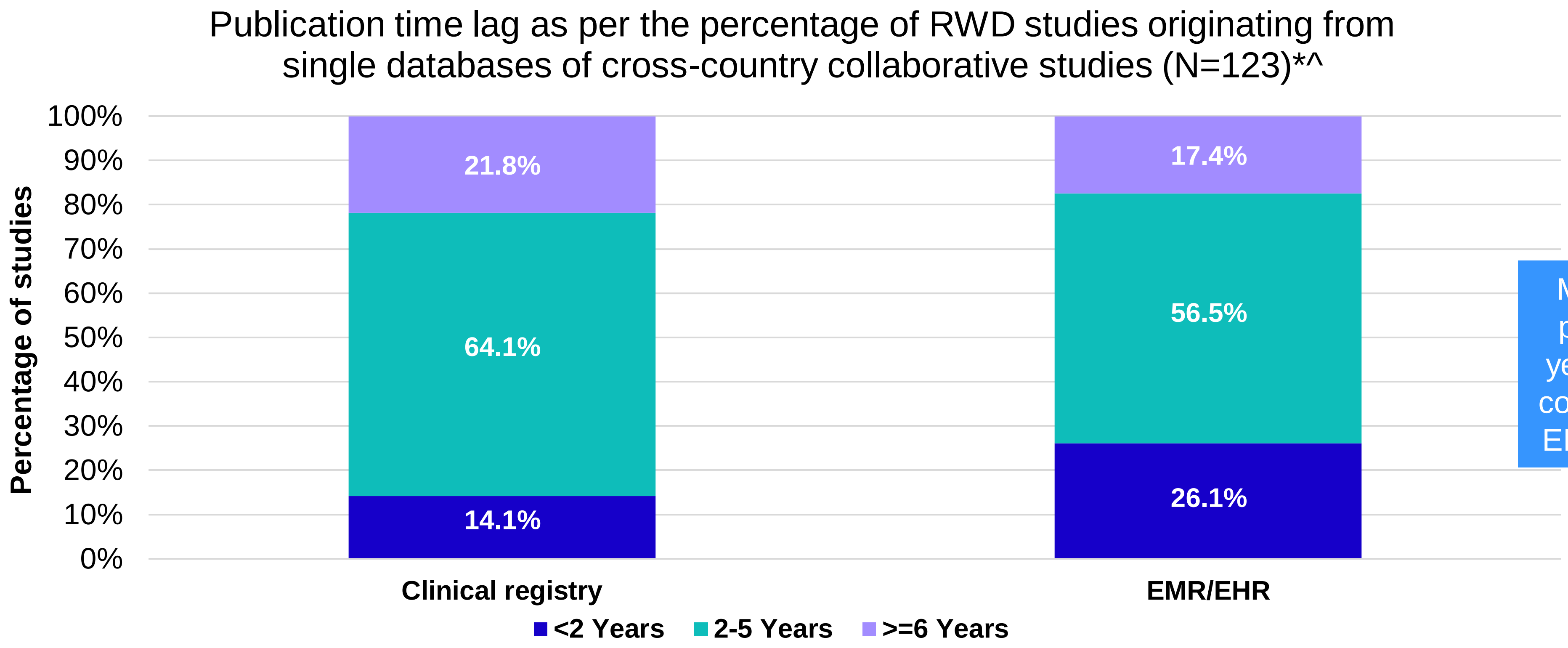

Majority of studies published within 2 years post research completion are using EMR/EHR database

*Out of 123 cross-country collaborative studies in target countries, 116 used a single database type/source, while 7 utilized a combination of one or more database types/sources. Out of 116 studies with a single database type/source, 102 have a specified year of research completion, while 14 have an unspecified year of research completion. No study is eligible for single exclusive health insurance/claims database.

^The lag period is defined as follows:
<2 Years (for publications published within 2 years after the research completion),
2-5 Years (for publications published within 2 to 5 years after the research completion), >=6 Years (for publications published 6 years and above after the research completion).

Abbreviations: EMR/HER, electronic medical record/electronic health record

Mean sample size from integrated database of target countries (SCS & CCCS combined)

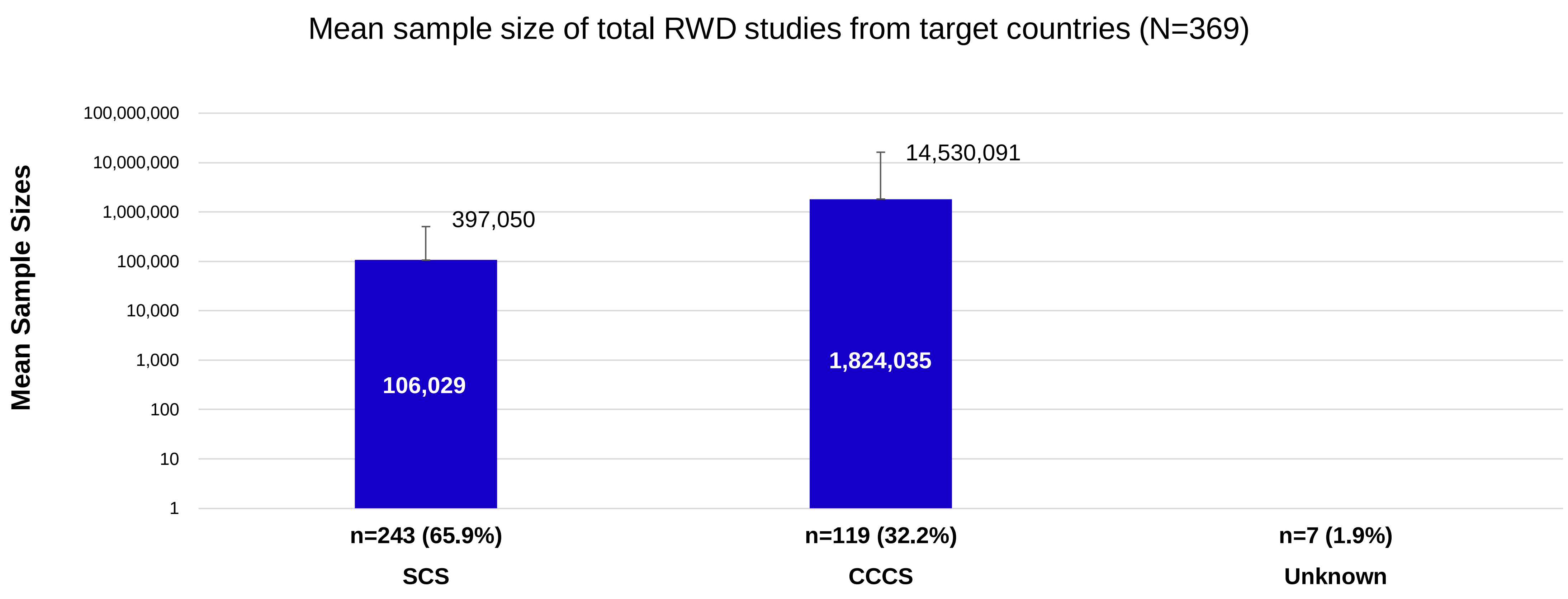

Abbreviations: RWD, real-world data; SCS, Single-country studies; CCCS, Cross-country collaborative studies

Sample size from integrated databases of seven target countries from 2018-2023

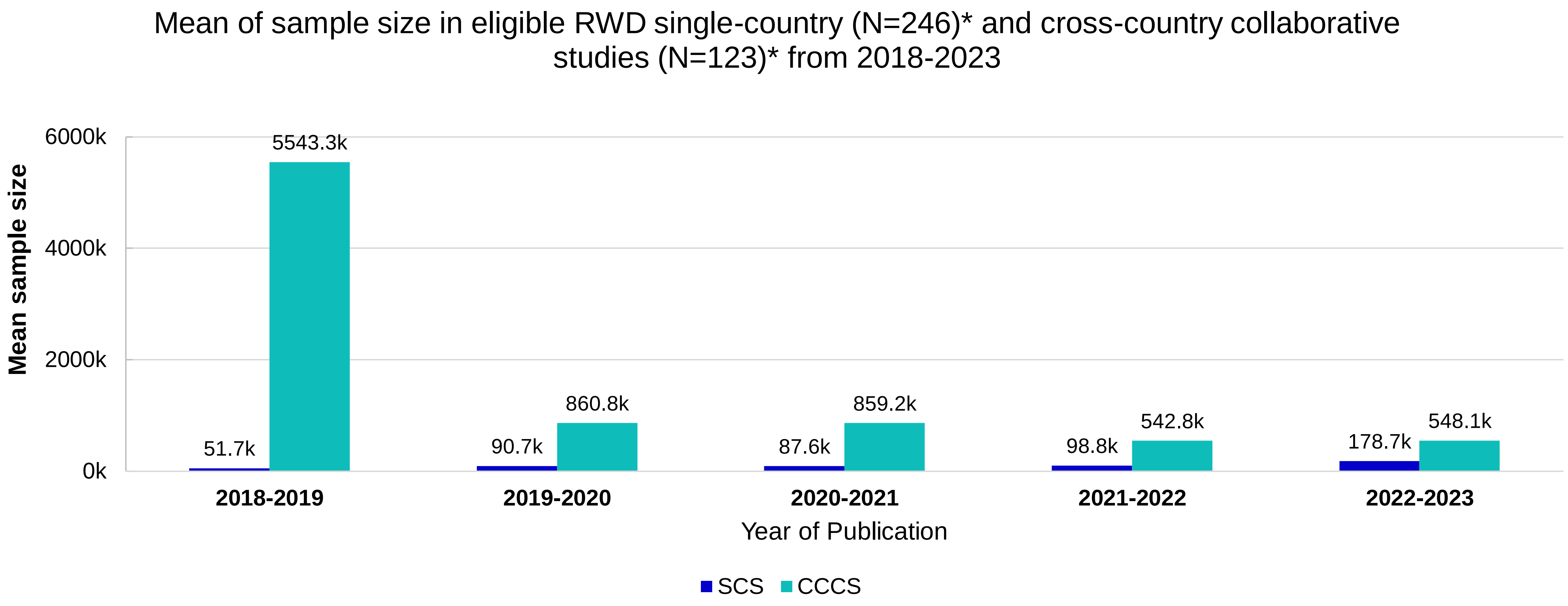

*As the PubMed final search was conducted on May 9, 2023, with filter for last 5 years, the eligible studies do not contain full data from 2018 and 2023. Study numbers from cross-country studies may appear as duplicates for studies conducted in multiple target countries. The moving average of sample size is calculated by averaging the mean of study duration in each consecutive year.

Abbreviations: k, thousand; RWD, real-world data; SCS, Single-country studies; CCCS, Cross-country collaborative studies

Study centers from integrated databases from 2018-2023

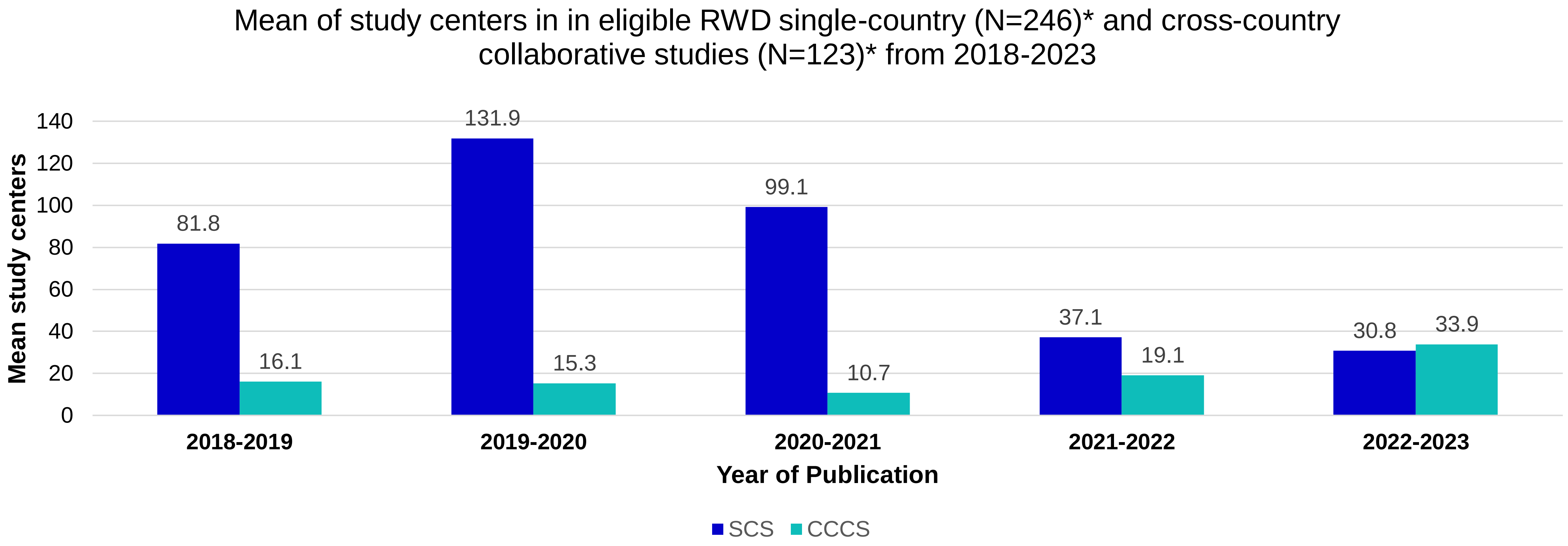

*As the PubMed final search was conducted on May 9, 2023, with filter for last 5 years, the eligible studies do not contain full data from 2018 and 2023. Study numbers from cross-country studies may appear as duplicates for studies conducted in multiple target countries. The moving average of sample size is calculated by averaging the mean of study duration in each consecutive year.

Abbreviations: RWD, real-world data; SCS, Single-country studies; CCCS, Cross-country collaborative studies
