# Supplementary material for: Integrated Real-World Data Warehouses Across 7 Evolving Asian Health Care Systems: Scoping Review
Source: J Med Internet Res. 2024 Jun 11;26:e56686. doi: 10.2196/56686 (PMC11200047; doi:10.2196/56686)
Supplement: Multimedia Appendix 3 [file jmir_v26i1e56686_app3.pdf]

Multimedia Appendix

Scoping review to identify and describe integrated contemporary real-world studies databases from three diverse healthcare systems in Asia: Hong Kong, Indonesia, Malaysia, Pakistan, Philippines, Singapore, and Vietnam

International Registered Report Identifier (IRRID): RR2-10.2196/43741

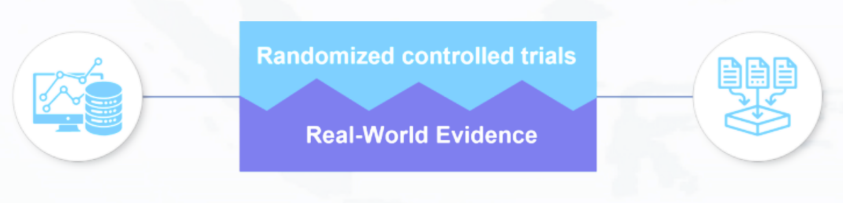

TOPLINE RESULTS

Real-world studies originating from contemporary integrated databases

Databases identified names from Hong Kong

Scoping review for Hong Kong, Indonesia, Malaysia, Pakistan, Philippines, Singapore and Vietnam

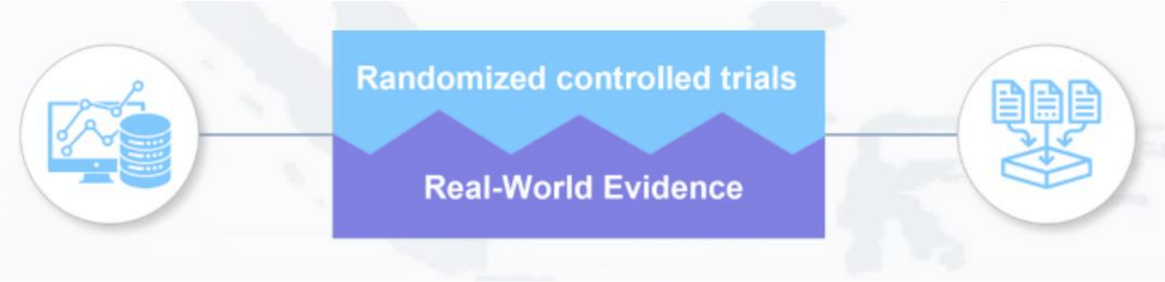

Names of identified databases from Hong Kong (1)

Identified clinical registry databases from Hong Kong (N=17)

Database Type	Database Name	Disease Area	Study Details
Clinical registry	Hong Kong Childhood Diabetes Registry	Cardiology and metabolic disorders	Tung 2020
	Hong Kong Diabetes Registry		Wang 2022
	Hong Kong Diabetes Registry		Lau 2019
	N/S		Zhang 2019
	Joint Asia Diabetes Evaluation (JADE)		Lim 2020
	Hong Kong TB-HIV Registry	Infectious diseases and vaccines	Chan 2018
	Public healthcare database		Lai FTT 2022
	N/S		Chan HLY 2019
	Hong Kong Biologics Registry	Inflammatory and autoimmune disorders	Mok 2023
	Hong Kong Cancer Registry	Oncology	Co 2020
	Hong Kong Cancer Registry		Chan 2018
	Hong Kong Cancer Registry		Ng 2019
	Digital Database of Thyroid Ultrasound Images (DDTI)	Others	Chambara 2021
	Fragility Fracture Registry		Chow 2018
	N/S		WongRMY 2021
	Hong Kong Cord Blood Bank Registry		Kwok 2018
	N/S		HungKCK 2022

Names of identified databases from Hong Kong (2)

Identified EMR/EHR databases of cardiology and metabolic disorders from Hong Kong (N=30)

Database Type	Database Name	Disease Area	Study Details
EMR/EHR	Clinical Data Analysis and Reporting System (CDARS)	Cardiology and metabolic disorders	Au PCM 2023
	Clinical Data Analysis and Reporting System (CDARS)		Au PCM 2022
	N/S		Au PCM 2022
	N/S		Cheung 2019
	N/S		Cheung 2021
	N/S		Law SWY 2018
	N/S		Zhou 2022
	N/S		Zhou 2023
	Clinical Data Analysis and Reporting System (CDARS)		Zhao 2020
	Clinical Data Analysis and Reporting System (CDARS)		Ju 2020
	N/S		Wu 2022
	Clinical Data Analysis and Reporting System (CDARS)		Yung NCL 2020
	Clinical Data Analysis and Reporting System (CDARS)		Blais 2022
	Clinical Data Analysis and Reporting System (CDARS)		Hsu WWQ 2022
	Clinical Data Analysis and Reporting System (CDARS)		Chen EYH 2023
	N/S		Lam 2020
	Electronic medical records from the Hong Kong Hospital Authority		Hong 2023
	Hong Kong Diabetes Surveillance Database or HKDSD (identified from Hong Kong Hospital Authority EMR system)		Wu 2020
	HKDSD		Wu 2020
	HKDSD		Wu 2020
	Hong Kong Hospital Authority EMR		Blais 2019
	Hong Kong Hospital Authority EMR		Lee 2021
	Hong Kong Hospital Authority EMR		Wan EYF 2023
	Hong Kong Osteoporosis Study cohort Clinical Data Analysis and Reporting System (CDARS)		Leung 2020
	N/S		Lee 2021
	N/S		Li 2020
	N/S		Lui DTW 2023
	N/S		Tenney 2020
	Risk Assessment and Management Programme for Diabetes Mellitus (RAMPDM); Hospital Authority EMR		Wu 2023
	Risk Assessment and Management Programme for Patients with Diabetes Mellitus (RAMP-DM)		Yang 2020

Names of identified databases from Hong Kong (3)

Identified EMR/EHR databases of other disease areas from Hong Kong (N=34)

Database Type	Database Name	Disease Area	Study Details
EMR/EHR	Hong Kong Hospital Authority EMR	Infectious diseases and vaccines	Cheng 2022
	Clinical Data Analysis and Reporting System (CDARS)		Liang 2022
	Clinical Data Analysis and Reporting System (CDARS)		WaiAKC 2022
	Clinical Data Analysis and Reporting System (CDARS)		Hui 2021
	N/S		Wong 2018
	N/S		Wong 2021
	Clinical Management System (CMS) by the Hospital Authority; Electronic Notification of Infectious Disease (eNID)		TsuiELH 2020
	Hong Kong Diabetes Surveillance Database (HKDSD)		LukAOY 2021
	Hospital Authority of the Department of Health		WongCKH 2022
	N/S		Chu 2022
	N/S		Du 2021
	N/S		Du 2022
	N/S		WongCKH 2022
	N/S		WongCKH 2022
	N/S		Xiong 2022
	Clinical Data Analysis Reporting System (CDARS)	Inflammatory and autoimmune disorders	Yu 2022
	N/S		WongSCT 2020
	N/S		WongSPY 2018
	Clinical Data Analysis and Reporting System (CDARS)	Oncology	Lee 2021
	Hong Kong Clinical Data Repository		Sung 2020
	Hospital Authority Clinical Data Repository		Li 2021
	N/S		Cheng 2019
	Clinical Analysis and Reporting System (CDARS)	Others	Wong 2023
	Clinical Data Analysis and Reporting System (CDARS)		Zhang 2022
	Clinical Data Analysis and Reporting System (CDARS)		Chu 2020
	N/S		Ge 2019
	Clinical Data Analysis and Reporting System (CDARS)		ManKKC 2020
	N/S		Chu 2022
	N/S		LamRPK 2020
	N/S		Lui 2018
	N/S		Rainer 2019
	N/S		Wang 2023
	N/S		Yeoh 2020
	Real-World Hip Fracture Cohort (RHFC) from the Clinical Data Analysis and Reporting System (CDARS)		HsuWWQ 2022

Names of identified databases from Hong Kong (4)

Identified EMR/HER; clinical registry databases from Hong Kong (N=5)

Database Type	Database Name	Disease Area	Study Details
EMR/EHR; Clinical registry	Hong Kong Diabetes Surveillance Database (HKDSD); Hong Kong Diabetes Register (HKDR)	Cardiology and metabolic disorders	LukAOY 2020
	Hong Kong Myositis Registry (MyoHK); Clinical Data Analysis and Reporting System (CDARS)	Inflammatory and autoimmune disorders	So 2022
	Hong Kong Myositis Registry; Clinical Data Analysis and Reporting System (CDARS)		So 2022
	Clinical Data Analysis and Reporting System (CDARS); Hong Kong Cancer Registry	Oncology	Leung 2018
	Child Protection Registry (CPR); Clinical Data Analysis and Report System (CDARS)	Others	Lo 2018

TOPLINE RESULTS

Real-world studies originating from contemporary integrated databases

Databases identified names from Indonesia

Scoping review for Hong Kong, Indonesia, Malaysia, Pakistan, Philippines, Singapore and Vietnam

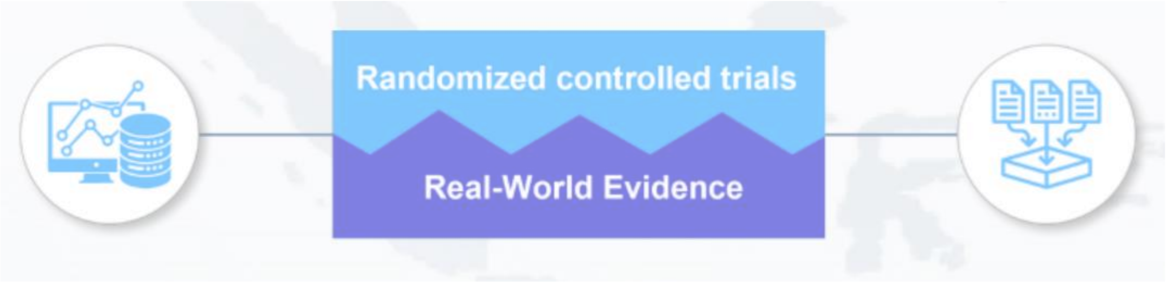

Names of identified databases from Indonesia

Identified clinical registry, EMR/HER, health insurance/claims databases from Indonesia (N=7)

Database Type	Database Name	Disease Area	Study Details
Clinical registry	Nationwide PCI registry	Cardiology and metabolic disorders	Alkatiri 2020
	Jakarta Province's COVID-19 epidemiological registry	Infectious diseases and vaccines	Harbuwono 2022
	Hasan Sadikin Lupus Registry	Inflammatory and autoimmune disorders	Hamijoyo 2022
	N/S	Oncology	Jasirwan COM 2020
EMR/EHR	N/S	Inflammatory and autoimmune disorders	Purba AKR 2020
	N/S	Others	Anggraini 2018
Health insurance/claims	Jaminan Kesehatan Nasional (database of Indonesia's National Health Insurance)	Infectious diseases and vaccines	Hidayat 2022

TOPLINE RESULTS

Real-world studies originating from contemporary integrated databases

Databases identified names from Malaysia

Scoping review for Hong Kong, Indonesia, Malaysia, Pakistan, Philippines, Singapore and Vietnam

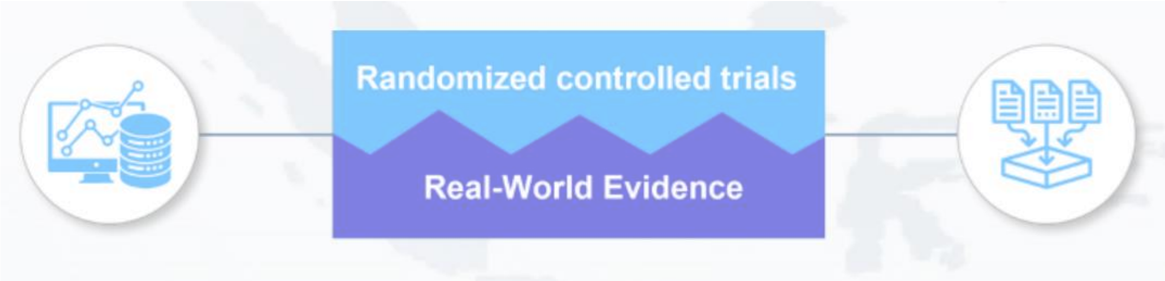

Names of identified databases from Malaysia (1)

Identified clinical registry databases from Malaysia (N=19)

Database Type	Database Name	Disease Area	Study Details
Clinical registry	Malaysian National Cardiovascular Database registry - acute coronary syndrome (NCVD-ACS); Malaysian Death Register	Cardiology and metabolic disorders	Venkatason 2019
	Malaysian National Cardiovascular Database-acute coronary syndrome) registry (NCVD-ACS)		Venkatason 2019
	Malaysian National Cardiovascular Disease Database - Percutaneous Coronary Intervention (NCVD-PCI) registry		Ismail 2018
	Malaysian National Cardiovascular Disease- ACS (NCVD-ACS) registry		Suki 2022
	National Cardiovascular Disease Database for Malaysia		Aziz 2021
	National Diabetes Registry		Wan 2021
	National Diabetes Registry		Wan 2020
	National Diabetes Registry.		Wan 2021
	National Diabetic Registry (NDR)		Rosedi 2022
	Pediatric Cardiology Clinical Information System		MatBah 2018
	The Malaysian National Cardiovascular Disease Database-Acute Coronary Syndrome (NCVD-ACS) registry		Lee 2021
	Tuberculosis Information System (TBIS)	Infectious diseases and vaccines	LaiJML 2019
	Malaysian national dengue passive surveillance system, e-Dengue registry		Woon 2019
	Perlis Rabid Potential Animal Bite Registry		MohammadBasir 2023
	National TB surveillance database		Tok PSK 2020
	Johor Kawasaki Clinical Registry	Inflammatory and autoimmune disorders	MatBah 2022
	Kawasaki Disease Information System		MatBah 2021
	Malaysian National Neonatal Registry (MNNR)		Boo 2022
	Malaysian Psoriasis Registry (MPR)		Gan 2023
	N/S		Robinson 2023

Names of identified databases from Malaysia (2)

Identified clinical registry databases from Malaysia (N=18)

Database Type	Database Name	Disease Area	Study Details
Clinical registry	Kelantan Cancer Registry	Oncology	Hanis 2021
	Malaysia Lung Cancer Registry		How 2022
	Malaysian Lung Cancer Registry		How 2022
	Malaysian National Cancer Patient Registry-Colorectal Cancer (NCPR-CRC)		Ghazali 2021
	Malaysian National Cancer Registry		NikAbKadir 2021
	Malaysian National Cancer Registry (MNCR)		Amir 2022
	N/S		NikAbKadir 2022
	N/S		NikAbKadir 2022
			Poh 2023
	Cataract Surgery Registry (CSR) in the National Eye Database (NED)	Others	Soundarajan 2021
	Kelantan Cancer Registry		Hanis 2019
	Malaysia National Stroke Registry		Chen 2019
	Malaysian Cataract Surgery Registry (CSR)		Lim 2021
	Malaysian Ministry of Health Cataract Surgery Registry (MOH CSR)		Yong 2022
	Malaysian National Neonatal Registry		Boo 2021
	Malaysian National Neurology Registry		Albitar 2020
	N/S		Aziz 2019
	Stop Smoking Services (SSS) registry		Ikhwan 2022

Names of identified databases from Malaysia (3)

Identified EMR/EHR, EMR/HER; Clinical registry, and pharmacy claims databases from Malaysia (N=13)

Database Type	Database Name	Disease Area	Study Details
EMR/EHR	Health Informatics Centre, Ministry of Health, Malaysia	Cardiology and metabolic disorders	LimYMF 2022
	N/S		Azit 2021
	N/S	Infectious diseases and vaccines	Ismail 2022
	N/S		Mardhiah 2021
	N/S	Inflammatory and autoimmune disorders	Patel 2022
	National Cardiovascular and Thoracic Surgical Database (NCTSD)	Oncology	Poh 2023
	National ADR reporting database	Others	Panickar 2020
	N/S		Musa 2018
	National ADR reporting database		Lee 2020
EMR/EHR; Clinical registry	SAFECOVAC study, national COVID-19 vaccination register	Infectious diseases and vaccines	AbRahman 2022
	Malaysia Prostate Cancer (M-CaP) database	Oncology	Lim 2021
Pharmacy claims	N/S	Others	Doris 2019
	N/S		Zin 2019

TOPLINE RESULTS

Real-world studies originating from contemporary integrated databases

Databases identified names from Pakistan

Scoping review for Hong Kong, Indonesia, Malaysia, Pakistan, Philippines, Singapore and Vietnam

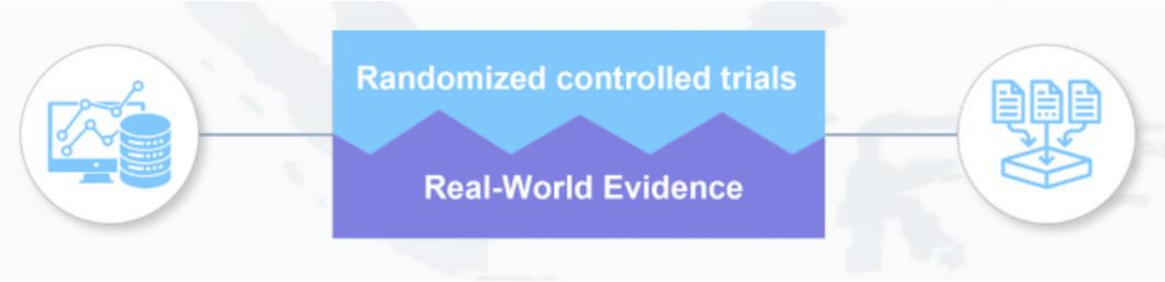

Names of identified databases from Pakistan

Identified clinical registry, EMR/EHR, and pharmacy claims databases from Pakistan (N=13)

Database Type	Database Name	Disease Area	Study Details
Clinical registry	CROP (Cardiac Registry of Pakistan) CathPCI database	Cardiology and metabolic disorders	Peerwani 2023
	Punjab Cancer Registry (PCR)	Oncology	Badar 2020
	Transfusion-dependent thalassaemia quality improvement (TDT QI) registry	Others	Hoodbhoy 2020
EMR/EHR	N/S	Cardiology and metabolic disorders	Kamin 2020
	N/S	Infectious diseases and vaccines	Iftikhar 2019
	N/S		Javaid 2021
	N/S		Khan 2022
	N/S		Sarwar 2018
	N/S	Oncology	Baig 2022
	N/S		Bajwa 2022
	N/S		Enam 2022
	N/S		Khalid 2022
Pharmacy claims	N/S	Others	Khan 2020

TOPLINE RESULTS

Real-world studies originating from contemporary integrated databases

Databases identified names from Philippines

Scoping review for Hong Kong, Indonesia, Malaysia, Pakistan, Philippines, Singapore and Vietnam

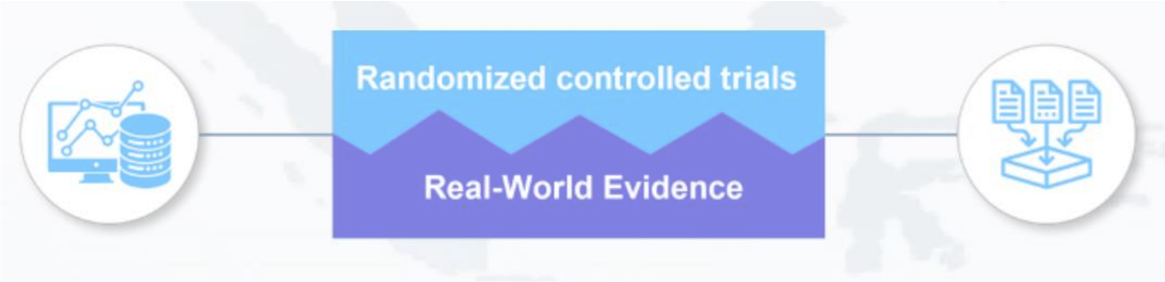

Names of identified databases from Philippines

Identified clinical registry and EMR/HER databases from Philippines (N=3)

Database Type	Database Name	Disease Area	Study Details
Clinical registry	Lysosomal Storage Disease (LSD) registry	Cardiology and metabolic disorders	Racoma MJC 2021
	Philippine Renal Disease Registry	Others	Bayani DBS 2021
EMR/EHR	N/S	Infectious diseases and vaccines	Cheng KJG 2020

TOPLINE RESULTS

Real-world studies originating from contemporary integrated databases

Databases identified names from Singapore

Scoping review for Hong Kong, Indonesia, Malaysia, Pakistan, Philippines, Singapore and Vietnam

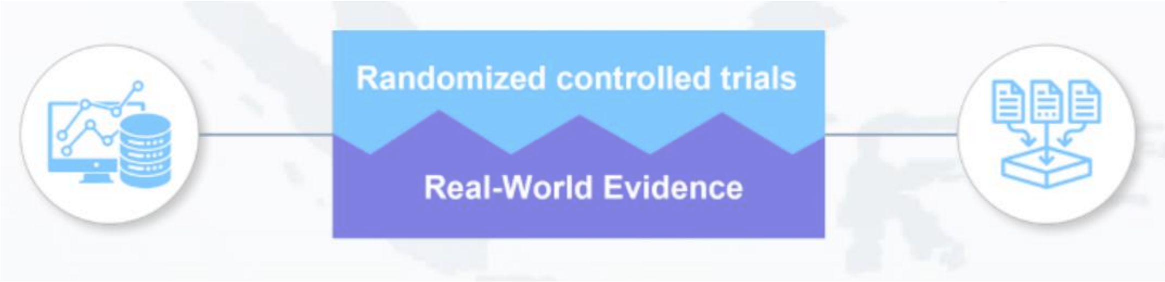

Names of identified databases from Singapore (1)

Identified clinical registry databases of cardiology and metabolic disorders from Singapore (N=22)

Database Type	Database Name	Disease Area	Study Details
Clinical registry	A Singapore VenaSealTM real world post-market evaluation Study (ASVS) registry	Cardiology and metabolic disorders	Tang 2021
	Microwave Ablations for Treatment of Varicose Veins in Singapore (MAESTRO) registry		Tang 2022
	N/S		Kojodjojo 2023
	N/S		Pan 2019
	Pan Asian Resuscitation Outcomes Study (PAROS) registry		Chia MYC 2019
	Pan-Asian Resuscitation Outcomes Study (PAROS) registry		Ho 2020
	Pan-Asian Resuscitation Outcomes Study (PAROS) registry		Ho AFW 2023
	N/S		Liu 2022
	Pan-Asian Resuscitation Outcomes Study (PAROS) registry		Ho AFW 2022
	Prospective Electrophysiological Ablation and Cardiac implantable Electronic device registry		Tan ESJ 2023
	Singapore Health Services (SingHealth) Diabetes Registry		Sun 2021
	Singapore Myocardial Infarction Registry (SMIR)		Zheng 2019
	Singapore Myocardial Infarction Registry (SMIR)		Bulluck 2019
	N/S		Cai 2020
	N/S		Ho AFW 2019
	N/S		Pong 2019
	N/S		Sia 2020
	Singapore Myocardial Infarction Registry (SMIR)		Paradies 2020
	N/S		Sia 2021
	N/S		Sia 2021
	Singapore Myocardial Infarction Registry (SMIR); Registry of Births and Deaths		Sia 2022
	Singapore's Pan-Asian Resuscitation Outcomes Study registry		Chual SY 2022

Names of identified databases from Singapore (2)

Identified clinical registry databases of other disease areas from Singapore (N=12)

Database Type	Database Name	Disease Area	Study Details
Clinical registry	National Immunization Registry of Singapore	Infectious diseases and vaccines	Loy 2020
	National HIV Registry; Registry of Births and Deaths		HoZJM 2019
	Singapore national TB registry		Chua 2018
	N/S	Oncology	Tan 2021
	N/S	Others	Chua 2022
	N/S		Chua 2023
	National Birth Defects Registry; Singapore Registry of Births and Deaths		Yow 2021
	National Dental Centre Singapore (NDCS) Endodontics Registry		Phang 2020
	Singapore National Stroke Registry; National Trauma Registry		Wei 2019
	Singapore National Trauma Registry		Tan 2018
	Singapore Stroke Registry		Xu 2020
	Singapore Stroke Registry; National Death Registry		Xu 2021

Names of identified databases from Singapore (3)

Identified clinical registry; health insurance/claims and EMR/EHR databases from Singapore (N=16)

Database Type	Database Name	Disease Area	Study Details
Clinical registry; Health insurance/claims	Nationwide claims data; National Death Registry	Others	Yong 2020
EMR/EHR	Electronic training and medical records of the Singapore Armed Forces	Cardiology and metabolic disorders	Gorny 2023
	N/S		DeLeon 2022
	National Healthcare Group (NHG) Chronic Disease Management System (CDMS)		Riandini 2021
	Pan-Asian Resuscitation Outcomes Study (PAROS)		Pek 2019
	Singapore Cardiovascular Longitudinal Outcomes Database (SingCLOUD); Singapore Cardiac Data Bank		Yeo 2019
	Pan-Asian Resuscitation Outcomes Study (PAROS)		Tay PJM 2020
	National electronic health record database	Infectious diseases and vaccines	Tan 2023
	National University Health System (NUHS)		Ko 2023
	Singapore Armed Forces EMR	Inflammatory and autoimmune disorders	Kok 2019
	Breast cancer database of KK Women's and Children's Hospital (KKH), Singapore General Hospital (SGH) and National Cancer Centre Singapore (NCCS)	Oncology	Tan 2023
	Lymphoma databases from the National Cancer Centre Singapore (NCCS), National University Hospital, Singapore (NUHS) and Singapore General Hospital (SGH		Tay 2022
	N/S		Chan 2020
	N/S		Rajasooriyar 2019
	N/S		Tay 2021
	N/S		Lo 2021

Names of identified databases from Singapore (4)

Identified EMR/EHR databases from Singapore (N=16)

Database Type	Database Name	Disease Area	Study Details
EMR/EHR	N/S	Others	ChanJSE 2021
	N/S		Chia 2018
	N/S		Ko 2022
	N/S		Lim 2021
	N/S		QuakXES 2022
	N/S		Zhu 2018
	National advance care planning (ACP) information technology system; electronic medical records of individual hospitals; administrative database from the Ministry of Health		Tan 2018
	National Healthcare Group (NHG) Chronic Disease Management System (CDMS)		Yeo 2020
	National Healthcare Group Polyclinics (NHGP) administrative database		Tan 2021
	National inpatient admission data from the Ministry of Health (MOH)		Riandini 2020
	National Neuroscience Institute's Parkinson's Disease and Movement Disorder (PDMD) database; administrative medical claims and subvention dataset from the Ministry of Health		SohEML 2022
	Integrated Population Health Management (PHM) database		AngIYH 2019
	Singapore data from the Pan-Asian Resuscitation Outcomes Study (PAROS)		Pourghaderi 2022
	Singapore Eastern Regional Health System (RHS)		Yan 2019
	Singapore KneE OA CoHort (SKETCH) study		Tan 2023
	SingHealth Electronic Health Records (SingHealth EHRs)		Low 2019

Names of identified databases from Singapore (5)

Identified EMR/HER; clinical registry, EMR/HER; clinical registry; health insurance/claims, EMR/HER; pharmacy claims, and health insurance/claims databases from Singapore (N=14)

Database Type	Database Name	Disease Area	Study Details
EMR/EHR; Clinical registry	Singapore Study of Macro-angiopathy and Micro-vascular Reactivity in Type 2 Diabetes (SMART2D) and Diabetic Nephropathy (DN) cohorts, Singapore Myocardial Infarction Registry	Cardiology and metabolic disorders	Gurung 2019
	SingHealth Diabetes Registry		Feng 2021
	National Immunisation Register	Infectious diseases and vaccines	Ma 2023
	National Skin Centre electronic medical and histology records, National Registry of Diseases Office (NRDO) death registry	Inflammatory and autoimmune disorders	Cai 2020
	National AMI registry; national death registry	Oncology	Lee 2019
	National AMI registry; national death registry		Lee 2018
	National death registry		Soon 2018
	Singapore Childhood Cancer Registry (SCCR)		Othman 2021
	National Healthcare Group (NHG) Chronic Disease Management System (CDMS); Singapore Stroke Registry (SSR)	Others	Yeo 2020
	Singapore Renal Registry		Geng 2019
EMR/EHR; Clinical registry; Health insurance/claims	Singapore Myocardial Infarct Registry (SMIR); Singapore Cardiac Databank PCI registry; Ministry of Health Mediclaims database	Cardiology and metabolic disorders	Loh 2018
EMR/EHR; Pharmacy claims	INSIDER study; SingHealth Diabetes Registry	Cardiology and metabolic disorders	Lim 2022
Health insurance/claims	Ministry of Health (MOH)'s administrative database	Cardiology and metabolic disorders	GohLGH 2022
	Ministry of Health (MOH) Central Claims Processing System	Infectious diseases and vaccines	Goh 2020

TOPLINE RESULTS

Real-world studies originating from contemporary integrated databases

Databases identified names from Vietnam

Scoping review for Hong Kong, Indonesia, Malaysia, Pakistan, Philippines, Singapore and Vietnam

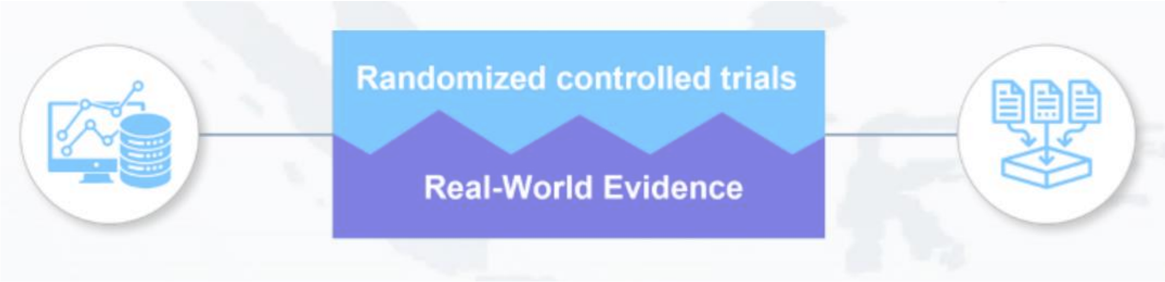

Names of identified databases from Vietnam

Identified clinical registry, EMR/EHR, health insurance/claims databases from Vietnam (N=7)

Database Type	Database Name	Disease Area	Study Details
Clinical registry	Ho Chi Minh City Cancer Registry	Oncology	Pham 2021
EMR/EHR	N/S	Inflammatory and autoimmune disorders	Do 2022
	N/S		Do 2023
	N/S		Nguyen 2019
	N/S	Others	DaoATM 2018
Health insurance/claims	Vietnam Health Insurance Scheme (VHIS) database	Cardiology and metabolic disorders	Bui 2020
	Vietnam Health Insurance Scheme (VHIS) database		TuanKietPham 2020

TOPLINE RESULTS

Real-world studies originating from contemporary integrated databases

Databases identified names from cross-country collaboration studies

Scoping review for Hong Kong, Indonesia, Malaysia, Pakistan, Philippines, Singapore and Vietnam

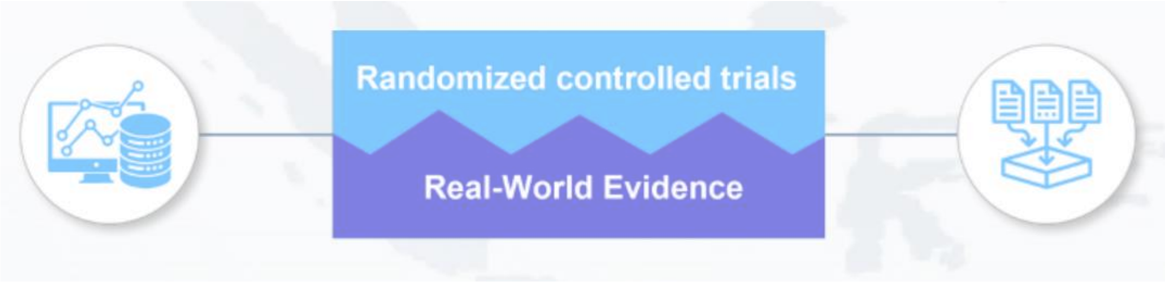

Names of identified databases (1)

Identified EMR/EHR; clinical registry and clinical registry databases from cross-country collaboration studies (N=5)

Target countries	Database Type	Database Name	Disease Area	Study Details
Hong Kong; Indonesia; Pakistan; One/more non-target countries	EMR/EHR; Clinical registry	N/S	Infectious diseases and vaccines	Smith 2022
Hong Kong; Malaysia; Indonesia; One/more non-target countries	Clinical registry	Registry on WATCHMAN Outcomes in Real-Life Utilization (WASP)	Cardiology and metabolic disorders	Phillips 2019
Hong Kong; Malaysia; One/more non-target countries	Clinical registry	Reallife experience of ADT in Asia (READT) registry	Oncology	WongCHM 2023
Hong Kong; Malaysia; Pakistan; One/more non-target countries	Clinical registry	Global Non-Alcoholic Steatohepatitis (NASH)/ NonAlcoholic Fatty Liver Disease (NAFLD) Registry	Cardiology and metabolic disorders	Younossi 2022
Hong Kong; Malaysia; Vietnam; One/more non-target countries	Clinical registry	EPICOR (long-tErm follow-uP of anti-thrombotic management patterns In acute CORonary syndrome patients) Asia registry	Cardiology and metabolic disorders	Zhang 2020

Names of identified databases (2)

Identified clinical registry, EMR/EHR and EMR/EHR; pharmacy claims databases from Hong Kong; One/more non-target countries (N=17)

Target countries	Database Type	Database Name	Disease Area	Study Details
Hong Kong; One/more non-target countries	Clinical registry	Identification of Predictors for Coronary Plaque Erosion in Patients with Acute Coronary Syndrome study	Cardiology and metabolic disorders	Kurihara 2020
		N/S	Infectious diseases and vaccines	Hsu 2021
		REAL-C registry		Huang 2019
		N/S	Inflammatory and autoimmune disorders	Co 2018
		International Agency for Research on Cancer/World Health Organization mortality database	Oncology	Abdel-Rahman 2018
		Real-World Evidence from the Asia Liver Consortium (REAL-C) registry		Ogawa 2020
		Robot-assisted Minimally Invasive Esophagectomy (RAMIE) registry by the Upper GI International Robotic Association (UGIRA)		Kingma 2022
		Sun Yat-sen University Cancer Center and Prince of Wales Hospital (SYSUCC-PWH) cohort; Surveillance, epidemiology, and end results (SEER) database		He 2020
		Treatment of Recurrent and Advanced Colorectal Cancer (TRACC) registry		Burge 2018
		N/S		Travers 2021
		Treatment Patterns in Patients With Metastatic Castration-Resistant Prostate Cancer Previously Treated With Docetaxel-Based Chemotherapy (PROXIMA)		Akaza 2018
		TraumaRegister DGU® and trauma registries from Prince of Wales Hospital (PWH), Princess Margaret Hospital (PMH), Queen Elizabeth Hospital (QEH), Queen Mary Hospital (QMH) and Tuen Mun Hospital (TMH)	Others	Lai 2021
	EMR/EHR	Identification of Predictors for Coronary Plaque Erosion in Patients with Acute Coronary Syndrome study	Cardiology and metabolic disorders	Yamamoto 2019
		N/S		Petta 2021
		N/S	Oncology	Kim 2020
		N/S	Others	Raman 2018
	EMR/ EHR; Pharmacy claims	Clinformatics Data Mart database (OptumInsight)	Cardiology and metabolic disorders	Wang 2019

Names of identified databases (3)

Identified clinical registry databases from cross-country collaboration studies (N=7)

Target countries	Database Type	Database Name	Disease Area	Study Details
Hong Kong; Singapore	Clinical registry	N/S	Oncology	HungriaVTM 2019
Hong Kong; Singapore; Indonesia; One/more non-target countries	Clinical registry	N/S	Cardiology and metabolic disorders	DeLuca 2022
Hong Kong; Singapore; Malaysia; Indonesia; One/more non-target countries	Clinical registry	GOASIA liver registry	Cardiology and metabolic disorders	Tan 2022
Hong Kong; Singapore; Malaysia; Indonesia; Philippines; One/more non-target countries	Clinical registry	ASIAN-HF (Asian Sudden Cardiac Death in Heart Failure) Registry	Cardiology and metabolic disorders	Yap 2019
		ASIAN-HF registry		Teng 2018
		HF-ACTION trial; ASIAN-HF registry		Cooper 2018
	Clinical registry	ASIAN-HF registry	Cardiology and metabolic disorders	MacDonald 2020

Names of identified databases (4)

Identified clinical registry, EMR/EHR; clinical registry, and EMR/EHR databases from cross-country collaboration studies (N=6)

Target countries	Database Type	Database Name	Disease Area	Study Details
Hong Kong; Singapore; Malaysia; Indonesia; Philippines; Vietnam; One/more non-target countries	Clinical registry	Pan-Asia Trauma Outcomes Study (PATOS) registry	Others	Chen 2022
Hong Kong; Singapore; Malaysia; Indonesia; Philippines; Vietnam; Pakistan; One/more non-target countries	EMR/EHR; Clinical registry	Global Burden of Disease Study	Oncology	Fitzmaurice 2019
Hong Kong; Singapore; Malaysia; Indonesia; Vietnam; One/more non-target countries	Clinical registry	The Asia Pacific Evaluation of Cardiovascular Therapies (ASPECT) collaboration	Cardiology and metabolic disorders	WongMYZ 2023
Hong Kong; Singapore; Malaysia; One/more non-target countries	Clinical registry	BIOSOLVE-IV-registry	Cardiology and metabolic disorders	Verheye 2021
Hong Kong; Singapore; Malaysia; Vietnam; One/more non-target countries	Clinical registry	Asia Pacific Evaluation of Cardiovascular Therapies (ASPECT) registry	Cardiology and metabolic disorders	Reid 2023
	EMR/EHR	EPICOR (long-tErm follow-uP of antithrombotic management patterns In acute CORonary syndrome patients); EPICOR Asia	Cardiology and metabolic disorders	Rossello 2020

Names of identified databases (5)

Identified clinical registry, EMR/EHR, and EMR/EHR; clinical registry; health insurance/claims databases from cross-country collaboration studies (N=17)

Target countries	Database Type	Database Name	Disease Area	Study Details
Hong Kong; Singapore; One/more non-target countries	Clinical registry	CTO (chronic total occlusion) registry	Cardiology and metabolic disorders	Wu 2020
		N/S		Chan 2019
		Asian Robot-Assisted Radical Cystectomy (RARC) registry	Oncology	Teoh 2021
	EMR/EHR	American Joint Committee on Cancer - Ophthalmic Oncology Task Force's (AJCC-OOTF) Retinoblastoma Registry	Oncology	Tomar 2021
		N/S	Others	Sing 2021
	EMR/EHR; Clinical registry; Health insurance/claims	N/S	Cardiology and metabolic disorders	Magliano 2022
Hong Kong; Singapore; Pakistan; One/more non-target countries	Clinical registry	SpyGlass AMEA Registry	Others	Maydeo 2019
Hong Kong; Singapore; Philippines; One/more non-target countries	Clinical registry	SpyGlass AMEA Registry	Others	Almadi 2020
Hong Kong; Singapore; Vietnam; One/more non-target countries	EMR/EHR	N/S	Others	ChongCC 2022
Indonesia; One/more non-target countries	Clinical registry	FOCUS Registry	Cardiology and metabolic disorders	Qu 2019
Indonesia; Vietnam; Pakistan; One/more non-target countries	EMR/EHR	EndTB observational study	Infectious diseases and vaccines	Khan 2019
		N/S		Nguyen 2021
Malaysia; Indonesia; Philippines; One/more non-target countries	Clinical registry	ESMO COVID-19 and CAncer REgistry (ESMO-CoCARE)	Oncology	Castelo-Branco 2022
Malaysia; Indonesia; Philippines; Pakistan; One/more non-target countries	EMR/EHR	ISARIC (International Severe Acute Respiratory and Emerging Infections Consortium) COVID-19 database	Infectious diseases and vaccines	Gonçalves 2022
Malaysia; Indonesia; Philippines; Vietnam	EMR/EHR	REPORT-HF study	Cardiology and metabolic disorders	Filippatos 2020
Malaysia; Indonesia; Vietnam; One/more non-target countries	Clinical registry	Pan-Asian Trauma Outcome Study (PATOS) registry	Others	Wang 2023
	EMR/EHR	International Epidemiology Databases to Evaluate AIDS (IeDEA)	Infectious diseases and vaccines	Wools-Kaloustian 2018

Names of identified databases (6)

Identified clinical registry, EMR/EHR; clinical registry, and EMR/EHR databases from cross-country collaboration studies (N=17)

Target countries	Database Type	Database Name	Disease Area	Study Details
Malaysia; One/more non-target countries	Clinical registry	BIOLUX P-III	Cardiology and metabolic disorders	Mwipatayi 2021
		ISAR 2000 all-comers registry		Krackhardt 2018
		Safety and effectiveness of the self-aPposing, bAlloon-delivered, siRolimus-eluting stent for the Treatment of the CAD (SPARTA)		Montefusco 2020
		The DCB-only All-Comers Registry		Rosenberg 2019
		WHO aDSM (active TB drug safety monitoring and management)	Infectious diseases and vaccines	Akkerman 2019
		Pan-Asia Trauma Outcomes Study (PATOS) database	Others	Chen 2020
		Pan-Asian Trauma Outcomes Study (PATOS)		Kim 2021
	EMR/EHR	TriNetX Analytics Network; US Collaborative Network	Infectious diseases and vaccines	Taquet 2022
Malaysia; Pakistan; One/more non-target countries	Clinical registry	Global Liver Registry (GLR)	Infectious diseases and vaccines	Younossi 2023
		Global Liver Registry (GLR)		Younossi 2022
Malaysia; Philippines; Vietnam; One/more non-target countries	Clinical registry	Pan-Asian Trauma Outcome Study (PATOS) registry	Others	Hsieh 2022
Malaysia; Vietnam; Pakistan; One/more non-target countries	Clinical registry	International Quality Improvement Collaborative Congenital Heart Disease Catheterization Registry (IQIC-CHDCR)	Cardiology and metabolic disorders	Barry 2021
Pakistan; One/more non-target countries	Clinical registry	N/S	Cardiology and metabolic disorders	Power 2022
	EMR/EHR	Shaukat Khanum Memorial Cancer Hospital and Research Centres (SKMCH&RC) COVID-19 database	Infectious diseases and vaccines	JuniorEPP 2023
	EMR/EHR; Clinical registry	N/S	Infectious diseases and vaccines	Morgan 2022
Singapore; Malaysia; Indonesia; One/more non-target countries	Clinical registry	RE-LATE	Cardiology and metabolic disorders	Choi 2020
	EMR/EHR; Clinical registry	Population-based cancer registries (PBCRs) in Lampang (Thailand), Penang (Malaysia) and Yogyakarta (Indonesia); Lung Cancer Consortium Singapore National Lung Cancer Research Study; Singapore Cancer Registry	Oncology	Soo 2022

Names of identified databases (7)

Identified clinical registry, EMR/EHR; clinical registry, and EMR/EHR from cross-country collaboration studies (N=16)

Target countries	Database Type	Database Name	Disease Area	Study Details
Singapore; Malaysia; Indonesia; Pakistan	Clinical registry	Pediatric Acute and Critical Care COVID-19 Registry of Asia (PACCOVRA)	Infectious diseases and vaccines	WongJJM 2022
Singapore; Malaysia; Indonesia; Philippines; One/more non-target countries	EMR/EHR	DISCOVER Global Registry	Cardiology and metabolic disorders	Khunti 2021
		N/S	Inflammatory and autoimmune disorders	Techatraisak 2019
Singapore; Malaysia; Indonesia; Philippines; Vietnam; Pakistan; One/more non-target countries	EMR/EHR	Global Burden of Disease Study	Cardiology and metabolic disorders	Yadgir 2020
	EMR/EHR; Clinical registry	Global Burden of Diseases, Injuries, and Risk Factors Study (GBD) 2017	Inflammatory and autoimmune disorders	GBD 2017 Inflammatory Bowel Disease Collaborators
Singapore; Malaysia; One/more non-target countries	Clinical registry	Cryo AF Global Registry	Cardiology and metabolic disorders	Rordorf 2021
		Pan-Asian Resuscitation Outcomes Study (PAROS) registry		Liu 2020
		Registry Identifier: NOPRODPCR40	Oncology	Liu 2019
		United in Fight against prOstate cancer (UFO) registry		Uemura 2020
		BIOLUX P-III	Others	Brodmann 2020
		BIOLUX P-III Passeo-18 Lux All-Comers Registry		Tepe 2021
Singapore; Malaysia; Pakistan; One/more non-target countries	Clinical registry	International Globe and Adnexal Trauma Epidemiology Study (IGATES) platform	Others	Hoskin 2021
Singapore; Malaysia; Philippines; One/more non-target countries	Clinical registry	Cryo AF Global Registry	Cardiology and metabolic disorders	ChunKRJ 2021
		International Pediatric HD Network (IPHN) Registry	Others	Borzych-Duzalka 2019
Singapore; Malaysia; Vietnam; One/more non-target countries	Clinical registry	prospeCtive observational LongitudinAl RegIstry oF patients with stable coronary arterY disease (CLARIFY) registry	Cardiology and metabolic disorders	Mak 2022
	EMR/EHR	Observational Prospective Study in Management of 1st-Line mCRC With Erbitux (cetuximab) (OPTIM1SE)	Oncology	Yang 2023

Names of identified databases (8)

Identified clinical registry databases from Singapore; One/more non-target countries (N=12)

Target countries	Database Type	Database Name	Disease Area	Study Details
Singapore; One/more non-target countries	Clinical registry	GARFIELD-AF Registry	Cardiology and metabolic disorders	Goto 2019
		Lutonix AV Global Registry		Karnabatidis 2021
		N/S		Amanullah 2021
		N/S		Butcher 2022
		N/S		Butcher 2023
		Society for Vascular Surgery (SVS) Vascular Quality Initiative (VQI) registry		VarkevisserRRB 2020
		International Severe Asthma Registry (ISAR)	Inflammatory and autoimmune disorders	FitzGerald 2020
		PURPLE (Pancreatic cancer: Understanding Routine Practice and Lifting End results) Pancreatic Cancer Registry	Oncology	Walpole 2023
		Fight Retinal Blindness Registry	Others	ChongTeo 2022
		Save Sight Registries (SSR) database		Kandel 2022
		Fight Retinal Blindness (FRB!) Registry		TeoKYC 2023
		Fight Retinal Blindness (FRB!) Registry		ChongTeo 2019

Names of identified databases (9)

Identified clinical registry databases from Singapore; One/more non-target countries (N=13)

Target countries	Database Type	Database Name	Disease Area	Study Details
Singapore; One/more non-target countries	Clinical registry	Fight Retinal Blindness! (FRB!) database	Others	Invernizzi 2019
		Fight Retinal Blindness! (FRB!) registry		Cornish 2023
		N/S		Gabrielle 2021
		N/S		TeoKYC 2021
		N/S		Vittorio 2020
		Fight Retinal Blindness! project database		Cornish 2021
		Fight Retinal Blindness! Registry		Gabrielle 2020
		N/S		Gabrielle 2022
		National Emergency Airway Registry for Neonates (NEAR4NEOS)		Foglia 2019
		National Emergency Airway Registry for Children (NEAR-4KIDS)		Kojima 2018
		National Emergency Airway Registry for Children (NEAR4KIDS)		Gradidge 2018
		National Emergency Airway Registry for Neonates (NEAR4NEOS)		Ozawa 2019
		Singapore Stroke Registry (SSR); Cognitive Rehabilitation Research Group Stroke Registry (CRRGSR)		Ng 2019

Names of identified databases (10)

Identified clinical registry, EMR/EHR; clinical registry, and EMR/EHR databases from cross-country collaboration studies (N=12)

Target countries	Database Type	Database Name	Disease Area	Study Details
Singapore; One/more non-target countries	EMR/EHR	CVD-REAL 2 Study; SingHealth Diabetes Registry	Cardiology and metabolic disorders	Kosiborod 2018
		Khoo Teck Puat Hospital (KTPH) and National University Hospital (NUH) databases from Singapore		Lu 2022
		N/S		deHoog 2018
		N/S		Tan 2022
		N/S		Vollema 2019
		N/S	Infectious diseases and vaccines	Bourgeois 2021
	EMR/EHR; Clinical registry	German Stroke Registry Endovascular Treatment	Others	Meyer 2021
Singapore; Pakistan; One/more non-target countries	Clinical registry	Asian CVT registry	Cardiology and metabolic disorders	Wasay 2019
Singapore; Philippines	Clinical registry	9 stroke registries from 6 Asian countries (China, Japan, Philippines, Singapore, South Korea and Taiwan)	Others	Wang 2019
		Stroke Acute Management with Urgent Risk-Factor Assessment and Improvement rtPA registry (SAMURAI); Thrombolysis Implementation and Monitor of acute ischemic Stroke (TIMS3); Philippines stroke registry; South Korean stroke registry; Singapore stroke registry; regional AIS registries (Changhua Christian Hospital stroke registry, Stroke Registry in Chang Gung Healthcare System, Shengyang Stroke registry, and Shanghai registry)		Wang 2021
Singapore; Vietnam; One/more non-target countries	Clinical registry	Extracorporeal Life Support Organization (ELSO) Registry	Infectious diseases and vaccines	Barbaro 2020
Vietnam; One/more non-target countries	Clinical registry	N/S	Cardiology and metabolic disorders	Rigatelli 2021
